# Proteomic and phosphoproteomic profiling in heart failure with preserved ejection fraction (HFpEF)

**DOI:** 10.3389/fcvm.2022.966968

**Published:** 2022-08-25

**Authors:** María Valero-Muñoz, Eng Leng Saw, Ryan M. Hekman, Benjamin C. Blum, Zaynab Hourani, Henk Granzier, Andrew Emili, Flora Sam

**Affiliations:** ^1^Whitaker Cardiovascular Institute, Boston University School of Medicine, Boston, MA, United States; ^2^Department of Biology, Boston University, Boston, MA, United States; ^3^Department of Biochemistry, Cell Biology and Genomics, Boston University, Boston, MA, United States; ^4^Center for Network Systems Biology, Boston University, Boston, MA, United States; ^5^Department of Cellular and Molecular Medicine, The University of Arizona, Tucson, AZ, United States

**Keywords:** HFpEF – heart failure with preserved ejection fraction, proteomics, phosphoproteomics, titin, mitochondria, metabolism, SIRT3

## Abstract

Although the prevalence of heart failure with preserved ejection fraction (HFpEF) is increasing, evidence-based therapies for HFpEF remain limited, likely due to an incomplete understanding of this disease. This study sought to identify the cardiac-specific features of protein and phosphoprotein changes in a murine model of HFpEF using mass spectrometry. HFpEF mice demonstrated moderate hypertension, left ventricle (LV) hypertrophy, lung congestion and diastolic dysfunction. Proteomics analysis of the LV tissue showed that 897 proteins were differentially expressed between HFpEF and Sham mice. We observed abundant changes in sarcomeric proteins, mitochondrial-related proteins, and NAD-dependent protein deacetylase sirtuin-3 (SIRT3). Upregulated pathways by GSEA analysis were related to immune modulation and muscle contraction, while downregulated pathways were predominantly related to mitochondrial metabolism. Western blot analysis validated SIRT3 downregulated cardiac expression in HFpEF vs. Sham (0.8 ± 0.0 vs. 1.0 ± 0.0; *P* < 0.001). Phosphoproteomics analysis showed that 72 phosphosites were differentially regulated between HFpEF and Sham LV. Aberrant phosphorylation patterns mostly occurred in sarcomere proteins and nuclear-localized proteins associated with contractile dysfunction and cardiac hypertrophy. Seven aberrant phosphosites were observed at the z-disk binding region of titin. Additional agarose gel analysis showed that while total titin cardiac expression remained unaltered, its stiffer N2B isoform was significantly increased in HFpEF vs. Sham (0.144 ± 0.01 vs. 0.127 ± 0.01; *P* < 0.05). In summary, this study demonstrates marked changes in proteins related to mitochondrial metabolism and the cardiac contractile apparatus in HFpEF. We propose that SIRT3 may play a role in perpetuating these changes and may be a target for drug development in HFpEF.

## Introduction

Heart failure (HF) is a clinical syndrome caused by abnormalities in the heart that limit its ability to fill or eject blood (1). Heart failure with preserved ejection fraction (HFpEF) is symptomatic clinical HF where left ventricular (LV) ejection fraction (EF) is preserved (LVEF ≥ 50%), and presently accounts for about 50% all HF clinical presentations. However, unlike HF with reduced EF (HFrEF), where LVEF is < 50%, there are limited evidence-based therapies for HFpEF (2–4). In addition to its escalating prevalence, HFpEF morbidity (5) and mortality (6) continues to increase. Central to HFpEF is the involvement of both cardiac and extra-cardiac abnormalities (7, 8). In contrast to HFrEF, HFpEF is highly associated with comorbidities and as such is a heterogenous multisystem disorder involving the heart, pulmonary, renal, adipose tissue, skeletal muscle, immune/inflammatory signaling and the vascular system (9, 10). Patients with HFpEF are generally older, more often female and have a predominance of comorbidities, such as hypertension, obesity, type 2 diabetes, atrial fibrillation, renal dysfunction, etc. (11, 12). However, the specific etiologies by which patients develop HFpEF are variable. Thus, a precision-based approach is needed to identify pathogenic mechanisms in HFpEF (10, 13).

Proteomic studies are powerful tools that allow for large-scale characterization of the entire protein phenotype in a biological system (14). Alterations in proteome patterns, such as global changes in protein expression and post-translational modifications (PTMs), are often indicative of marked changes in functional stages in health and disease (15). Thus, investigating the varying patterns of the proteome may provide insights into pathogenic pathways (16) and these protein signatures may facilitate rapid screening of the efficacy of novel treatments and aid in drug development (17, 18).

Previous proteomic studies have identified protein changes in dilated cardiomyopathy, atherosclerosis, and atrial fibrillation (19–23) and these types of studies likely provided a deeper mechanistic understanding of the molecular pathways in HF. For example, cardiac tissue from patients with HFrEF demonstrated protein modifications associated with cardiac metabolism, cardiac remodeling, and impaired cardiac contractility (24–27). Additionally, differentially regulated pathways by proteomic signatures were observed in HFrEF vs. HFpEF patients, which is consistent with the predominant view that the underlying pathophysiology in these two diseases are largely different, and thus the variable response to therapies. This difference is exemplified by Adamo et al., where blood samples from both HFrEF and HFpEF patients demonstrated increased growth factor signaling and increased angiogenesis markers, while proteomic signatures from only HFpEF patients showed increased humoral immunity and those from HFrEF patients showed increased extracellular matrix remodeling markers, consistent with active cardiac remodeling (28). These findings underscore the potential that high-performance proteomics, in combination with clinical assessment, may identify unique targets in specific groups of HF patients.

Although HFpEF is greatly impacted by the obesity and diabetes pandemic, hypertension remains the most prevalent and modifiable risk factor in HFpEF and is implicated in both its pathogenesis and prognosis (12, 29). Hypertensive HFpEF pathophysiology extends beyond the emphasis on LV hypertrophy development and diastolic dysfunction to impaired myocardial contractility, left atrial myopathy, cardiomyocyte remodeling, macro- and microvascular dysfunction, to systemic inflammation, fibrosis, and collagen deposition. However, despite this knowledge a paucity of therapies exists for HFpEF. Here, we applied a deep quantitative proteomics and phosphoproteomic profiling approach to identify molecular protein signatures that are altered in HFpEF in a well characterized murine model of hypertension-associated HFpEF, the *SAUNA* model (SAlty drinking water/Unilateral Nephrectomy/Aldosterone), which recapitulates the human HFpEF phenotype (30–37) (Supplementary Figure 1). Using an unbiased and comprehensive analysis, we report a systematic, large-scale study of pathway, metabolic and organelle level changes that occur in the left ventricle of this HFpEF murine model.

## Material and methods

All procedures related to the handling and surgery of the mice conformed to the *Guide for the Care and Use of Laboratory Animals* published by the United States National Institutes of Health and were approved by the Institutional Animal Care and Use Committee at Boston University School of Medicine.

### *SAUNA* model of HFpEF

As previously described (30–33, 35–37), eight-week-old male C57BL/6J mice (Jackson Laboratories) were anesthetized with 80–100 mg/Kg ketamine and 5–10 mg/Kg xylazine intraperitoneally. Mice (20–25 g) then underwent uninephrectomy, received either a continuous infusion of saline (Sham) or *d*-aldosterone (0.30 μg/h, Sigma-Aldrich, St. Louis, MO, United States; HFpEF) for 4 weeks via osmotic minipumps (Alzet, Durect Corp., Cupertino, CA, United States) and were maintained on 1% sodium chloride drinking water.

### Physiological measurements

Blood pressure and echocardiographic measurements were performed at the end of the 4 weeks. Systolic blood pressure was measured using a non-invasive tail-cuff blood pressure analyzer (BP-2000 Blood Pressure Analysis System; Visitech Systems Inc., Apex, NC, United States). Transthoracic echocardiography was performed using a Vevo770 High-Resolution *in vivo* Micro-Imaging System and a Real-Time Micro Visualization 707B Scanhead (VisualSonic Inc., Toronto, ON, Canada) as previously described (33). Briefly, interventricular septum wall thickness (IVST), left ventricle (LV) posterior wall thickness (LVPWT), LV end-diastolic diameter (LVEDD), LV end-systolic diameter (LVESD), and LV ejection fraction (LVEF) were measured. As a measure of systolic function and cardiac contractility fractional shortening (FS) was calculated as follows (LVEDD-LVESD/LVEDD) × 100. Total wall thickness (TWT) was derived from an average of the IVST and LVPWT. Relative wall thickness (RWT) was calculated as 2× LVPWT/LVEDD. LV mass was calculated using the formula described by Kiatchoosakun et al. (38). As diastolic function is sensitive to heart rate (HR) and loading conditions, HR was maintained at ∼350 bpm during these measurements (39). Pulse wave measurements were then recorded and analyzed blinded to group.

### Histopathological analyses

Paraffin-embedded sections (5 μm) of the mid-LV were stained with hematoxylin and eosin (H&E, Sigma-Aldrich) to measure LV cardiac myocyte cross-sectional area. Microscopy images (BZ-9000 BioRevo microscope, Keyence Corp. of America, Itasca, IL, United States) were analyzed blinded to group identity using ImageJ measuring software (National Institutes of Health, Bethesda, MD, United States).

### Tissue sample preparation for proteomics and phosphoproteomics

Left ventricle samples from 4 mice/group were processed as previously described (22, 40–42). Briefly, freshly thawed samples were homogenized on ice in with a mixer mill MM 400 (Retsch USA Verder Scientific Inc., Newtown, PA, United States) in 10 volumes of 8 M urea, 50 mM ammonium bicarbonate, 2 mM dithiothreitol, and protease and phosphatase inhibitor cocktails (Roche Applied Science, Indianapolis, IN, United States). Tissue homogenate was then sonicated with a probe sonicator (Branson Ultrasonics Corporation, North Billerica, MA, United States) and centrifuged. After centrifugation, supernatant was decanted and total protein in each sample was determined using a modified “microtiter plate” version of the Bradford assay (Sigma-Aldrich). For phosphoproteomics experiments, aliquots containing 300 μg of protein were alkylated with 5 mM iodoacetamide for additional 45 min at room temperature in the dark. Samples were then diluted eight-fold with 50 mM ammonium bicarbonate and digested overnight with sequencing-grade trypsin (#90057, Thermo Fisher Scientific Inc., Waltham, MA, United States). Digestion was stopped by acidification to a final concentration of 1% (v/v) formic acid and the peptide solutions were desalted using disposable C18 Sep-Pak syringes (Waters Corporation, Milford, MA, United States) and lyophilized to dryness following manufacturer’s instructions.

### Tandem mass tag (TMT) labeling

Peptide concentrations were determined by a colorimetric peptide assay kit (Thermo Fisher Scientific Inc., Waltham, MA, United States) and an aliquot of 100 μg was placed in 100 μl of 100 mM triethylammonium bicarbonate. Peptides were labeled with 0.4 mg of TMT label (TMT10plex™ Isobaric Label Reagent Set, Thermo Fisher Scientific Inc., Waltham, MA, United States). All samples were labeled in the same TMT-batch, representing reporter tags 126C, 127N, 127C, 128C, 129N, 129C, 130N, and 131N. Labeled samples were pooled, and 95% was set aside for phosphopeptide enrichment. The remaining 5% of labeled peptides and the phosphopeptide enriched samples were analyzed separately by mass spectrometry.

### Phosphopeptide enrichment

Phosphopeptides were selectively enriched by binding to titanium dioxide (TiO_2_) beads (Titansphere Phos-TiO Bulk 10 μm, GL Sciences, Tokyo, Japan) (43). Briefly, peptides were resuspended in 200 μl 80% acetontirile, 6% trifluoroacetic acid and incubated for 10 min with 10 μl of slurry containing TiO_2_ beads. Unbound peptides and supernatant were decanted, and the beads were washed three times with a wash buffer containing 50% acetonitrile and 1% trifluoroacetic acid. After final decanting, the beads were incubated for 10 min with elution solution containing 25% ammonium hydroxide and 50% acetonitrile and the eluate was carefully removed and dried prior to mass spectrometry analysis.

### Mass spectrometry analysis

Tryptic peptide mixtures and enriched phosphopeptides were analyzed by nano-scale high-performance liquid chromatography (Proxeon EASY-Nano system, Thermo Fisher Scientific Inc., Waltham, MA, United States) and online nano electrospray ionization tandem mass spectrometry (Q-Exactive HF-X mass spectrometer; Thermo Fisher Scientific Inc., Waltham, MA, United States). Briefly, samples were loaded in aqueous 0.1% (v/v) formic acid via a trap column (75 μm i.d. × 2 cm, Acclaim PepMap100 C18 3 μm, 100 Å, Thermo Fisher Scientific) and peptides were resolved over an Easy-Spray analytical column (50 cm × 75 μm ID, PepMap RSLC C18, Thermo Fisher Scientific) by an increasing mobile phase B. Mobile phase A consisted of 2% acetonitrile and 0.1% formic acid, and organic phase B contained 80% acetonitrile and 0.1% formic acid. Reverse phase separation was performed over 120 min at a flow rate of 300 nl/min. Eluted peptides were ionized directly into the mass spectrometer using a nanospray ion source. The mass spectrometer was operated in positive ion mode with a capillary temperature of 300 C, and with a potential of 2,100 V applied to the frit. Tandem mass spectrometry (MS/MS) was performed using high-energy collision-induced disassociation and 10 MS/MS data-dependent scans (45,000 resolution) were acquired in profile mode alongside each profile mode full-scan mass spectra (120,000 resolution) as reported previously (44). The automatic gain control (AGC) for MS scans was 1 × 10^6^ ions with a maximum fill time of 60 ms. The AGC for MS/MS scans was 3 × 10^4^, with 80 ms maximum injection time, 0.1 ms activation time, and 33% normalized collision energy. To avoid repeated selection of peptides for MS/MS a dynamic exclusion list was enabled to exclude all fragmented ions for 60 s.

### Protein identification

Data files (RAW format) were searched using the standard workflow of MaxQuant (version 1.3.0.5)^1^ under standard settings using the entire Swiss-Prot mouse database^2^ downloaded January 24, 2019, allowing for two missed trypsin cleavage sites, carbamidomethylation of cysteine (fixed) and variable oxidation of methionine, protein N-terminal acetylation and phosphorylation of STY residues. Precursor ion tolerances were 20 ppm for first search and 4.5 ppm for a second search. The MS/MS peaks were de-isotoped and searched using a 20-ppm mass tolerance. A stringent false discovery rate threshold of 1% was used to filter candidate peptide, protein, and phosphosite identifications. The datasets generated for this study have been deposited and publicly available at the PRIDE Archive, proteomics data repository (European Bioinformatics Institute, European Molecular Biology Laboratory) with the data set identifier PXD033501.

### Bioinformatics analysis

The searched intensity data were filtered, normalized, and clustered using *Omics Notebook* (45). Filtering was performed to remove any proteins or phosphopeptides not quantified in at least 70 percent of samples, with 2,905 and 281 proteins and phosphopeptides passing the filter, respectively. After filtering, both datasets showed low levels of sparsity and no missing value imputation was performed. The LIMMA R package was used for LOESS normalization and differential expression analysis (46). A combined ranked list for both sets was generated where duplicate gene entries were removed to keep the entry with the highest absolute rank value.

### GSEA analysis

Gene Set Enrichment analysis (GSEA) software from the fgsea R package was used to compute gene set enrichment after ranking proteins by differential expression in HFpEF vs. Sham (45, 47, 48). Briefly, GSEA was used in rank mode along with gene sets downloaded from the Bader Lab (Mouse_GOBP_AllPathways_no_GO_iea_October_01_2018_ symbol.gmt)^3^ (49, 50). GSEA results were visualized using the Enrichment Map app (Version 3.1) in Cytoscape (Version 3.6.1) and highly related pathways were grouped into a theme and labeled by AutoAnnotate (version 1.2). For the merged gene set analyses, we applied an enrichment *P* < 0.01 and FDR ≤ 0.1 cutoffs and calculated overlap between gene set annotations using a combination of Jaccard and overlap coefficients with a cutoff of 0.375.

### Titin isoform analysis

Additional studies were performed to investigate changes in titin isoforms in HFpEF. Briefly, LV protein lysates from Sham (*N* = 7) and HFpEF (*N* = 11) mice were extracted and electrophoresed in 1% agarose gels using a SE600X vertical gel system (Hoefer Inc., Holliston, MA, United States) as previously described (51). Gels were run at 15 mA constant current, stained with Neuhoff’s Coomassie (52), and then scanned using Epson Perfection V750 PRO scanner (Epson America Inc., Los Alamitos, CA, United States) and analyzed using One-D scan EX analysis software (Scanalytics Inc., Rockville, MD, United States). The integrated optical density of titin and total myosin heavy chain (MHC) was determined as a function of the slope of the linear range between integrated optical density and loaded volume (53). The expression of compliant N2BA titin, stiffer N2B titin and total titin (TT) was normalized to the expression of total MHC. The expression of titin degraded product (T2) was normalized to the TT expression.

### SIRT3 immunoblotting analysis

Protein lysates were extracted from LV tissue using in ice-cold RIPA buffer as previously described (54). Equal amounts of protein were then subjected to electrophoresis in SDS-polyacrylamide gel under reducing conditions and blotted to polyvinylidene difluoride (PVDF) membranes using the Bio-Rad Transblot Turbo Transfer System (Hercules, CA, United States). The membranes were blocked in 5% BSA, 0.1% Tween-20 in tris-buffered saline for 1 h at room temperature and then incubated overnight at 4°C with rabbit anti-SIRT3 antibody (Cell Signaling Technology, Inc., Danvers, MA, United States, #5490; 1:1.000). Membranes were then washed with tris-buffered saline and incubated with respective horseradish peroxidase (HRP)-conjugated secondary antibodies for 1 h in room temperature: anti-rabbit antibody (R&D system, HAF008; 1:5,000). Immune complexes were detected with the enhanced chemiluminescence ECL detection system (Bio-Rad, #1705060) in the ImageQuant LAS 4000 biomolecular imaging system (GE Healthcare, Pittsburgh, PA, United States). The intensity of bands for each protein was normalized to the loading control mouse anti-GAPDH (Abcam, Ab8245; 1:10.000).

### Statistical analysis

Proteomics and phosphoproteomics differential analysis were based on a moderated *t*-test and performed using R: A language and environment for statistical computing (R Foundation for Statistical Computing, Vienna, Austria) (45, 55). For histology analysis, titin isoform studies and SIRT3 expression, data are shown as mean ± SEM and statistical significance of differences was assessed using the Student’s *t*-test (two sided). In those cases when data were not sampled as a normal distribution, non-parametric Mann–Whitney *U* test was used. *P* ≤ 0.05 values were considered significant. These statistical tests were performed using GraphPad Prism software (GraphPad Software Inc., La Jolla, CA, United States).

## Results

### Mouse model of HFpEF

As previously described (30–33, 35–37), salty drinking water, unilateral nephrectomy, and chronic exposure to aldosterone (*SAUNA*) induced hypertension associated HFpEF in mice. Compared to Sham, HFpEF mice demonstrated a moderate increase in systolic blood pressure (137.8 ± 7.0 mmHg vs. 115.4 ± 6.0 mmHg; *P* < 0.05), lung congestion (4.5 ± 0.1 vs. 4.0 ± 0.1 *P* < 0.01), and LV hypertrophy, measured by the LV weight-to-total body weight ratio (3.7 ± 0.1 mg/g vs. 3.3 ± 0.1 mg/g; *P* < 0.05). Additionally, cardiomyocyte size was increased 1.2-fold in HFpEF mice vs. Sham; *P* < 0.05 (Supplementary Figure 2).

Echocardiography demonstrated preserved LVEF and increased LV mass (107.5 ± 4.9 mg vs. 78.2 ± 7.9 mg in Sham; *P* < 0.05; Table 1). Wall thickness was significantly increased in HFpEF and there was evidence of concentric hypertrophy, as demonstrated by the increased relative wall thickness (0.7 ± 0.1 vs. 0.5 ± 0.0 in Sham; *P* < 0.005). As previously shown (33), LV end-systolic dimensions and end-diastolic dimensions were also decreased in HFpEF (Table 1). HFpEF mice had impaired diastolic function, characterized by an increase in isovolumetric relaxation time (24.3 ± 2.6 ms vs. 14.4 ± 1.6 ms in Sham; *P* < 0.05).

**TABLE 1:** Characteristics and echocardiographic parameters of HFpEF (*SAUNA*) mice 4 weeks after *d*-Aldosterone or saline (Sham) infusion.

	HFpEF	Sham
Systolic blood pressure (mmHg)	137.8 ± 7.0*	115.4 ± 6.0
Wet-to-dry lung ratio	4.5 ± 0.1**	4.0 ± 0.1
Heart weight-to-body weight (mg/g)	3.7 ± 0.1*	3.3 ± 0.1
**Left ventricle structure and function**		
LV mass (mg)	107.5 ± 4.9*	78.6 ± 7.9
Total wall thickness (mm)	1.0 ± 0.0***	0.8 ± 0.1
Posterior wall thickness (mm)	1.0 ± 0.1*	0.8 ± 0.1
Relative wall thickness	0.7 ± 0.1***	0.5 ± 0.0
LV end-systolic diameter (mm)	1.1 ± 0.2*	1.6 ± 0.1
LV end-diastolic diameter (mm)	3.0 ± 0.2	3.3 ± 0.1
LV ejection fraction (%)	91.1 ± 1.3	83.1 ± 3.0
LV fractional shortening	62.1 ± 2.3	52.0 ± 3.5
E/A	1.9 ± 0.2	1.7 ± 0.2
Early filling deceleration time (ms)	21.0 ± 3.0	17.6 ± 2.6
Isovolumetric relaxation time (ms)	24.3 ± 2.6*	14.4 ± 1.6

Data are expressed as mean ± SEM. A, peak late transmitral flow velocity; E, peak early transmitral flow velocity; LV, left ventricular (N = 5 mice/group), *P < 0.05 vs. Sham; **P < 0.01 vs. Sham; ***P < 0.005 vs. Sham. Statistical analysis by two-tailed Student’s t-test.

*Comparison to human HFpEF*: Recently, two clinical scores (HFA-PEFF and H2FPEF) were developed to standardize the clinical diagnosis of human HFpEF. However, a discrepancy exists between these scores (56). The H2FPEF score largely includes clinical parameters whereas the HFA-PEFF score includes predominantly echocardiographic measures and natriuretic peptides. The HFA-PEFF score can rule in human HFpEF with high specificity (93%) and positive predictive value (98%) when the score is high (5–6 points) (57). As such, the translational utility of the HFpEF *SAUNA* mouse model was demonstrated in the context of this HFpEF score with a HFA-PEFF score of ≥ 6 as described by Withaar et al. (58), where a score of ≥ 5 is a high probability of clinical HFpEF.

### Proteome profile of the left ventricle in HFpEF

**FIGURE 1:**
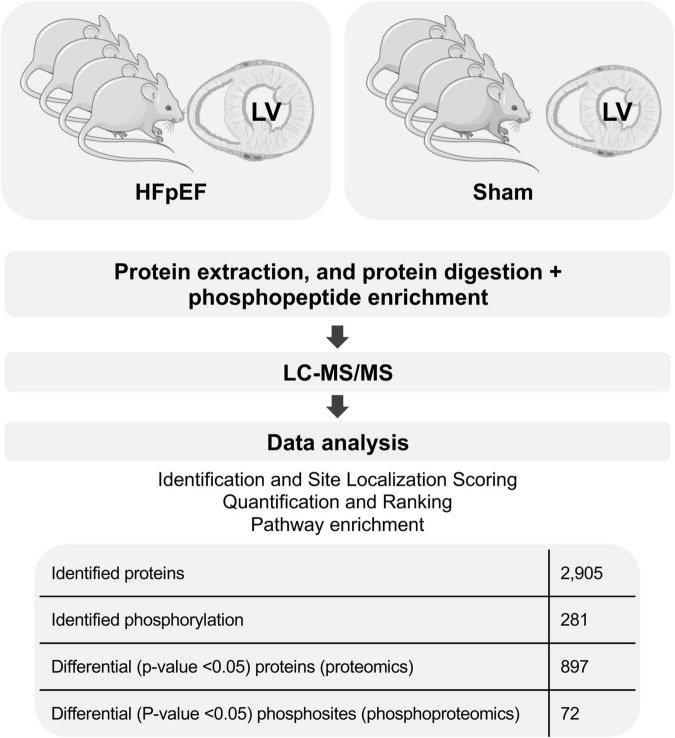
General proteomics and phosphoproteomics workflow. The figure was partly generated using Servier Medical Art, provided by Servier, licensed under a Creative Commons Attribution 3.0 unported license.

To achieve comprehensive evaluation of the cardiac signaling that is seen in HFpEF, a global quantitative proteome and phosphoproteome profile was performed in LV cardiac tissue obtained from HFpEF mice and their respective Shams (*N* = 4 mice/group; Figure 1).

**FIGURE 2:**
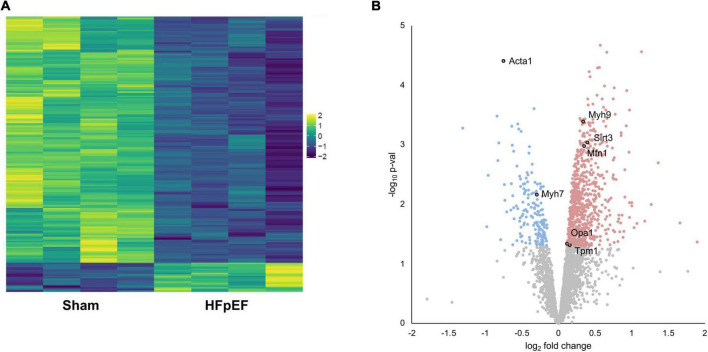
Proteomics analysis. **(A)** Heatmap of the differentially expressed proteins. **(B)** Volcano plot presenting log-transformed *p*-values (*t*-test) associated with individual significantly altered proteins plotted against log-transformed fold change in abundance between the left ventricles in Sham and HFpEF mice. Blue and red dots represent up-regulation and down-regulation in HFpEF (*N* = 4) vs. Sham (*N* = 4), respectively.

Proteomics analysis found a total of 2,905 identified proteins that were then used for comparative analysis (Supplementary Table 1). Among them, 897 proteins were differentially expressed between HFpEF and Sham LV, with 19% of these being predominantly higher in HFpEF than in Sham (*P* < 0.05; Figures 2A,B).

Systematic evaluation of the datasets revealed abundant changes in sarcomeric proteins, namely skeletal alpha (α)-actin (ACTA1; *P* = 0.000039), beta (β)-myosin heavy chain (MYH7; *P* = 0.006963), myosin heavy chain 9 (MYH9; *P* = 0.000408), tropomyosin alpha (α)-1 chain (TPM1; *P* = 0.048698); the mitochondria-related proteins mitofusin 1 (MFN1; *P* = 0.001059), mitochondrial dynamin like GTPase (*aka* optic atrophy protein 1, OPA1; *P* = 0.046441) and transcription factor A mitochondrial (TFAM; *P* = 0.005837); and the NAD-dependent protein deacetylase sirtuin-3 (SIRT3; *P* = 0.000914), recently implicated in cardiac function and cardiac stress responsiveness in HFpEF (59, 60) (Figure 2B and Supplementary Table 1).

### Impaired mitochondrial function and oxidative metabolism of energy substrates in HFpEF

**FIGURE 3:**
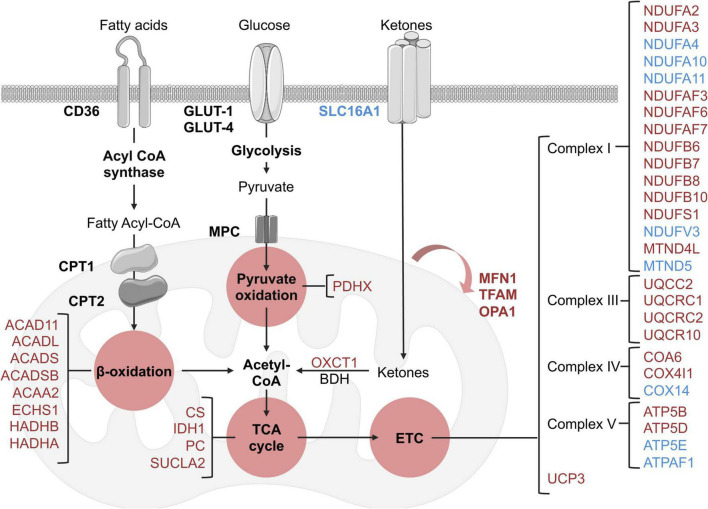
Schematic overview of identified metabolic and mitochondrial related targets in HFpEF mice in the proteome dataset. Blue and red represent significant (*P* < 0.05) up-regulation and down-regulation in HFpEF vs. Sham, respectively. Black targets are unchanged.

There was an extensive *reduction* in the abundance of proteins involved in cardiac metabolism in the LV of HFpEF mice, including the oxidation of free fatty acid (FFA), pyruvate, and ketone bodies. Significant changes are summarized in Figure 3. These include:

(I) β-oxidation related enzymes, implicated in FFA metabolism to acetyl-CoA, such as acyl-CoA dehydrogenase (ACAD) family member 11 (ACAD11; *P* = 0.00002), long-chain specific ACAD (ACADL; *P* = 0.00449), short-chain specific ACAD (ACADS; *P* = 0.00896), short-branched chain specific ACAD (ACADSB; *P* = 0.011317), 3-ketoacyl-CoA thiolase (ACAA2, *P* = 0.00609), enoyl-CoA hydratase (ECHS1, *P* = 0.02647), hydroxyacyl-CoA dehydrogenase trifunctional multienzyme complex (HADH) beta (β)-subunit (HADHB, *P* = 0.00729) and HADH alpha (α)-subunit (HADHA, *P* = 0.03235).

(II) the pyruvate oxidation enzyme pyruvate dehydrogenase X component (PDHX, *P* = 0.00961), which is part of the pyruvate dehydrogenase complex that catalyzes pyruvate to acetyl-CoA; and

(III) the ketone metabolism enzyme succinyl-CoA:3-keto- acid coenzyme A transferase 1 (OXCT1, *P* = 0.01237), which catalyzes ketone bodies and produces acetyl-CoA for the tricarboxylic acid (TCA) cycle.

These cumulative results suggest that the energy substrates for mitochondrial oxidative metabolism may be inefficient in HFpEF. Interestingly, although there were no significant alterations in the protein signature of fatty acid and glucose transporters (CD36 and GLUT1 and 4, respectively) in HFpEF, there was an upregulation of the ketone bodies transporter monocarboxylate transporter 1 (SLC16A1, *P* = 0.00376) in the LV of HFpEF.

Additional analysis revealed that the mitochondrial proteins involved in the TCA cycle were also significantly decreased in the LV of HFpEF mice. These mitochondrial enzymes, namely citrate synthase (CS, *P* = 0.008068), succinyl-CoA ligase beta subunit (SUCLA2, *P* = 0.02523), isocitrate dehydrogenase (IDH1, *P* = 0.00105) and pyruvate carboxylase (PC, *P* = 0.00206), are required to catalyze acetyl-CoA and produce essential intermediates for the biosynthesis process, and most importantly, high energy molecules such as nicotinamide adenine dinucleotide (NADH) and flavin adenine dinucleotide (FADH_2_) for the electron transport chain (ETC). Subsequent analysis then showed that 27 proteins involved in the ETC (namely the respiratory complex I, III, IV, and V) were also differentially expressed between HFpEF and Sham. Of these 27 proteins, 19 proteins were significantly reduced in the HFpEF, suggesting impaired ETC, which was consistent with an additional reduction of the uncoupling protein 3 (UCP3, *P* = 0.02765).

Lastly, additional proteins involved in mitochondrial biogenesis (transcription factor A, TFAM, *P* = 0.00584) and fusion (mitofusin-1, MFN1, *P* = 0.001056 and dynamin-like 120 kDa protein, OPA1, *P* = 0.04644) were similarly decreased in the LV tissue from HFpEF mice.

These findings (Figure 3) suggest that mitochondrial dysfunction may lead to inefficient metabolism of energy substrates, possibly contributing to an energy deficit and thus affecting cardiac function in HFpEF.

### Pathway enrichment analysis

Pathway enrichment analyses of the proteomics and phosphoproteomics combined datasets were performed by means of GSEA, which detects biology-driven gene sets of canonical pathways from databases of molecular signatures (61). These analyses revealed that the most relevant and over-represented (enriched) biological annotations in the LV from HFpEF to be: (I) processes involving immune system modulation, (II) cardiac muscle cell development and differentiation, and (III) muscle contraction (Table 2). These processes included positive regulation of cytokine production (GO:0001819; *P* = 0.0000), striated muscle contraction (Wikipathway; *P* = 0.0000), positive regulation of adaptive immune response (GO:0002821; *P* = 0.00578), cardiac muscle cell development (GO:0055013; *P* = 0.03158) and cardiac muscle cell differentiation (GO: 0055007; *P* = 0.03571). In contrast, the downregulated pathways were related to a multitude of GO terms associated with cellular metabolism (Table 3). This is consistent with the earlier data from Figure 3, where pathways and processes involving acetyl-CoA metabolic process (GO:0006084, *P* = 0.00000), fatty acid metabolic process (GO:0006631, *P* = 0.00000), acyl-CoA biosynthesis process (GO:0071616, *P* = 0.0000), fatty acid oxidation (GO:0019395, *P* = 0.00111), coenzyme metabolic process (GO:0006732, *P* = 0.01015) were significantly reduced in HFpEF.

**TABLE 2:** Biological annotations terms enriched in significantly up-regulated proteins of the proteome dataset.

Name	Group	*P*-value	Size	ES
**Positive regulation of cytokine production**	GO:0001819	0.00000	57	−0.34
Pallium development	GO:0021543	0.00000	32	−0.38
Platelet degranulation	Reactome pathway	0.00000	56	−0.33
Response to elevated platelet cytosolic ca2 +	Reactome pathway	0.00000	58	−0.33
Signaling by ROBO receptors	Reactome pathway	0.00000	98	−0.26
**Striated muscle contraction**	Wikipathway	0.00000	30	−0.42
**Positive regulation of adaptive immune response**	GO:0002821	0.00578	18	−0.47
Regulation of adaptive immune response based on somatic recombination of immune receptors built from immunoglobulin superfamily domains	GO:0002822	0.00585	18	−0.47
Positive regulation of wound healing	GO:0090303	0.00595	16	−0.51
Intrinsic pathway for apoptosis	Reactome pathway	0.00633	15	−0.54
Positive regulation of response to wounding	GO:1903036	0.00671	20	−0.44
Integrin pathway	Biocarta pathway	0.00690	23	−0.39
Nucleus organization	GO:0006997	0.00893	31	−0.36
Foxo pathway	PID pathway	0.01500	16	−0.48
G2 m checkpoints	Reactome pathway	0.01754	52	−0.28
Coagulation	Hallmark Pathway	0.01818	54	−0.29
Complement and coagulation cascades	Wikipathway	0.01829	21	−0.45
Cerebral cortex development	GO:0021987	0.02143	27	−0.39
Rho GTPases activate PKNs	Reactome pathway	0.02158	25	−0.40
Positive regulation of adaptive immune response based on somatic recombination of immune receptors built from immunoglobulin superfamily domains	GO:0002824	0.02222	17	−0.47
**Regulation of adaptive immune response**	GO:0002819	0.02367	19	−0.46
Spermatid development	GO:0007286	0.02717	16	−0.44
Spermatid differentiation	GO:0048515	0.02924	16	−0.44
Fc epsilon receptor signaling	Reactome pathway	0.02985	50	−0.27
**Cardiac muscle cell development**	GO:0055013	0.03158	38	−0.31
Regulation of production of molecular mediator of immune response	GO:0002700	0.03550	17	−0.42
**Cardiac muscle cell differentiation**	GO:0055007	0.03571	41	−0.30
Activation of MAPK activity	GO:0000187	0.03593	21	−0.38
**Cardiac cell development**	GO:0055006	0.03659	38	−0.31
Regulation of blood coagulation	GO:0030193	0.04380	25	−0.35
Regulation of expression of SLITS and ROBOS	Reactome pathway	0.04762	82	−0.25

ES, enrichment score. Results are sorted by the nominal P-value in an ascending order.

**TABLE 3:** Biological annotations terms significantly enriched in down-regulated proteins of the proteome dataset.

Name	Group	*P*-value	Size	ES
Regulation of tp53 activity	Reactome pathway	0.00000	23	0.60
Purine nucleoside bisphosphate metabolic process	GO:0034032	0.00000	43	0.51
**Acetyl-coA metabolic process**	GO:0006084	0.00000	16	0.64
Negative regulation of lipid metabolic process	GO:0045833	0.00000	17	0.63
Ribonucleoside bisphosphate metabolic process	GO:0033875	0.00000	43	0.51
Monocarboxylic acid catabolic process	GO:0072329	0.00000	46	0.53
Nucleoside bisphosphate metabolic process	GO:0033865	0.00000	43	0.51
**Fatty acid metabolic process**	GO:0006631	0.00000	91	0.48
Carboxylic acid catabolic process	GO:0046395	0.00000	78	0.46
Monocarboxylic acid metabolic process	GO:0032787	0.00000	138	0.45
Organic acid catabolic process	GO:0016054	0.00000	78	0.46
**Acyl-CoA biosynthetic process**	GO:0071616	0.00000	15	0.67
Thioester biosynthetic process	GO:0035384	0.00000	15	0.67
Sulfur compound metabolic process	GO:0006790	0.00000	99	0.44
Carboxylic acid metabolic process	GO:0019752	0.00000	259	0.41
Oxoacid metabolic process	GO:0043436	0.00000	264	0.41
Organic acid metabolic process	GO:0006082	0.00000	268	0.41
Cellular monovalent inorganic cation homeostasis	GO:0030004	0.00000	17	0.66
Response to nitrogen compound	GO:1901698	0.00000	196	0.39
Small molecule metabolic process	GO:0044281	0.00100	463	0.34
Thioester metabolic process	GO:0035383	0.00109	39	0.53
**Fatty acid oxidation**	GO:0019395	0.00111	38	0.52
Positive regulation of ion transmembrane transporter activity	GO:0032414	0.00111	34	0.51
Cilium assembly	GO:0060271	0.00115	22	0.59
Protein trimerization	GO:0070206	0.00121	17	0.66
Sulfur compound biosynthetic process	GO:0044272	0.00229	26	0.58
Protein dephosphorylation	GO:0006470	0.00231	25	0.59
Protein localization	Reactome pathway	0.00310	84	0.42
**Acyl-CoA metabolic process**	GO:0006637	0.00327	39	0.53
**Fatty acid catabolic process**	GO:0009062	0.00328	39	0.51
Activation of GTPase activity	GO:0090630	0.00362	16	0.61
Response to oxygen-containing compound	GO:1901700	0.00400	256	0.35
**Fatty acid beta-oxidation**	GO:0006635	0.00439	31	0.54
**Lipid oxidation**	GO:0034440	0.00443	38	0.52
Monovalent inorganic cation homeostasis	GO:0055067	0.00473	20	0.62
Laminin interactions	Reactome pathway	0.00486	15	0.63
Small molecule catabolic process	GO:0044282	0.00509	110	0.40
**Lipid modification**	GO:0030258	0.00536	45	0.50
Dephosphorylation	GO:0016311	0.00553	34	0.52
Ion channel transport	Reactome pathway	0.00553	34	0.51
Metabolism of water-soluble vitamins and cofactors	Reactome pathway	0.00559	32	0.54
Response to organonitrogen compound	GO:0010243	0.00604	178	0.38
Cellular amino acid metabolic process	GO:0006520	0.00609	98	0.41
Positive regulation of transporter activity	GO:0032411	0.00661	37	0.51
Cilium organization	GO:0044782	0.00685	23	0.58
Nucleoside bisphosphate biosynthetic process	GO:0033866	0.00823	19	0.56
Neurotransmitter transport	GO:0006836	0.00894	27	0.54
Positive regulation of ion transmembrane transport	GO:0034767	0.00966	49	0.46
Cell projection assembly	GO:0030031	0.00968	49	0.46
Long-chain fatty acid metabolic process	GO:0001676	0.00980	17	0.60
Response to drug	GO:0042493	0.01006	137	0.38
Cell projection organization	GO:0030030	0.01006	155	0.37
**Coenzyme metabolic process**	GO:0006732	0.01015	126	0.38
**Fatty acid metabolism**	Reactome pathway	0.01053	72	0.42
**Mitochondrial fatty acid beta-oxidation**	Reactome pathway	0.01114	28	0.52
Cellular response to hormone stimulus	GO:0032870	0.01148	75	0.43
Pyrimidine-containing compound metabolic process	GO:0072527	0.01214	17	0.59
Cellular response to oxygen levels	GO:0071453	0.01350	26	0.52
Plasma membrane bounded cell projection assembly	GO:0120031	0.01609	47	0.46
Purine nucleoside bisphosphate biosynthetic process	GO:0034033	0.01667	19	0.56
Negative regulation of cellular response to TGFbeta stimulus	GO:1903845	0.01914	15	0.58
Ribonucleoside bisphosphate biosynthetic process	GO:0034030	0.01932	19	0.56
Transmission across chemical synapses	Reactome pathway	0.01967	45	0.45
Nephron development	GO:0072006	0.01975	15	0.59
Cellular response to endogenous stimulus	GO:0071495	0.02018	163	0.35
Positive regulation of transmembrane transport	GO:0034764	0.02030	62	0.42
Plasma membrane bounded cell projection organization	GO:0120036	0.02113	149	0.36
Cellular response to organic substance	GO:0071310	0.02200	323	0.33
Response to organic substance	GO:0010033	0.02200	448	0.32
Branched-chain amino acid catabolism	Reactome pathway	0.02241	21	0.54
Regulation of coenzyme metabolic process	GO:0051196	0.02281	18	0.56
Neuronal system	Reactome pathway	0.02318	59	0.42
Cellular response to inorganic substance	GO:0071241	0.02341	34	0.48
Negative regulation of transmembrane receptor protein serine/threonine kinase signaling pathway	GO:0090101	0.02392	19	0.55
FCgamma receptor dependent phagocytosis	Reactome pathway	0.02540	25	0.51
Fatty acid biosynthetic process	GO:0006633	0.02549	23	0.52
Cellular response to nitrogen compound	GO:1901699	0.02554	108	0.38
Negative regulation of TGFbeta receptor signaling pathway	GO:0030512	0.02599	15	0.58
Positive regulation of cation transmembrane transport	GO:1904064	0.02612	46	0.45
Negative regulation of organelle organization	GO:0010639	0.02764	87	0.39
Regulation of muscle organ development	GO:0048634	0.02772	34	0.46
Dicarboxylic acid metabolic process	GO:0043648	0.02793	42	0.46
Positive regulation of striated muscle tissue development	GO:0045844	0.02818	21	0.52
Positive regulation of muscle tissue development	GO:1901863	0.02904	21	0.52
Cellular amino acid biosynthetic process	GO:0008652	0.03012	17	0.56
Positive regulation of muscle organ development	GO:0048636	0.03030	21	0.52
Signal release	GO:0023061	0.03111	26	0.49
Heterotrimeric G-protein signaling pathway-GI alpha and GS alpha mediated pathway	Panther pathway	0.03222	22	0.50
Response to endogenous stimulus	GO:0009719	0.03307	215	0.33
Protein complex oligomerization	GO:0051259	0.03313	163	0.36
Neurotransmitter secretion	GO:0007269	0.03410	16	0.57
Signal release from synapse	GO:0099643	0.03431	16	0.57
Regulation of transporter activity	GO:0032409	0.03434	71	0.40
Regulation of transmembrane transporter activity	GO:0022898	0.03441	68	0.40
Cellular response to lipid	GO:0071396	0.03470	67	0.40
Regulation of TGFbeta receptor signaling pathway	GO:0017015	0.03477	21	0.52
Regulation of transmembrane receptor protein serine/threonine kinase signaling pathway	GO:0090092	0.03528	35	0.46
NABA basement membranes	MSIGDB	0.03534	19	0.53
Coenzyme biosynthetic process	GO:0009108	0.03560	67	0.40
Cellular response to chemical stimulus	GO:0070887	0.03600	417	0.31
Response to organic cyclic compound	GO:0014070	0.03858	123	0.36
Transition metal ion transport	GO:0000041	0.03943	19	0.54
Regulation of striated muscle tissue development	GO:0016202	0.04013	34	0.46
Response to ammonium ion	GO:0060359	0.04152	23	0.49
Positive regulation of ion transport	GO:0043270	0.04280	73	0.39
Opioid signaling	Reactome pathway	0.04282	22	0.52
Cell-cell adhesion	GO:0098609	0.04366	66	0.40
Positive regulation of sodium ion transport	GO:0010765	0.04380	16	0.55
Regulation of muscle tissue development	GO:1901861	0.04402	34	0.46
Blood vessel morphogenesis	GO:0048514	0.04516	48	0.42
Regulation of NIK/NF-kappaB signaling	GO:1901222	0.04535	19	0.52
Regulation of response to drug	GO:2001023	0.04642	15	0.55
Alpha-amino acid metabolic process	GO:1901605	0.04674	55	0.41
Negative regulation of cell proliferation	GO:0008285	0.04689	84	0.37
Activation of cysteine-type endopeptidase activity involved in apoptotic process	GO:0006919	0.04711	20	0.50
Integrin signaling pathway	MSIGDB	0.04718	26	0.48
Cooperation of PDCL (PHLP1) and TRIC CCT in G-protein beta folding	Reactome pathway	0.04785	15	0.54
Response to peptide	GO:1901652	0.04876	84	0.37
Glutamine family amino acid metabolic process	GO:0009064	0.04901	20	0.50

ES, enrichment score. Results are sorted by the nominal P-value in an ascending order.

### Phospho-proteome profile of the left ventricle in HFpEF

**FIGURE 4:**
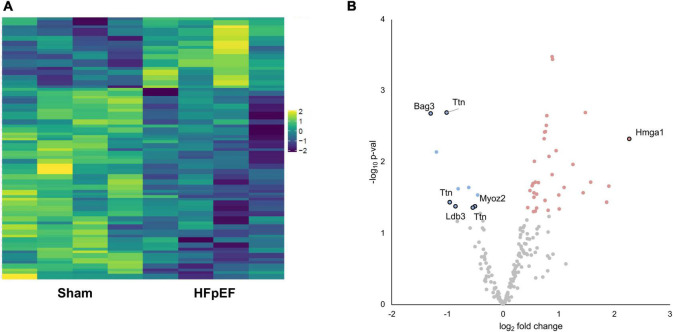
Phosphoproteomics analysis. **(A)** Heatmap of the differentially expressed phosphosites. **(B)** Volcano plot presenting log-transformed *p*-values (*t*-test) associated with individual significantly altered phosphosites plotted against log-transformed fold change in abundance between the left ventricles in Sham and HFpEF mice. Blue and red dots represent up-regulation and down-regulation in HFpEF (*N* = 4) vs. Sham (*N* = 4), respectively.

We next investigated the phosphoproteomics dataset. Phosphoproteomics analysis profiled 281 mouse reference protein sequences, of which 240 mapped to serine, 37 mapped to threonine and 3 mapped to tyrosine residues, consistent with the expected 90:9:1 cellular distribution ratio (22). The abundance of 72 phosphosites was differentially altered (elevated or reduced) between HFpEF and Sham (*P* < 0.05; Figures 4A,B).

Aberrant phosphorylation patterns occurred on proteins linked to disparate subcellular compartments, ranging from sarcomeric proteins (LIM domain-binding protein 3, LDB3; myozenin 2, MYOZ2; titin, TTN), to nuclear-localized proteins (BAG family molecular chaperone regulator 3, BAG3; high mobility group protein HMG-I/HMG-Y, HMGA1) with established links to cardiac contractile function, cardiac hypertrophy and/or cardiomyopathy (Figure 4B and Supplementary Table 2).

### Left ventricular titin expression and phosphorylation in HFpEF

Despite global proteomics not showing a significant change in total titin in the LV between HFpEF and Sham mice, extensive phosphorylation changes across titin were observed in HFpEF vs. Sham. We identified 22 titin phosphosites (including 76% serines, 19% threonines, and 4% tyrosines) in the phospho-dataset, and among them, seven phosphosites were *P* < 0.05, all considered class 1 (localization probability 0.75–1.00) (Table 4). Interestingly, five of these were located at the z disk binding region (S262, S264, T266, S1411, and S1415) while the remainder were residues in the C-terminal region (S34464, T34467), suggesting changes in the mechano-sensing activity of titin. These regions are known to act as titin “hotspots,” which respond to mechanical stress and regulate specific actions such as activating the hypertrophic gene program or interacting with the protein quality control machinery (62, 63).

**TABLE 4:** Significantly changed titin phosphosites.

Feature	Log FC (Sham/HFpEF)	*P* value	Position	Site probability (%)	Peptide sequence
Ttn_A2ASS6.55	−1.01327	0.00203	S262	0.99876	QLPHKTPPRIPPKPKSRSPTPPSIAAKAQLA
Ttn_A2ASS6.56	−1.01327	0.00203	S264	0.99836	PHKTPPRIPPKPKSRSPTPPSIAAKAQLARQ
Ttn_A2ASS6.60	−1.01327	0.00203	T266	0.99033	KTPPRIPPKPKSRSPTPPSIAAKAQLARQQS
Ttn_A2ASS6.36	−0.96294	0.03670	S34464	0.96352	VTSPPRVKSPEPRVKSPETVKSPKRVKSPEP
Ttn_A2ASS6.40	−0.96294	0.03670	T34467	0.81504	PPRVKSPEPRVKSPETVKSPKRVKSPEPVTS
Ttn_A2ASS6.29	−0.54438	0.04350	S1411	0.79632	PTPEAVSRIRSVSPRSLSRSPIRMSPAMSPA
Ttn_A2ASS6.30	−0.54438	0.04350	S1415	0.96095	AVSRIRSVSPRSLSRSPIRMSPAMSPARMSP
Ttn_A2ASS6.27	−0.41168	0.05240	S283	0.89160	PSIAAKAQLARQQSPSPIRHSPSPVRHVRAP
Ttn_A2ASS6.28	−0.41168	0.05240	S290	0.95098	QLARQQSPSPIRHSPSPVRHVRAPTPSPVRS
Ttn_A2ASS6.23	0.42746	0.06498	S34451	0.82907	TLTVQKARVIEKAVTSPPRVKSPEPRVKSPE
Ttn_A2ASS6.24	0.42746	0.06498	S34457	0.98108	ARVIEKAVTSPPRVKSPEPRVKSPETVKSPK
Ttn_A2ASS6.38	0.31502	0.10499	T33859	0.99883	LTQDDLEMVRPARRRTPSPDYDLYYYRRRRR
Ttn_A2ASS6.31	−0.31311	0.19004	S34107	0.99068	DAERRSPTPERTRPRSPSPVSSERSLSRFER
Ttn_A2ASS6.32	−0.33175	0.22516	S34109	0.76541	ERRSPTPERTRPRSPSPVSSERSLSRFERSA
Ttn_A2ASS6.25	1.12758	0.27026	S1406	1.00000	APTYMPTPEAVSRIRSVSPRSLSRSPIRMSP
Ttn_A2ASS6.26	1.12758	0.27026	S1408	1.00000	TYMPTPEAVSRIRSVSPRSLSRSPIRMSPAM
Ttn_A2ASS6.41	0.46224	0.28856	Y33864	0.93320	LEMVRPARRRTPSPDYDLYYYRRRRRSLGDM
Ttn_A2ASS6.33	−0.27602	0.31791	S34112	0.88295	SPTPERTRPRSPSPVSSERSLSRFERSARFD
Ttn_A2ASS6.22	0.19164	0.43120	S307	0.93434	VRHVRAPTPSPVRSVSPAGRISTSPIRSVKS
Ttn_A2ASS6.42	−0.06749	0.79212	S301	0.99417	RHSPSPVRHVRAPTPSPVRSVSPAGRISTSP
Ttn_A2ASS6.43	−0.06749	0.79212	S307	0.93434	VRHVRAPTPSPVRSVSPAGRISTSPIRSVKS
Ttn_A2ASS6.58	−0.06749	0.79212	T299	0.99713	PIRHSPSPVRHVRAPTPSPVRSVSPAGRIST

Results are sorted by the nominal P-value in an ascending order. Highlighted values indicate significantly regulated phosphorylation between Sham and HFpEF.

**FIGURE 5:**
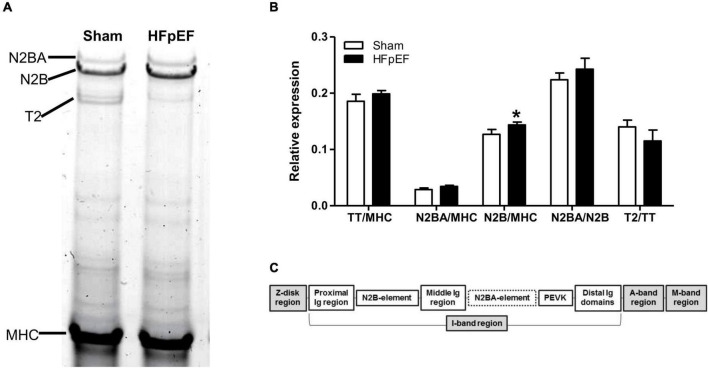
Titin isoform expression. **(A)** Representative image of 1% agarose gel for titin analysis. **(B)** Quantitative analysis of total titin (TT) and titin isoforms N2BA and N2B relative to total myosin heavy chain (MHC), the ratio of N2BA to N2B, and titin degradation product (T2) relative to TT, in the left ventricles from Sham (*N* = 7) and HFpEF mice (*N* = 11). Data are presented as mean ± SEM. Unpaired *T*-test was performed, *P < 0.05 vs. Sham mice. **(C)** Overview of the titin molecule structure.

Additionally, high-resolution gel electrophoresis was performed to further examine other potential switches in titin isoform expression which may affect titin stiffness, i.e., to quantitatively detect changes in the stiffer N2B or the compliant N2BA isoforms of titin (Figure 5A). As expected, there were no changes in total titin (TT) expression between HFpEF and Sham mice. However, as previously described (64), N2B expression was significantly increased in the LV from HFpEF compared to Sham mice (0.144 ± 0.010 vs. 0.127 ± 0.010; *P* < 0.05). Neither N2BA expression, N2BA/N2B ratio nor titin degradation were differentially altered between HFpEF and Sham mice (Figure 5B).

### Left ventricular expression of SIRT3 in HFpEF mice

**FIGURE 6:**
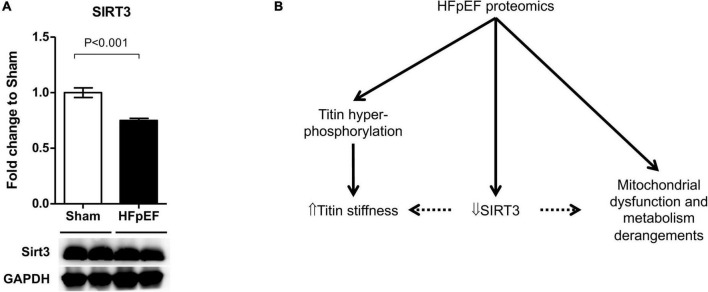
SIRT3 regulation in HFpEF. **(A)** SIRT3 protein expression in the left ventricles from HFpEF mice (*N* = 11) and Sham (*N* = 7). Data are presented as mean ± SEM. Statistical analysis by two-tailed Student’s *t*-test. **(B)** Overview of proposed SIRT3 regulatory system in HFpEF.

Accumulating evidence suggests that SIRT3 plays a critical role in the development of HF (65), particularly in HFpEF (60, 66, 67). As the global proteomics dataset showed a decreased in SIRT3 in the LV from HFpEF mice vs. Sham (*P* = 0.000914), we thus performed additional immunoblot analysis to validate these findings. Indeed, SIRT3 expression was significantly decreased in the LV from HFpEF mice vs. Sham (0.8 ± 0.0 vs. 1.0 ± 0.0; *P* < 0.001; Figure 6A).

## Discussion

Heart failure with preserved ejection fraction is a complex disease involving several sub-phenotypes within a heterogeneous HFpEF syndrome (10, 13, 68). Of all the comorbidities in HFpEF, hypertension remains the most common, and is implicated in both the pathogenesis and the prognosis of the disease (12, 29). However, the exact biological mechanisms that underlie hypertension associated HFpEF remain largely unclear. In this study, we investigated the proteomic and phosphoproteomics profile underlying HFpEF in a clinically relevant murine model of hypertension associated HFpEF. The *SAUNA* model of HFpEF model fulfils the criteria for a “high probability of HFpEF” based on HFA-PEFF diagnostic algorithm for human HFpEF (58, 69).

In the present study, extensive proteomics and phosphoproteomics analysis permitted in-depth screening of the changes in protein expression, post-translational modifications (i.e., phosphorylation), and pathway alterations in HFpEF. These included but were not limited to: (I) changes in cardiac metabolism, where the predominant components were the mitochondrial metabolic processes and mitochondrial dysfunction; (II) alteration in cardiac contractile function-related proteins; (III) overexpression of pathways related to immune modulation; and (IV) a significant decrease in SIRT3 expression, that was validated by immunoblotting.

We found marked changes in signatures of protein expression related to mitochondrial function and oxidative metabolism of energy substrates in HFpEF. There was a significant decrease in targets related to mitochondrial substrate oxidation, suggesting that cardiac mitochondrial metabolic function is impaired in HFpEF. Interestingly, there was an upregulation of the ketone bodies transporter SLC16A1 in the LV of HFpEF, but this was not accompanied by comparable changes in ketone metabolism enzymes. Although not investigated in this study, these findings may contribute to the metabolic impairment seen in HFpEF by increasing the transport of ketone bodies into the mitochondria, but without a compensatory catabolic response. We hypothesize that this mismatch in mitochondrial substrate intake and utilization results in mitochondrial ketone bodies accumulation which may detrimentally affect cardiac function (70). Ketone bodies are thought to be a relevant energy source in both preclinical HFrEF models (71) and advance HFrEF patients (72). Additionally, it has been shown that HFpEF patients have significantly higher circulating ketone levels than HFrEF patients (73) suggesting that some of the beneficial effects of SGLT2 inhibitors in HFpEF may be due to enhanced ketone bodies availability and cardiac utilization (74–76), a process known as “thrifty substrate/fuel hypothesis” (77). We also observed decreased OXCT1 (aka SCOT, succinyl-CoA:3-ketoacid CoA transferase) expression in HFpEF hearts (Figure 3). OXCT1 allows cells to utilize energy stored in ketone bodies thus its decrease in HFpEF hearts supports a role for ketone body cardiac metabolism. Similarly, others have shown worse HF in pre-clinical models with cardio-specific deletion of OXCT1 (78).

Proteomic evaluation of PTMs is essential to understand the function of many proteins in physiological and pathophysiological settings. PTMs are regulators of protein structure and function and, in the heart the predominant PTM is phosphorylation, followed by acetylation (79), and it is also recognized that many proteins are regulated by phosphorylation independently of their expression (80).

Titin is a major cardiac protein regulated by phosphorylation and facilitates myocardial passive tension by conditioning cardiomyocyte-derived stiffness (81). Titin regulates cardiomyocyte stiffness both at the transcriptional and post-transcriptional level. At the transcriptional level, titin shifts from its compliant isoform N2BA toward its stiff isoform N2B, which contributes to the impaired diastolic function that is seen in HFpEF (33, 64, 82). In the present study, translational and PTMs in titin are apparent in the LV of HFpEF hearts. The stiffer N2B isoform was significantly increased in HFpEF mice. However, it is notably that the N2B isoform is also the predominant isoform expressed in the LV of rodents (83). At the post-transcriptional level, despite comparable global proteomics expression between HFpEF mice and Sham, phosphoproteomics analysis showed that titin was one of the proteins with the greatest alterations in phosphorylation in HFpEF mice. Similar to a Dahl salt-sensitive rat study (84), in these *SAUNA* HFpEF mice most of the significantly hyper-phosphorylated titin residues were located at the Z-disk binding region of the titin protein (Figure 5C). Interestingly, it has been suggested that titin may be part of a Z-disk macromolecular machinery acting as a node for hypertrophic signaling (85). As such, our findings that the myofilament and myofilament-associated proteins viz. ACTA1, MYH7, MHY9, TPM1, and MYOZ2 were differentially expressed in both global and phosphoproteomics dataset, support the premise that alterations in sarcomeric and myofilament regulating proteins play a central role in HFpEF. Of these proteins, MYH7, TPM1 and MYOZ2 are known to be important in hypertrophic cardiomyopathy (86–88), and may play a similar role in HFpEF. However, although their function in muscle contraction is well known (89, 90), their role in cardiac hypertrophy and adverse cardiac remodeling remains elusive. It has been hypothesized that changes in cardiac architecture may be a compensatory response that eventually fails, resulting in a re-induction of fetal genes, fibrosis replacing necrotic and apoptotic cardiac cells, and a shift in metabolic substrates (91). However, additional studies are warranted to identify the precise role these myofilament-associated proteins play in HFpEF.

Because of its size, titin has more phosphorylation sites than other smaller proteins and hundreds of phosphorylation sites have been predicted based on proteomic analysis (83), Z-disk Similarly, multiple kinases are also involved in titin phosphorylation (92), representing more opportunities for the regulation of the cardiomyocyte structure and function. However, the effect that a specific phosphorylation pattern has on the function of titin is largely dependent on the specific structural domain which is modified within the protein (93). For example several studies have focused on the “spring-like” I-domain, including the N2bus and PEVK regions, likely due to the mechanically active nature of this specific domain, where phosphorylation may modulate passive and active tension of the sarcomere (85, 92–94). Conversely, the proline-directed kinases, including extracellular signal-regulated kinase-1/-2 (ERK1/2) and cyclin-dependent protein kinase-2 (Cdc2) were able to regulate the phosphorylation status of non-extensible Z-disk (95, 96) and C-terminal (M-band) (97) regions (98). Although additional studies using site-specific methods are needed (92), it has been suggested that changes in the phosphorylation status of these regions may have an important function, not only during developmental stages, but also regulating the binding of titin to other and M-band proteins, as well as the assembly and turnover of these binding partners (99, 100).

In addition to phosphorylation, HFpEF is also associated with hyperacetylation of mitochondrial proteins in the myocardium (101, 102). In the mitochondria, the acetylation state of key enzymes involved in mitochondrial metabolism, oxidative stress defense and mitochondrial dynamics is regulated by the mitochondrial, NAD-dependent protein deacetylase SIRT3 (103–106). SIRT3 interacts with at least 84 mitochondrial proteins involved in many aspects of mitochondrial biology, such as maintaining mitochondrial integrity and function (67, 106). In the present study, the global proteomics data set showed decreased expression of SIRT3 in the LV of HFpEF mice, which was also confirmed by immunoblotting. Others have shown that reduced SIRT3 expression is related to reduced NAD+ bioavailability in HFpEF, and that cardiomyocyte specific SIRT3 knockout mice developed worse diastolic dysfunction in HFpEF (60). Additional studies using whole-body knockout or transgenic mice similarly showed that SIRT3 is required to maintain cardiac contractile function under pro-hypertrophic or ischemic stress (107–110). A recent study showed that a deficit of cardiac NAD+ exists not only pre-clinical HFpEF models but also in patients with HFpEF, and that increasing NAD+ levels with nicotinamide improved diastolic dysfunction (111). The authors hypothesized the beneficial effects were mediated, partly by increasing deacetylation of proteins that regulate the mechano-elastic properties of cardiac myocytes such as titin and sarco/endoplasmic reticulum Ca^2+^ ATPase 2a (SERCA2a). Although not investigated in the present study, SIRT3 may also play a role in cardiomyocyte stiffness and impaired diastolic function in HFpEF, possibly by titin acetylation (59). SIRT 3 may be a future target since, compared to younger subjects, exercise increases SIRT3 protein expression in muscle, which is decreased in older sedentary individuals (112). Interestingly, of the 7 mammalian sirtuins described, SIRT3 is the only analog whose increased expression associates with longevity in humans (113–115). Since HFpEF is highly associated with aging, and exercise training is effective in improving the quality of life in HFpEF patients (116), future studies are warranted to explore the role of SIRT3 expression in muscle in patients with HFpEF.

In conclusion, untargeted proteomics have demonstrated a key role of protein PTMs in metabolism, cell preservation and sarcomere function in the heart (117). In the present study marked proteomics and phosphoproteomics changes occurred in the heart in HFpEF mice which were related to altered mitochondrial metabolism and sarcomere contractility. It is possible that SIRT3 plays a pivotal role in HFpEF, by regulating mitochondrial metabolism and titin stiffness but this requires further study (Figure 6B).

## Data availability statement

The datasets presented in this study can be found online at the PRIDE Archive, proteomics data repository (European Bioinformatics Institute, European Molecular Biology Laboratory) with the data set identifier PXD033501. Raw unedited gels images are shown in Supplementary Figure 3.

## Ethics statement

The animal study was reviewed and approved by the Institutional Animal Care and Use Committee at Boston University School of Medicine.

## Author contributions

MV-M and FS contributed to the conception and design of the study. MV-M and ES performed the surgeries and physiological measurements. MV-M, ES, and ZH performed the molecular analysis. RH and BB performed the proteomic sample preparation and carried out the mass spectrometry and bioinformatics analysis. MV-M, ES, and FS wrote the first draft of the manuscript. RH wrote sections of the manuscript. All authors contributed to the manuscript revision, read, and approved the submitted version.

## Footnotes

1
http://maxquant.org/

2
www.uniprot.org/taxonomy/10090

3
https://baderlab.org/GeneSets

## Abbreviations

ACTA1: skeletal alpha-actin
EF: ejection fraction
GLUT1: glucose transporter 1
GLUT4: glucose transporter 4
HF: heart failure
HFpEF: heart failure with preserved ejection fraction
HFrEF: heart failure with reduced ejection fraction
IVST: interventricular septum wall thickness
LV: left ventricle
LVEF: left ventricle ejection fraction
LVEDD: left ventricle end diastolic diameter
LVESD: left ventricle end systolic diameter
MFN1: mitofusin 1
MS/MS: tandem mass spectrometry
MYH7: beta-myosin heavy chain
MYH9: myosin heavy chain 9
OXCT1: succinyl-CoA:3-ketoacid coenzyme A transferase 1
PM1: tropomyosin alpha-1 chain
PTM: post-translational modification
RWT: relative wall thickness
SLC16A1: monocarboxylate transporter 1
SIRT3: sirtuin-3
TFAM: transcription factor A mitochondrial
TWT: total wall thickness.
