# Supplementary material for: Proteomic and phosphoproteomic profiling in heart failure with preserved ejection fraction (HFpEF)
Source: Front Cardiovasc Med. 2022 Aug 25;9:966968. doi: 10.3389/fcvm.2022.966968 (PMC9452734; doi:10.3389/fcvm.2022.966968)
Supplement: Supplementary file 1 [file Data_Sheet_1.pdf]

Supplementary Material

*Supplementary Material*

## 1 Supplementary Figures and Tables

### 1.1 Supplementary Figures

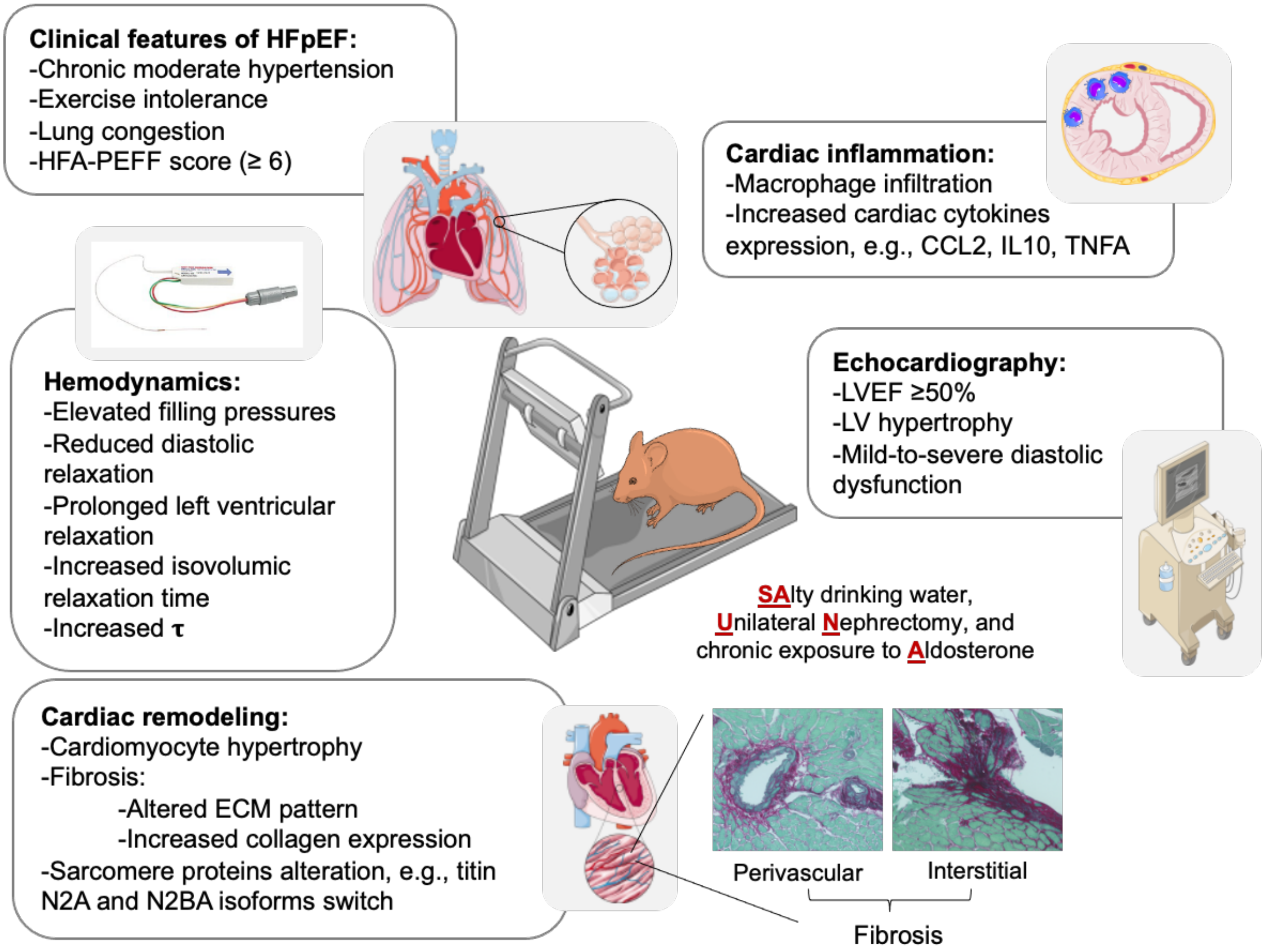


**Supplemental Figure 1: HFpEF (SAUNA) mouse model recapitulates human HFpEF.** The Figure was partly generated using Servier Medical Art, provided by Servier, licensed under a Creative Commons Attribution 3.0 unported license.

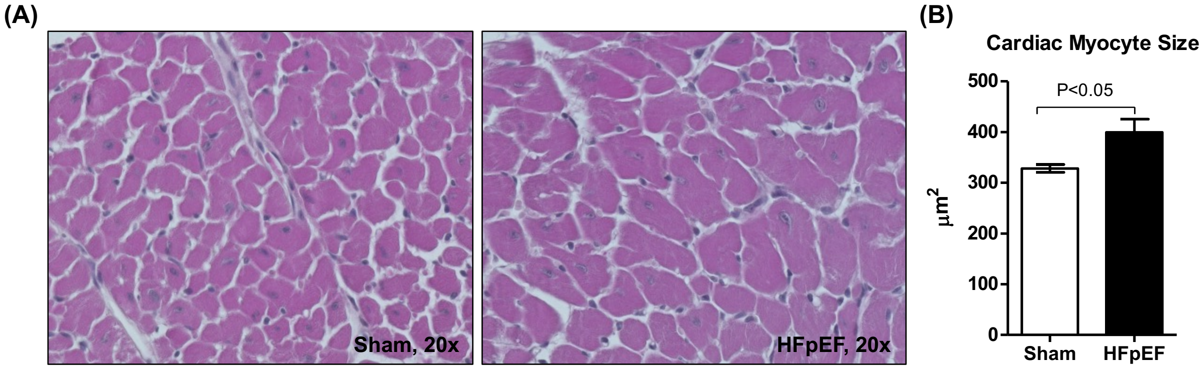


**Supplementary Figure 2. Cardiomyocyte size**. (**A**) Representative image of hematoxilin and eosin staining in the left ventricle from Sham and HFpEF mice hearts. (**B**) Quantitative analysis of cardiac myocyte cross-sectional area from the left ventricle.

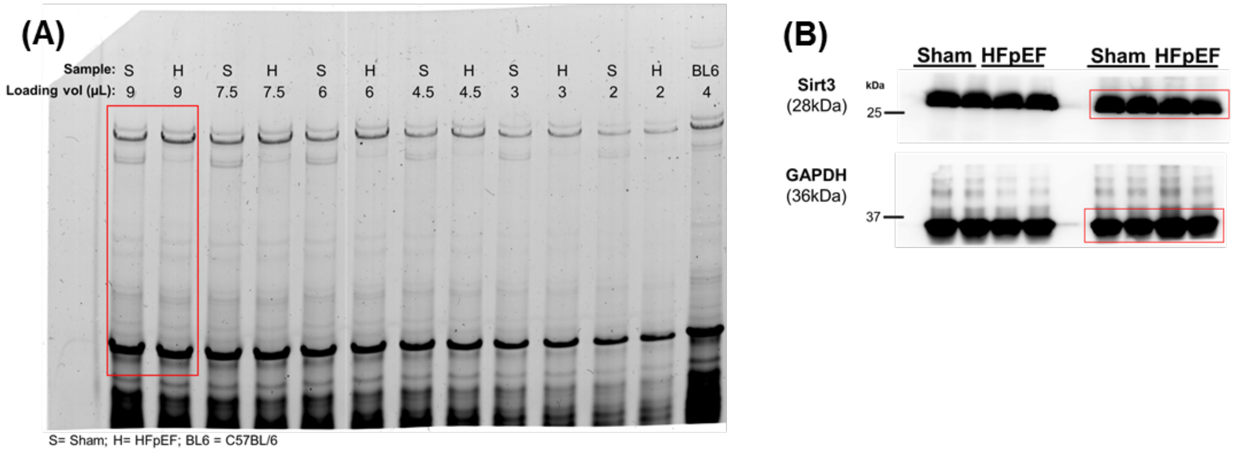


**Supplemental Figure 3: Full unedited gels images.** (**A**) Uncropped blot of Titin isoforms shown in Figure 5A. (**B**) Uncropped blot of SIRT3 shown in Figure 6.

## 1.2 Supplementary Tables

| UNIPROT | PROTEIN | logFC | P.VALUE |
|---|---|---|---|
| P50136 | 2-oxoisovalerate dehydrogenase subunit alpha, mitochondrial | 0.57 | 2.12E-05 |
| Q9QYE6 | Golgin subfamily A member 5 | 1.14 | 2.71E-05 |
| Q80XL6 | Acyl-CoA dehydrogenase family member 11 | 0.66 | 2.76E-05 |
| P68134 | Actin, alpha skeletal muscle | -0.75 | 3.92E-05 |
| Q8R2Y2 | Cell surface glycoprotein MUC18 | 0.56 | 4.99E-05 |
| Q9WUP7 | Ubiquitin carboxyl-terminal hydrolase isozyme L5 | 0.53 | 5.04E-05 |
| Q9CYK1 | Tryptophan--tRNA ligase, mitochondrial | 0.42 | 5.82E-05 |
| Q9ERD7 | Tubulin beta-3 chain | 0.43 | 7.18E-05 |
| Q99L04 | Dehydrogenase/reductase SDR family member 1 | 0.63 | 0.000102 |
| Q9CPU0 | Lactoylglutathione lyase | 0.76 | 0.00011 |
| Q8BX80 | Cytosolic endo-beta-N-acetylglucosaminidase | 0.93 | 0.000123 |
| Q9JKX6 | ADP-sugar pyrophosphatase | 0.53 | 0.000146 |
| Q9QXT0 | Protein canopy homolog 2 | 0.75 | 0.000169 |
| Q8BGD8 | Cytochrome c oxidase assembly factor 6 homolog | 0.40 | 0.000169 |
| Q6PIE5 | Sodium/potassium-transporting ATPase subunit alpha-2 | 0.59 | 0.00017 |
| Q8VBZ3 | Cleft lip and palate transmembrane protein 1 homolog | 0.47 | 0.000207 |
| Q04447 | Creatine kinase B-type | 0.41 | 0.000215 |
| Q9R0E2 | Procollagen-lysine,2-oxoglutarate 5-dioxygenase 1 | 0.61 | 0.000232 |
| A6X935 | Inter alpha-trypsin inhibitor, heavy chain 4 | -0.33 | 0.000248 |
| Q9CPW9 | Methionine aminopeptidase 1D, mitochondrial | 0.97 | 0.000258 |
| Q99K41 | EMILIN-1 | 0.65 | 0.000261 |
| P59672 | Ankyrin repeat and SAM domain-containing protein 1A | 0.78 | 0.000306 |
| Q3UR70 | Transforming growth factor-beta receptor-associated protein 1 | -0.84 | 0.000328 |
| Q61543 | Golgi apparatus protein 1 | 0.30 | 0.000355 |
| P22892 | AP-1 complex subunit gamma-1 | 0.66 | 0.000361 |
| Q9DCU6 | 39S ribosomal protein L4, mitochondrial | 0.36 | 0.000363 |
| Q8BT60 | Copine-3 | 0.47 | 0.000373 |
| Q9WVC3 | Caveolin-2 | 0.35 | 0.000374 |
| P08752 | Guanine nucleotide-binding protein G(i) subunit alpha-2 | 0.30 | 0.000385 |
| Q9D710 | Thioredoxin-related transmembrane protein 2 | 0.61 | 0.000396 |
| Q8VDD5 | Myosin-9 | 0.34 | 0.000408 |
| Q9R1P1 | Proteasome subunit beta type-3 | 0.42 | 0.000421 |
| Q8VC30 | Bifunctional ATP-dependent dihydroxyacetone kinase/FAD-AMP lyase (cyclizing);ATP-dependent dihydroxyacetone kinase;FAD-AMP lyase (cyclizing) | 0.47 | 0.000423 |
| P18872 | Guanine nucleotide-binding protein G(o) subunit alpha | 0.44 | 0.000426 |
| Q8CGA0 | Protein phosphatase 1F | 0.37 | 0.000426 |
| P02802 | Metallothionein-1 | -0.55 | 0.000444 |

| | | | |
|---|---|---|---|
| Q60759 | Glutaryl-CoA dehydrogenase, mitochondrial | 0.33 | 0.000449 |
| Q3TPX4 | Exocyst complex component 5 | 0.42 | 0.000451 |
| Q9CZ42 | ATP-dependent (S)-NAD(P)H-hydrate dehydratase | 0.44 | 0.000479 |
| Q8C9H6 | Striatin-interacting proteins 2 | 0.93 | 0.00048 |
| Q9D708 | S100-A16 | -0.64 | 0.000487 |
| P03903 | NADH-ubiquinone oxidoreductase chain 4L | 0.65 | 0.000489 |
| Q8K4B4 | Interleukin-23 receptor | -1.31 | 0.000523 |
| Q9JK92 | Heat shock protein beta-8 | -0.54 | 0.00054 |
| Q8CFI0 | E3 ubiquitin-protein ligase NEDD4-like | 0.48 | 0.000543 |
| P55284 | Cadherin-5 | 0.47 | 0.000566 |
| Q91YR1 | Twinfilin-1 | 0.44 | 0.000573 |
| Q8CGY6 | Protein unc-45 homolog B | 0.29 | 0.000584 |
| P37889 | Fibulin-2 | -0.51 | 0.00059 |
| Q9D7X3 | Dual specificity protein phosphatase 3 | 0.50 | 0.000593 |
| Q923B6 | Metalloreductase STEAP4 | 0.86 | 0.000621 |
| Q8BM72 | Heat shock 70 kDa protein 13 | 0.40 | 0.000623 |
| P52293 | Importin subunit alpha-1 | 0.35 | 0.000632 |
| Q6ZWM4 | U6 snRNA-associated Sm-like protein LSm8 | 0.34 | 0.000662 |
| P63323 | 40S ribosomal protein S12 | 0.59 | 0.00067 |
| Q99M04 | Lipoyl synthase, mitochondrial | 0.42 | 0.000683 |
| O88935 | Synapsin-1 | 2.32 | 0.000683 |
| Q8CIH5 | 1-phosphatidylinositol 4,5-bisphosphate phosphodiesterase gamma-2 | 0.86 | 0.000688 |
| Q9D1F4 | Proline-rich AKT1 substrate 1 | 0.54 | 0.000699 |
| Q9CQN3 | Mitochondrial import receptor subunit TOM6 homolog | 0.33 | 0.000708 |
| Q3UGC7 | Eukaryotic translation initiation factor 3 subunit J-A;Eukaryotic translation initiation factor 3 subunit J-B | 0.36 | 0.000718 |
| Q8CFE4 | SCY1-like protein 2 | 0.50 | 0.000802 |
| Q8R1I1 | Cytochrome b-c1 complex subunit 9 | 0.46 | 0.00082 |
| P15105 | Glutamine synthetase | 0.43 | 0.00082 |
| Q61738 | Integrin alpha-7;Integrin alpha-7 heavy chain;Integrin alpha-7 light chain | 0.46 | 0.000847 |
| P12367 | cAMP-dependent protein kinase type II-alpha regulatory subunit | 0.29 | 0.000857 |
| Q9CX34 | Suppressor of G2 allele of SKP1 homolog | 0.61 | 0.000868 |
| P21619 | Lamin-B2 | 0.37 | 0.000888 |
| Q61553 | Fascin | 0.65 | 0.000893 |
| Q8R104 | NAD-dependent protein deacetylase sirtuin-3 | 0.40 | 0.000915 |
| Q9DBB9 | Carboxypeptidase N subunit 2 | 0.59 | 0.000922 |
| O70252 | Heme oxygenase 2 | 0.30 | 0.000923 |
| Q9D0M5 | Dynein light chain 2, cytoplasmic | -0.82 | 0.000928 |
| Q8BFZ3 | Beta-actin-like protein 2 | 0.30 | 0.000935 |
| Q9CZD5 | Translation initiation factor IF-3, mitochondrial | 0.41 | 0.000938 |

| | | | |
|---|---|---|---|
| Q9D1A2 | Cytosolic non-specific dipeptidase | 0.33 | 0.000942 |
| Q64010 | Adapter molecule crk | 0.42 | 0.000966 |
| Q99MD6 | Thioredoxin reductase 3 | 0.56 | 0.000972 |
| Q99J47 | Dehydrogenase/reductase SDR family member 7B | 0.32 | 0.000975 |
| Q8K009 | Mitochondrial 10-formyltetrahydrofolate dehydrogenase | 0.31 | 0.00098 |
| D3YZP9 | Coiled-coil domain-containing protein 6 | 0.50 | 0.000982 |
| Q9JK48 | Endophilin-B1 | -0.63 | 0.001006 |
| Q3TFD2 | Lysophosphatidylcholine acyltransferase 1 | 0.67 | 0.001047 |
| O88844 | Isocitrate dehydrogenase [NADP] cytoplasmic | 0.44 | 0.001047 |
| Q3UMU9 | Hepatoma-derived growth factor-related protein 2 | -0.39 | 0.001057 |
| Q811U4 | Mitofusin-1 | 0.35 | 0.001059 |
| P17918 | Proliferating cell nuclear antigen | 0.40 | 0.001124 |
| Q8BTR5 | Dual specificity phosphatase 28 | 0.41 | 0.001128 |
| Q0VG49 | Uncharacterized protein C15orf61 homolog | 0.74 | 0.001129 |
| Q9CQA9 | Cancer-related nucleoside-triphosphatase homolog | 0.48 | 0.00114 |
| Q6PEB4 | Probable tRNA N6-adenosine threonylcarbamoyltransferase, mitochondrial | 0.31 | 0.001179 |
| P05977 | Myosin light chain 1/3, skeletal muscle isoform | 0.26 | 0.001206 |
| Q3TEA8 | Heterochromatin protein 1-binding protein 3 | 0.35 | 0.001218 |
| Q8VDQ1 | Prostaglandin reductase 2 | 0.30 | 0.001257 |
| Q9D8Y0 | EF-hand domain-containing protein D2 | 0.52 | 0.001262 |
| P30416 | Peptidyl-prolyl cis-trans isomerase FKBP4;Peptidyl-prolyl cis-trans isomerase FKBP4, N-terminally processed | 0.26 | 0.001272 |
| P30681 | High mobility group protein B2 | -0.61 | 0.001275 |
| Q9Z1J3 | Cysteine desulfurase, mitochondrial | 0.47 | 0.001279 |
| Q9QUH0 | Glutaredoxin-1 | 0.34 | 0.001305 |
| Q9JKL5 | Calcineurin B homologous protein 3 | 0.41 | 0.001305 |
| Q9QXB9 | Developmentally-regulated GTP-binding protein 2 | 0.57 | 0.001314 |
| G5E897 | O-glucosyltransferase 3 | 0.97 | 0.001323 |
| Q8BIJ6 | Isoleucine--tRNA ligase, mitochondrial | 0.27 | 0.00133 |
| Q6ZWY9 | Histone H2B type 1-C/E/G;Histone H2B type 1-H;Histone H2B type 1-F/J/L;Histone H2B type 1-K | 0.38 | 0.001341 |
| P58404 | Striatin-4 | 0.55 | 0.001351 |
| Q9CQR6 | Serine/threonine-protein phosphatase 6 catalytic subunit;Serine/threonine-protein phosphatase 6 catalytic subunit, N-terminally processed | 0.43 | 0.001373 |
| Q6NT99 | Dual specificity protein phosphatase 23 | -0.40 | 0.001378 |
| O08807 | Peroxiredoxin-4 | 0.31 | 0.001402 |
| O88967 | ATP-dependent zinc metalloprotease YME1L1 | 0.57 | 0.001407 |
| Q9D8Y1 | Transmembrane protein 126A | 0.32 | 0.001417 |
| P55258 | Ras-related protein Rab-8A | 0.32 | 0.001421 |
| O54692 | Centromere/kinetochore protein zw10 homolog | 0.63 | 0.001423 |

| | | | |
|---|---|---|---|
| Q641P0 | Actin-related protein 3B | 0.45 | 0.001431 |
| Q9QYS9 | Protein quaking | 0.56 | 0.001463 |
| Q9D9V3 | Ethylmalonyl-CoA decarboxylase | 0.52 | 0.001472 |
| E9Q4Z2 | Acetyl-CoA carboxylase 2;Biotin carboxylase | 0.25 | 0.001474 |
| Q8R105 | Vacuolar protein sorting-associated protein 37C | 0.31 | 0.001482 |
| P97449 | Aminopeptidase N | 0.61 | 0.001485 |
| Q8K297 | Procollagen galactosyltransferase 1 | 0.53 | 0.001512 |
| O08532 | Voltage-dependent calcium channel subunit alpha-2/delta-1;Voltage-dependent calcium channel subunit alpha-2-1;Voltage-dependent calcium channel subunit delta-1 | 0.28 | 0.001521 |
| P30275 | Creatine kinase U-type, mitochondrial | 0.60 | 0.001533 |
| P54729 | NEDD8 ultimate buster 1 | 0.45 | 0.001537 |
| P38647 | Stress-70 protein, mitochondrial | 0.28 | 0.001553 |
| Q9DBJ1 | Phosphoglycerate mutase 1 | 0.25 | 0.001575 |
| Q8CC88 | von Willebrand factor A domain-containing protein 8 | 0.26 | 0.00158 |
| P61963 | DDB1- and CUL4-associated factor 7 | 0.76 | 0.001634 |
| Q3UHD6 | Sorting nexin-27 | 0.25 | 0.001671 |
| P62874 | Guanine nucleotide-binding protein G(I)/G(S)/G(T) subunit beta-1 | 0.32 | 0.00168 |
| Q8VDN2 | Sodium/potassium-transporting ATPase subunit alpha-1 | 0.23 | 0.001727 |
| Q9Z0P5 | Twinfilin-2 | 0.45 | 0.001727 |
| Q8C0Z1 | Protein ITFG3 | 0.40 | 0.001754 |
| Q9JK23 | Proteasome assembly chaperone 1 | 0.75 | 0.001785 |
| Q922M3 | BTB/POZ domain-containing adapter for CUL3-mediated RhoA degradation protein 3 | -0.83 | 0.001811 |
| Q8K2M0 | 39S ribosomal protein L38, mitochondrial | 0.38 | 0.001818 |
| Q3THS6 | S-adenosylmethionine synthase isoform type-2 | 0.35 | 0.001842 |
| P50516 | V-type proton ATPase catalytic subunit A | 0.35 | 0.00185 |
| Q91XD6 | Vacuolar protein-sorting-associated protein 36 | 0.27 | 0.001872 |
| Q9WUL7 | ADP-ribosylation factor-like protein 3 | 0.32 | 0.001874 |
| Q8CBW3 | Abl interactor 1 | 0.42 | 0.001886 |
| Q64514 | Tripeptidyl-peptidase 2 | 0.27 | 0.001926 |
| Q8R1S0 | Ubiquinone biosynthesis monooxygenase COQ6, mitochondrial | 0.27 | 0.001952 |
| Q8VE65 | Transcription initiation factor TFIID subunit 12 | 1.36 | 0.001987 |
| P63330 | Serine/threonine-protein phosphatase 2A catalytic subunit alpha isoform;Serine/threonine-protein phosphatase 2A catalytic subunit beta isoform | 0.51 | 0.00202 |
| P62852 | 40S ribosomal protein S25 | 0.26 | 0.002023 |
| P06801 | NADP-dependent malic enzyme | 0.23 | 0.002054 |
| Q05920 | Pyruvate carboxylase, mitochondrial | 0.27 | 0.00206 |
| Q68FL6 | Methionine--tRNA ligase, cytoplasmic | 0.35 | 0.002098 |
| Q8K4Z5 | Splicing factor 3A subunit 1 | -0.36 | 0.002098 |

| | | | |
|---|---|---|---|
| Q9JKL4 | NADH dehydrogenase [ubiquinone] 1 alpha subcomplex assembly factor 3 | 0.36 | 0.002197 |
| Q99LH2 | Phosphatidylserine synthase 1 | 0.45 | 0.00221 |
| Q8K2K6 | Arf-GAP domain and FG repeat-containing protein 1 | 0.31 | 0.002224 |
| P27661 | Histone H2AX | -0.57 | 0.002251 |
| Q8K310 | Matrin-3 | 0.21 | 0.002283 |
| P60710 | Actin, cytoplasmic 1;Actin, cytoplasmic 1, N-terminally processed | 0.24 | 0.002303 |
| Q8CHT0 | Delta-1-pyrroline-5-carboxylate dehydrogenase, mitochondrial | 0.32 | 0.002305 |
| Q7TNG5 | Echinoderm microtubule-associated protein-like 2 | 0.36 | 0.002348 |
| Q99N57 | RAF proto-oncogene serine/threonine-protein kinase | 0.54 | 0.002405 |
| Q9CR51 | V-type proton ATPase subunit G 1 | 0.28 | 0.002527 |
| P62242 | 40S ribosomal protein S8 | 0.26 | 0.002531 |
| Q8JZL3 | Thiamine-triphosphatase | 0.25 | 0.002561 |
| Q6PIC6 | Sodium/potassium-transporting ATPase subunit alpha-3 | 0.91 | 0.002615 |
| A2AVZ9 | Solute carrier family 43 member 3 | 0.46 | 0.00262 |
| Q6PD31 | Trafficking kinesin-binding protein 1 | 0.25 | 0.002709 |
| Q99J39 | Malonyl-CoA decarboxylase, mitochondrial | 0.25 | 0.002733 |
| Q9CZD3 | Glycine--tRNA ligase | 0.26 | 0.002741 |
| Q6IRU5 | Clathrin light chain B | 0.39 | 0.002789 |
| P42225 | Signal transducer and activator of transcription 1 | 0.66 | 0.002805 |
| Q9DB77 | Cytochrome b-c1 complex subunit 2, mitochondrial | 0.23 | 0.00281 |
| Q6P1B1 | Xaa-Pro aminopeptidase 1 | 0.42 | 0.002886 |
| Q8K010 | 5-oxoprolinase | 0.48 | 0.002893 |
| Q60590 | Alpha-1-acid glycoprotein 1;Alpha-1-acid glycoprotein 2 | -0.37 | 0.002911 |
| P01898 | H-2 class I histocompatibility antigen, Q10 alpha chain | -0.43 | 0.002917 |
| Q923X4 | Glutaredoxin-2, mitochondrial | 0.34 | 0.002921 |
| P58871 | 182 kDa tankyrase-1-binding protein | 0.36 | 0.002926 |
| Q7TNG8 | Probable D-lactate dehydrogenase, mitochondrial | 0.44 | 0.0031 |
| P10493 | Nidogen-1 | 0.23 | 0.003117 |
| Q9DBD0 | Inhibitor of carbonic anhydrase | 0.54 | 0.003121 |
| Q9Z126 | Platelet factor 4 | 0.32 | 0.003146 |
| Q3THK7 | GMP synthase [glutamine-hydrolyzing] | 0.25 | 0.003149 |
| Q99MI1 | ELKS/Rab6-interacting/CAST family member 1 | 0.24 | 0.003155 |
| Q9D1E6 | Tubulin-folding cofactor B | -0.41 | 0.003162 |
| P56480 | ATP synthase subunit beta, mitochondrial | 0.21 | 0.003166 |
| Q9JLR9 | HIG1 domain family member 1A, mitochondrial | 0.60 | 0.00317 |
| P62254 | Ubiquitin-conjugating enzyme E2 G1;Ubiquitin-conjugating enzyme E2 G1, N-terminally processed | 0.46 | 0.003183 |
| O70622 | Reticulon-2 | 0.75 | 0.003201 |
| Q768S4 | Rab effector Noc2 | -0.96 | 0.00321 |

| | | | |
|---|---|---|---|
| Q02788 | Collagen alpha-2(VI) chain | 0.26 | 0.003239 |
| P26039 | Talin-1 | 0.22 | 0.003254 |
| O08715 | A-kinase anchor protein 1, mitochondrial | 0.57 | 0.003277 |
| Q925I1 | ATPase family AAA domain-containing protein 3 | 0.31 | 0.003284 |
| Q9D824 | Pre-mRNA 3-end-processing factor FIP1 | 0.59 | 0.003286 |
| Q9D7N3 | 28S ribosomal protein S9, mitochondrial | 0.23 | 0.003335 |
| Q8CHP8 | Phosphoglycolate phosphatase | 0.22 | 0.00334 |
| Q8BH51 | Cytochrome c oxidase assembly protein COX14 | -0.53 | 0.003349 |
| Q921L3 | Transmembrane and coiled-coil domain-containing protein 1 | 0.34 | 0.003365 |
| Q8R5A0 | N-lysine methyltransferase SMYD2 | 0.38 | 0.003426 |
| P14148 | 60S ribosomal protein L7 | 0.38 | 0.003521 |
| P46061 | Ran GTPase-activating protein 1 | 0.74 | 0.003545 |
| O70305 | Ataxin-2 | 0.62 | 0.003576 |
| Q9CQW9 | Interferon-induced transmembrane protein 3 | 0.51 | 0.003592 |
| Q9CS72 | Filamin-A-interacting protein 1 | -0.44 | 0.003616 |
| Q3UMT1 | Protein phosphatase 1 regulatory subunit 12C | 0.29 | 0.003641 |
| Q9ER72 | Cysteine--tRNA ligase, cytoplasmic | 0.27 | 0.003646 |
| P48758 | Carbonyl reductase [NADPH] 1 | 0.19 | 0.003649 |
| A2A8Z1 | Oxysterol-binding protein-related protein 9 | 0.25 | 0.003713 |
| P17183 | Gamma-enolase | 0.75 | 0.003735 |
| P53986 | Monocarboxylate transporter 1 | -0.52 | 0.003761 |
| Q791T5 | Mitochondrial carrier homolog 1 | 0.33 | 0.00378 |
| P34914 | Bifunctional epoxide hydrolase 2;Cytosolic epoxide hydrolase 2;Lipid-phosphate phosphatase | 0.31 | 0.003787 |
| Q91YS8 | Calcium/calmodulin-dependent protein kinase type 1 | 0.79 | 0.003822 |
| Q9D3P8 | Plasminogen receptor (KT) | 0.23 | 0.003831 |
| Q64338 | Calcium/calmodulin-dependent 3,5-cyclic nucleotide phosphodiesterase 1C | 0.34 | 0.003912 |
| Q9CWB7 | Glutaredoxin-like protein C5orf63 homolog | 0.42 | 0.003952 |
| Q9D1X0 | Nucleolar protein 3 | 0.32 | 0.003977 |
| P19258 | Protein Mpv17 | 0.32 | 0.004004 |
| Q9D4H8 | Cullin-2 | 0.30 | 0.004064 |
| O09167 | 60S ribosomal protein L21 | -0.42 | 0.004124 |
| Q9R112 | Sulfide:quinone oxidoreductase, mitochondrial | 0.34 | 0.004128 |
| Q80VJ2 | Steroid receptor RNA activator 1 | 0.49 | 0.00413 |
| Q8QZR5 | Alanine aminotransferase 1 | 0.39 | 0.004152 |
| P62264 | 40S ribosomal protein S14 | 0.21 | 0.004225 |
| O70456 | 14-3-3 protein sigma | -0.32 | 0.004228 |
| P97493 | Thioredoxin, mitochondrial | 0.29 | 0.004255 |
| Q9JL35 | High mobility group nucleosome-binding domain-containing protein 5 | 0.51 | 0.004269 |
| Q3V384 | Lactation elevated protein 1 | 0.18 | 0.004319 |

| | | | |
|---|---|---|---|
| Q99LE6 | ATP-binding cassette sub-family F member 2 | 0.49 | 0.004323 |
| Q9CR13 | UPF0562 protein C7orf55 homolog | 0.22 | 0.004438 |
| Q9R069 | Basal cell adhesion molecule | 0.51 | 0.004441 |
| Q62130 | Tyrosine-protein phosphatase non-receptor type 14 | 0.64 | 0.004447 |
| Q9Z1R4 | Uncharacterized protein C6orf47 homolog | -0.64 | 0.004451 |
| Q9WTQ5 | A-kinase anchor protein 12 | -0.25 | 0.004453 |
| P47753 | F-actin-capping protein subunit alpha-1 | 0.24 | 0.00447 |
| Q9DBL9 | 1-acylglycerol-3-phosphate O-acyltransferase ABHD5 | 0.46 | 0.00447 |
| Q9D8S4 | Oligoribonuclease, mitochondrial | 0.28 | 0.00448 |
| O54887 | Testis-specific serine kinase substrate | -0.46 | 0.004485 |
| P51174 | Long-chain specific acyl-CoA dehydrogenase, mitochondrial | 0.19 | 0.00449 |
| Q78ZA7 | Nucleosome assembly protein 1-like 4 | 0.21 | 0.004507 |
| Q7TSQ8 | Pyruvate dehydrogenase phosphatase regulatory subunit, mitochondrial | 0.23 | 0.004523 |
| B9EJ86 | Oxysterol-binding protein | 0.50 | 0.004528 |
| Q9ET26 | E3 ubiquitin-protein ligase RNF114 | -0.48 | 0.004551 |
| Q91WJ8 | Far upstream element-binding protein 1 | -0.29 | 0.004562 |
| O88623 | Ubiquitin carboxyl-terminal hydrolase 2 | 0.39 | 0.004598 |
| Q03734 | Serine protease inhibitor A3M | 0.43 | 0.004608 |
| Q80VK6 | Rho guanine nucleotide exchange factor 38 | -0.74 | 0.004649 |
| Q9Z1R2 | Large proline-rich protein BAG6 | 0.27 | 0.004687 |
| Q6NVE9 | Protein phosphatase PTC7 homolog | 0.25 | 0.004819 |
| Q8R016 | Bleomycin hydrolase | 0.35 | 0.00486 |
| P42125 | Enoyl-CoA delta isomerase 1, mitochondrial | 0.23 | 0.004911 |
| Q9CZS1 | Aldehyde dehydrogenase X, mitochondrial | 0.26 | 0.004954 |
| Q9DBG9 | Tax1-binding protein 3 | 0.51 | 0.004962 |
| Q9CQL1 | Protein mago nashi homolog 2;Protein mago nashi homolog | -0.21 | 0.004978 |
| Q9CPR4 | 60S ribosomal protein L17 | -0.51 | 0.00498 |
| Q8R0Y8 | Mitochondrial coenzyme A transporter SLC25A42 | 0.20 | 0.005012 |
| Q8K2T1 | NmrA-like family domain-containing protein 1 | 0.55 | 0.005057 |
| P07724 | Serum albumin | -0.24 | 0.005094 |
| Q60973 | Histone-binding protein RBBP7;Histone-binding protein RBBP4 | 0.28 | 0.005218 |
| Q60714 | Long-chain fatty acid transport protein 1 | 0.25 | 0.005227 |
| Q61205 | Platelet-activating factor acetylhydrolase IB subunit gamma | 0.23 | 0.005394 |
| Q9CQ91 | NADH dehydrogenase [ubiquinone] 1 alpha subcomplex subunit 3 | 0.56 | 0.005395 |
| Q9CY73 | 39S ribosomal protein L44, mitochondrial | -0.47 | 0.005399 |
| Q6PDH0 | Pleckstrin homology-like domain family B member 1 | 0.23 | 0.005438 |
| P70414 | Sodium/calcium exchanger 1 | -0.24 | 0.005443 |

| | | | |
|---|---|---|---|
| Q9D3D9 | ATP synthase subunit delta, mitochondrial | 0.33 | 0.005467 |
| Q9D892 | Inosine triphosphate pyrophosphatase | 0.60 | 0.005503 |
| Q01279 | Epidermal growth factor receptor | 0.41 | 0.005561 |
| Q60936 | Atypical kinase ADCK3, mitochondrial | 0.22 | 0.005591 |
| Q9WTP7 | GTP:AMP phosphotransferase AK3, mitochondrial | 0.36 | 0.00564 |
| Q61599 | Rho GDP-dissociation inhibitor 2 | -0.41 | 0.005702 |
| P56395 | Cytochrome b5 | 0.33 | 0.005726 |
| Q91XF0 | Pyridoxine-5-phosphate oxidase | 0.47 | 0.005796 |
| Q61234 | Alpha-1-syntrophin | 0.18 | 0.005809 |
| O35127 | Protein C10 | 0.24 | 0.005833 |
| P40630 | Transcription factor A, mitochondrial | 0.23 | 0.005837 |
| Q9D5V5 | Cullin-5 | -0.45 | 0.005895 |
| Q99JW2 | Aminoacylase-1 | 0.50 | 0.005927 |
| P06909 | Complement factor H | -0.30 | 0.005944 |
| Q8VCM5 | Mitochondrial ubiquitin ligase activator of NFKB 1 | 0.28 | 0.005984 |
| P35283 | Ras-related protein Rab-12 | 0.33 | 0.006074 |
| Q61739 | Integrin alpha-6;Integrin alpha-6 heavy chain;Integrin alpha-6 light chain | 0.22 | 0.006077 |
| Q8BWT1 | 3-ketoacyl-CoA thiolase, mitochondrial | 0.22 | 0.006087 |
| Q8K0U4 | Heat shock 70 kDa protein 12A | 0.22 | 0.006122 |
| Q9CZX8 | 40S ribosomal protein S19 | 0.17 | 0.006205 |
| Q9CZ28 | Vacuolar-sorting protein SNF8 | 0.27 | 0.006219 |
| P62245 | 40S ribosomal protein S15a | -0.46 | 0.006325 |
| Q8BHE8 | Uncharacterized protein C2orf47 homolog, mitochondrial | -0.31 | 0.006339 |
| P57759 | Endoplasmic reticulum resident protein 29 | -0.34 | 0.006413 |
| Q8VDP4 | Cell cycle and apoptosis regulator protein 2 | 0.31 | 0.006435 |
| P51910 | Apolipoprotein D | 0.29 | 0.006455 |
| Q99KI0 | Aconitate hydratase, mitochondrial | 0.20 | 0.006456 |
| P19253 | 60S ribosomal protein L13a | 0.38 | 0.006472 |
| P68372 | Tubulin beta-4B chain;Tubulin beta-4A chain | 0.24 | 0.006512 |
| Q9Z0Y1 | Dynactin subunit 3 | -0.30 | 0.006531 |
| P50431 | Serine hydroxymethyltransferase, cytosolic | 0.26 | 0.006543 |
| Q61598 | Rab GDP dissociation inhibitor beta | 0.26 | 0.006547 |
| O88343 | Electrogenic sodium bicarbonate cotransporter 1 | 0.55 | 0.006571 |
| O35286 | Pre-mRNA-splicing factor ATP-dependent RNA helicase DHX15 | 0.43 | 0.006573 |
| P51125 | Calpastatin | -0.47 | 0.006594 |
| Q9CWZ7 | Gamma-soluble NSF attachment protein | 0.29 | 0.0066 |
| Q8CC27 | Voltage-dependent L-type calcium channel subunit beta-2 | 0.55 | 0.006645 |
| Q8CEI1 | BolA-like protein 3 | 0.32 | 0.006652 |
| P55292 | Desmocollin-2 | -0.37 | 0.006676 |

| | | | |
|---|---|---|---|
| P62814 | V-type proton ATPase subunit B, brain isoform | 0.24 | 0.006707 |
| Q9EPL8 | Importin-7 | 0.44 | 0.006754 |
| O08915 | AH receptor-interacting protein | 0.30 | 0.006796 |
| Q9Z0E6 | Interferon-induced guanylate-binding protein 2 | -0.41 | 0.006813 |
| P39061 | Collagen alpha-1(XVIII) chain;Endostatin | 0.19 | 0.006837 |
| Q6PIU9 | Uncharacterized protein FLJ45252 homolog | 0.36 | 0.006841 |
| Q8VHN8 | Protein syndesmos | 0.49 | 0.006906 |
| Q5U458 | DnaJ homolog subfamily C member 11 | 0.20 | 0.006912 |
| O55137 | Acyl-coenzyme A thioesterase 1 | 0.40 | 0.006945 |
| Q91Z83 | Myosin-7 | -0.29 | 0.006963 |
| Q9WVR4 | Fragile X mental retardation syndrome-related protein 2 | 0.51 | 0.007059 |
| P50543 | Protein S100-A11 | -0.63 | 0.007114 |
| P21447 | Multidrug resistance protein 1A;Multidrug resistance protein 1B | 0.24 | 0.007116 |
| Q9WTP6 | Adenylate kinase 2, mitochondrial;Adenylate kinase 2, mitochondrial, N-terminally processed | 0.21 | 0.007134 |
| Q8VDC0 | Probable leucine--tRNA ligase, mitochondrial | 0.26 | 0.007171 |
| Q61838 | Alpha-2-macroglobulin;Alpha-2-macroglobulin 165 kDa subunit;Alpha-2-macroglobulin 35 kDa subunit | -0.22 | 0.007186 |
| Q8C5Q4 | G-rich sequence factor 1 | 0.56 | 0.007187 |
| Q9QZF2 | Glypican-1;Secreted glypican-1 | 0.20 | 0.00724 |
| Q60675 | Laminin subunit alpha-2 | 0.21 | 0.007265 |
| Q99JY0 | Trifunctional enzyme subunit beta, mitochondrial;3-ketoacyl-CoA thiolase | 0.19 | 0.007288 |
| Q60668 | Heterogeneous nuclear ribonucleoprotein D0 | 0.29 | 0.007336 |
| Q9R0P3 | S-formylglutathione hydrolase | 0.18 | 0.007388 |
| P60202 | Myelin proteolipid protein | 0.91 | 0.007447 |
| Q9DBG6 | Dolichyl-diphosphooligosaccharide--protein glycosyltransferase subunit 2 | 0.31 | 0.007525 |
| P47758 | Signal recognition particle receptor subunit beta | 0.27 | 0.007577 |
| Q8K0D5 | Elongation factor G, mitochondrial | 0.22 | 0.007608 |
| Q5EG47 | 5-AMP-activated protein kinase catalytic subunit alpha-1 | 0.31 | 0.007697 |
| P49722 | Proteasome subunit alpha type-2 | 0.26 | 0.007708 |
| Q6IR34 | G-protein-signaling modulator 1 | 0.72 | 0.007759 |
| Q3UKJ7 | WD40 repeat-containing protein SMU1;WD40 repeat-containing protein SMU1, N-terminally processed | 0.52 | 0.007781 |
| Q00623 | Apolipoprotein A-I;Proapolipoprotein A-I;Truncated apolipoprotein A-I | -0.42 | 0.00783 |
| Q9WTR5 | Cadherin-13 | 0.19 | 0.007884 |
| Q8K4Q0 | Regulatory-associated protein of mTOR | 0.33 | 0.007885 |
| Q9CR24 | Nucleoside diphosphate-linked moiety X motif 8, mitochondrial | 0.22 | 0.007938 |
| Q9DCW4 | Electron transfer flavoprotein subunit beta | 0.34 | 0.007969 |

| | | | |
|---|---|---|---|
| Q91WC0 | Histone-lysine N-methyltransferase setd3 | 0.28 | 0.007972 |
| Q9Z1Q5 | Chloride intracellular channel protein 1 | 0.19 | 0.007981 |
| P27601 | Guanine nucleotide-binding protein subunit alpha-13 | 0.51 | 0.008048 |
| Q9CZU6 | Citrate synthase, mitochondrial | 0.30 | 0.008068 |
| O55042 | Alpha-synuclein;Beta-synuclein | 0.22 | 0.008092 |
| Q8BJZ4 | 28S ribosomal protein S35, mitochondrial | 0.19 | 0.008137 |
| Q8BTM8 | Filamin-A | 0.23 | 0.00816 |
| Q61001 | Laminin subunit alpha-5 | 0.25 | 0.008205 |
| P98203 | Armadillo repeat protein deleted in velo-cardio-facial syndrome homolog | 0.47 | 0.008216 |
| Q924D0 | Reticulon-4-interacting protein 1, mitochondrial | 0.21 | 0.008231 |
| P22599 | Alpha-1-antitrypsin 1-2 | -0.29 | 0.008251 |
| Q3UHJ0 | AP2-associated protein kinase 1 | 0.24 | 0.0083 |
| Q9D1H7 | Golgi to ER traffic protein 4 homolog | 0.41 | 0.00838 |
| P67871 | Casein kinase II subunit beta | 0.64 | 0.008387 |
| Q9D7N9 | Adipocyte plasma membrane-associated protein | 0.30 | 0.008406 |
| P46735 | Unconventional myosin-Ib | 0.69 | 0.00841 |
| Q6PGB6 | N-alpha-acetyltransferase 50 | -0.73 | 0.008481 |
| P41216 | Long-chain-fatty-acid--CoA ligase 1 | 0.21 | 0.008561 |
| Q99KJ8 | Dynactin subunit 2 | 0.19 | 0.008604 |
| Q60676 | Serine/threonine-protein phosphatase 5 | 0.38 | 0.00865 |
| Q6P549 | Phosphatidylinositol 3,4,5-trisphosphate 5-phosphatase 2 | 0.28 | 0.008658 |
| Q9CQX8 | 28S ribosomal protein S36, mitochondrial | 0.18 | 0.008681 |
| Q6PD03 | Serine/threonine-protein phosphatase 2A 56 kDa regulatory subunit alpha isoform | 0.49 | 0.008794 |
| Q8BPM0 | Disheveled-associated activator of morphogenesis 1 | 0.42 | 0.008809 |
| Q9JLZ3 | Methylglutaconyl-CoA hydratase, mitochondrial | 0.29 | 0.008903 |
| Q3UM45 | Protein phosphatase 1 regulatory subunit 7 | 0.19 | 0.008914 |
| O08583 | THO complex subunit 4;Aly/REF export factor 2 | 0.23 | 0.00895 |
| Q3TCH7 | Cullin-4A | 0.17 | 0.00895 |
| Q07417 | Short-chain specific acyl-CoA dehydrogenase, mitochondrial | 0.17 | 0.008964 |
| Q9CSN1 | SNW domain-containing protein 1 | 0.68 | 0.008997 |
| P19783 | Cytochrome c oxidase subunit 4 isoform 1, mitochondrial | 0.23 | 0.00906 |
| Q9WUK2 | Eukaryotic translation initiation factor 4H | 0.20 | 0.009136 |
| P15532 | Nucleoside diphosphate kinase A | -0.48 | 0.009219 |
| Q91YP0 | L-2-hydroxyglutarate dehydrogenase, mitochondrial | 0.18 | 0.009233 |
| Q99LP6 | GrpE protein homolog 1, mitochondrial | 0.23 | 0.009284 |
| O88983 | Syntaxin-8 | 0.97 | 0.009325 |
| P52479 | Ubiquitin carboxyl-terminal hydrolase 10 | 0.44 | 0.009359 |
| Q91XV3 | Brain acid soluble protein 1 | 1.00 | 0.00936 |

| | | | |
|---|---|---|---|
| Q62095 | ATP-dependent RNA helicase DDX3Y;Putative ATP-dependent RNA helicase Pl10 | 0.27 | 0.009401 |
| Q62425 | Cytochrome c oxidase subunit NDUFA4 | -0.33 | 0.00948 |
| Q8C166 | Copine-1 | 0.45 | 0.009485 |
| Q8K1R3 | Polyribonucleotide nucleotidyltransferase 1, mitochondrial | 0.28 | 0.009485 |
| Q921W0 | Charged multivesicular body protein 1a | -0.24 | 0.009526 |
| Q6NZB0 | DnaJ homolog subfamily C member 8 | -0.34 | 0.009546 |
| Q8BKZ9 | Pyruvate dehydrogenase protein X component, mitochondrial | 0.25 | 0.009614 |
| Q9Z1G4 | V-type proton ATPase 116 kDa subunit a isoform 1 | 0.33 | 0.009619 |
| P48193 | Protein 4.1 | 0.23 | 0.009647 |
| Q9DCB8 | Iron-sulfur cluster assembly 2 homolog, mitochondrial | 0.32 | 0.009766 |
| Q9JHJ0 | Tropomodulin-3 | 0.30 | 0.009868 |
| Q3UQ84 | Threonine--tRNA ligase, mitochondrial | 0.40 | 0.009878 |
| P43006 | Excitatory amino acid transporter 2 | 0.45 | 0.009963 |
| Q9D735 | Uncharacterized protein C19orf43 homolog | 1.27 | 0.010062 |
| Q64520 | Guanylate kinase | -0.41 | 0.010088 |
| Q6P9Q4 | FH1/FH2 domain-containing protein 1 | 0.60 | 0.010192 |
| Q8R3B1 | 1-phosphatidylinositol 4,5-bisphosphate phosphodiesterase delta-1 | 0.44 | 0.010203 |
| Q3URD3 | Sarcolemmal membrane-associated protein | -0.31 | 0.010204 |
| Q8BUY5 | Complex I assembly factor TIMMDC1, mitochondrial | 0.42 | 0.010246 |
| P60867 | 40S ribosomal protein S20 | 0.36 | 0.010361 |
| Q8BIJ7 | RUN and FYVE domain-containing protein 1 | 0.21 | 0.010366 |
| Q9WV98 | Mitochondrial import inner membrane translocase subunit Tim9 | 0.21 | 0.010367 |
| Q6PGH1 | Protein BUD31 homolog | 0.39 | 0.010378 |
| Q61187 | Tumor susceptibility gene 101 protein | 0.21 | 0.010467 |
| P62823 | Ras-related protein Rab-3C | 1.00 | 0.010479 |
| Q3TZ89 | Protein transport protein Sec31B | -0.58 | 0.010504 |
| O55128 | Histone deacetylase complex subunit SAP18 | -0.28 | 0.010612 |
| Q61074 | Protein phosphatase 1G | 0.22 | 0.010628 |
| Q6PAM0 | 5-AMP-activated protein kinase subunit beta-2 | 0.48 | 0.01074 |
| O88685 | 26S protease regulatory subunit 6A | 0.32 | 0.010784 |
| O08599 | Syntaxin-binding protein 1 | 0.39 | 0.010902 |
| Q922D8 | C-1-tetrahydrofolate synthase, cytoplasmic;Methylenetetrahydrofolate dehydrogenase;Methenyltetrahydrofolate cyclohydrolase;Formyltetrahydrofolate synthetase;C-1-tetrahydrofolate synthase, cytoplasmic, N-terminally processed | 0.19 | 0.010926 |
| Q64152 | Transcription factor BTF3 | 0.51 | 0.010986 |

| | | | |
|---|---|---|---|
| O88952 | Protein lin-7 homolog C | 0.48 | 0.010992 |
| P56382 | ATP synthase subunit epsilon, mitochondrial | -0.37 | 0.011055 |
| Q9D820 | Prolyl-tRNA synthetase associated domain-containing protein 1 | 0.28 | 0.011062 |
| P61922 | 4-aminobutyrate aminotransferase, mitochondrial | 0.40 | 0.011086 |
| Q99KC8 | von Willebrand factor A domain-containing protein 5A | 0.25 | 0.011151 |
| Q61029 | Lamina-associated polypeptide 2, isoforms beta/delta/epsilon/gamma | 0.17 | 0.011164 |
| O55100 | Synaptogyrin-1 | 0.27 | 0.011206 |
| Q99K51 | Plastin-3 | 0.21 | 0.011254 |
| Q9DBL1 | Short/branched chain specific acyl-CoA dehydrogenase, mitochondrial | 0.22 | 0.011317 |
| Q3TL44 | NLR family member X1 | 0.64 | 0.011334 |
| Q5SX39 | Myosin-4 | -0.61 | 0.01135 |
| Q9JK81 | UPF0160 protein MYG1, mitochondrial | 0.41 | 0.011353 |
| P23953 | Carboxylesterase 1C | -0.33 | 0.011359 |
| Q8VBV7 | COP9 signalosome complex subunit 8 | 0.19 | 0.011382 |
| Q5ND52 | rRNA methyltransferase 3, mitochondrial | 0.35 | 0.011405 |
| Q9CYG7 | Mitochondrial import receptor subunit TOM34 | 0.29 | 0.011426 |
| P97821 | Dipeptidyl peptidase 1;Dipeptidyl peptidase 1 exclusion domain chain;Dipeptidyl peptidase 1 heavy chain;Dipeptidyl peptidase 1 light chain | 0.28 | 0.011518 |
| P14733 | Lamin-B1 | 0.16 | 0.011563 |
| Q9QUR7 | Peptidyl-prolyl cis-trans isomerase NIMA-interacting 1 | 0.29 | 0.011595 |
| O35566 | CD151 antigen | -0.33 | 0.011657 |
| Q9DA03 | Complex III assembly factor LYRM7 | 0.23 | 0.011674 |
| Q6ZQ73 | Cullin-associated NEDD8-dissociated protein 2 | 0.26 | 0.011733 |
| Q9WU79 | Proline dehydrogenase 1, mitochondrial | 0.50 | 0.011736 |
| P17095 | High mobility group protein HMG-I/HMG-Y | -0.55 | 0.011799 |
| P04925 | Major prion protein | 0.32 | 0.01192 |
| Q61334 | B-cell receptor-associated protein 29 | 0.34 | 0.011964 |
| Q8CGC7 | Bifunctional glutamate/proline--tRNA ligase;Glutamate--tRNA ligase;Proline--tRNA ligase | 0.17 | 0.011982 |
| P61957 | Small ubiquitin-related modifier 2 | -0.22 | 0.012145 |
| Q9JJK2 | LanC-like protein 2 | -0.45 | 0.012267 |
| Q3U5Q7 | UMP-CMP kinase 2, mitochondrial | 0.20 | 0.012279 |
| Q91YJ3 | Thymocyte nuclear protein 1 | 0.28 | 0.012283 |
| Q9D0K2 | Succinyl-CoA:3-ketoacid coenzyme A transferase 1, mitochondrial | 0.17 | 0.01237 |
| Q3UIU2 | NADH dehydrogenase [ubiquinone] 1 beta subcomplex subunit 6 | 0.28 | 0.012384 |
| Q9QXD8 | LIM domain-containing protein 1 | -0.23 | 0.01246 |
| Q8C0E3 | Tripartite motif-containing protein 47 | 0.49 | 0.012473 |
| O35114 | Lysosome membrane protein 2 | -0.65 | 0.012476 |

| | | | |
|---|---|---|---|
| P60670 | Nuclear protein localization protein 4 homolog | 0.34 | 0.012615 |
| O08579 | Emerin | 0.37 | 0.012646 |
| Q9Z2W0 | Aspartyl aminopeptidase | 0.26 | 0.012675 |
| Q6ZWQ0 | Nesprin-2 | 0.69 | 0.012692 |
| Q9CZR3 | Mitochondrial import receptor subunit TOM40B | -0.47 | 0.0127 |
| Q8BRK8 | 5-AMP-activated protein kinase catalytic subunit alpha-2 | 0.25 | 0.012737 |
| Q9CZJ2 | Heat shock 70 kDa protein 12B | 0.21 | 0.012738 |
| Q8BH43 | Wiskott-Aldrich syndrome protein family member 2 | 0.27 | 0.012739 |
| Q9WTM5 | RuvB-like 2 | 0.28 | 0.012756 |
| Q3UUI3 | Acyl-coenzyme A thioesterase THEM4 | 0.36 | 0.01285 |
| D3YXK2 | Scaffold attachment factor B1 | 0.38 | 0.012887 |
| Q5XJY4 | Presenilins-associated rhomboid-like protein, mitochondrial;P-beta | 0.56 | 0.012932 |
| Q9EQ20 | Methylmalonate-semialdehyde dehydrogenase [acylating], mitochondrial | 0.16 | 0.012998 |
| Q9D7P6 | Iron-sulfur cluster assembly enzyme ISCU, mitochondrial | -0.28 | 0.013039 |
| Q3ULF4 | Paraplegin | 0.20 | 0.013058 |
| Q60710 | Deoxynucleoside triphosphate triphosphohydrolase SAMHD1 | 0.37 | 0.013062 |
| Q8R081 | Heterogeneous nuclear ribonucleoprotein L | 0.35 | 0.013127 |
| P97355 | Spermine synthase | -0.42 | 0.013136 |
| O55013 | Trafficking protein particle complex subunit 3 | -0.27 | 0.013254 |
| E9Q401 | Ryanodine receptor 2 | 0.25 | 0.013272 |
| Q9CQY6 | Ubiquinol-cytochrome-c reductase complex assembly factor 2 | 0.23 | 0.01329 |
| Q64337 | Sequestosome-1 | 0.59 | 0.013327 |
| B2RRE7 | OTU domain-containing protein 4 | 0.23 | 0.013332 |
| Q8BWA5 | Kelch-like protein 31 | -0.71 | 0.013332 |
| P29758 | Ornithine aminotransferase, mitochondrial | 0.30 | 0.013336 |
| P18608 | Non-histone chromosomal protein HMG-14 | -0.43 | 0.013419 |
| P01899 | H-2 class I histocompatibility antigen, D-B alpha chain;H-2 class I histocompatibility antigen, alpha chain;H-2 class I histocompatibility antigen, L-D alpha chain | 0.18 | 0.013456 |
| Q9ES97 | Reticulon-3 | 0.33 | 0.013489 |
| P62309 | Small nuclear ribonucleoprotein G | 0.50 | 0.013542 |
| E9Q7G0 | Nuclear mitotic apparatus protein 1 | 0.21 | 0.013582 |
| Q7TMB8 | Cytoplasmic FMR1-interacting protein 1;Cytoplasmic FMR1-interacting protein 2 | 0.22 | 0.013641 |
| P35505 | Fumarylacetoacetase | 0.33 | 0.013645 |
| Q99JR5 | Tubulointerstitial nephritis antigen-like | 0.22 | 0.013904 |

| | | | |
|---|---|---|---|
| P62141 | Serine/threonine-protein phosphatase PP1-beta catalytic subunit | 0.50 | 0.013916 |
| Q3THG9 | Alanyl-tRNA editing protein Aarsd1 | 0.26 | 0.013953 |
| Q61239 | Protein farnesyltransferase/geranylgeranyltransferase type-1 subunit alpha | 0.24 | 0.013974 |
| Q61411 | GTPase HRas;GTPase HRas, N-terminally processed | -0.32 | 0.014002 |
| Q99M01 | Phenylalanine--tRNA ligase, mitochondrial | -0.32 | 0.014034 |
| Q9CR61 | NADH dehydrogenase [ubiquinone] 1 beta subcomplex subunit 7 | 0.17 | 0.014095 |
| Q3UV70 | [Pyruvate dehydrogenase [acetyl-transferring]]-phosphatase 1, mitochondrial | 0.23 | 0.014097 |
| P47955 | 60S acidic ribosomal protein P1 | 0.46 | 0.014173 |
| P84096 | Rho-related GTP-binding protein RhoG | 0.25 | 0.014295 |
| P61961 | Ubiquitin-fold modifier 1 | -0.49 | 0.014454 |
| Q99NB1 | Acetyl-coenzyme A synthetase 2-like, mitochondrial | 0.30 | 0.014575 |
| P97370 | Sodium/potassium-transporting ATPase subunit beta-3 | 0.53 | 0.014611 |
| Q9CWK8 | Sorting nexin-2 | 0.16 | 0.014685 |
| P97379 | Ras GTPase-activating protein-binding protein 2 | 0.27 | 0.014691 |
| Q99KR8 | Plasma alpha-L-fucosidase | 0.32 | 0.014693 |
| Q65CL1 | Catenin alpha-3 | 0.20 | 0.014695 |
| P0C872 | JmjC domain-containing protein 7 | 0.43 | 0.014722 |
| Q8VDQ8 | NAD-dependent protein deacetylase sirtuin-2 | 0.19 | 0.014746 |
| P84084 | ADP-ribosylation factor 5 | -0.29 | 0.014784 |
| Q8BU33 | Acetolactate synthase-like protein | 0.22 | 0.014789 |
| Q9Z0X1 | Apoptosis-inducing factor 1, mitochondrial | 0.17 | 0.014835 |
| Q921M7 | Protein FAM49B | -0.28 | 0.014852 |
| Q68FF6 | ARF GTPase-activating protein GIT1 | -0.21 | 0.014874 |
| Q9CQ60 | 6-phosphogluconolactonase | 0.20 | 0.014881 |
| Q61037 | Tuberin | 0.32 | 0.014968 |
| Q9Z0U1 | Tight junction protein ZO-2 | -0.25 | 0.015023 |
| Q80W00 | Serine/threonine-protein phosphatase 1 regulatory subunit 10 | 0.83 | 0.015116 |
| P60060 | Protein transport protein Sec61 subunit gamma | 0.70 | 0.015143 |
| Q70FJ1 | A-kinase anchor protein 9 | 0.26 | 0.015144 |
| Q9CQU0 | Thioredoxin domain-containing protein 12 | 0.30 | 0.015166 |
| P09103 | Protein disulfide-isomerase | 0.18 | 0.015192 |
| Q9ESW4 | Acylglycerol kinase, mitochondrial | 0.37 | 0.015206 |
| Q6P5C5 | Single-strand selective monofunctional uracil DNA glycosylase | 0.87 | 0.015207 |
| P48410 | ATP-binding cassette sub-family D member 1 | 0.44 | 0.015223 |
| Q64213 | Splicing factor 1 | 0.55 | 0.015288 |
| Q9CQV8 | 14-3-3 protein beta/alpha;14-3-3 protein beta/alpha, N-terminally processed | 0.25 | 0.015594 |

| | | | |
|---|---|---|---|
| Q8R3F5 | Malonyl-CoA-acyl carrier protein transacylase, mitochondrial | 0.42 | 0.015618 |
| Q6P8J7 | Creatine kinase S-type, mitochondrial | 0.17 | 0.01571 |
| Q9CYR0 | Single-stranded DNA-binding protein, mitochondrial | -0.37 | 0.015739 |
| Q8C3X2 | Coiled-coil domain-containing protein 90B, mitochondrial | -0.49 | 0.015775 |
| Q91V12 | Cytosolic acyl coenzyme A thioester hydrolase | 0.33 | 0.015854 |
| Q9R0U0 | Serine/arginine-rich splicing factor 10 | 0.48 | 0.015948 |
| Q8R326 | Paraspeckle component 1 | 0.66 | 0.016029 |
| P39054 | Dynamin-2 | 0.18 | 0.016029 |
| Q9JJ11 | Transforming acidic coiled-coil-containing protein 3 | -0.44 | 0.016087 |
| O88667 | GTP-binding protein RAD | 0.20 | 0.016149 |
| Q8K1A5 | Transmembrane protein 41B | 0.21 | 0.016179 |
| Q9D2U9 | Histone H2B type 3-A;Histone H2B type 3-B | 0.34 | 0.016253 |
| Q5DTJ9 | Myopalladin | 0.18 | 0.016277 |
| Q99PW4 | TP53-regulating kinase | 0.40 | 0.016292 |
| O35972 | 39S ribosomal protein L23, mitochondrial | 0.20 | 0.016302 |
| Q61292 | Laminin subunit beta-2 | 0.17 | 0.016393 |
| Q8BU14 | Translocation protein SEC62 | -0.35 | 0.016532 |
| P06837 | Neuromodulin | 0.97 | 0.016631 |
| Q8CGV9 | Teashirt homolog 3 | -0.52 | 0.016681 |
| Q6ZWY8 | Thymosin beta-10 | 0.34 | 0.016782 |
| Q8K4L3 | Supervillin | 0.16 | 0.017011 |
| Q9D1I6 | 39S ribosomal protein L14, mitochondrial | 0.20 | 0.017112 |
| P70349 | Histidine triad nucleotide-binding protein 1 | 0.31 | 0.017134 |
| Q9QZD9 | Eukaryotic translation initiation factor 3 subunit I | 0.18 | 0.01725 |
| B2RSH2 | Guanine nucleotide-binding protein G(i) subunit alpha-1 | 0.82 | 0.017288 |
| O35350 | Calpain-1 catalytic subunit | 0.44 | 0.017355 |
| Q8C7H1 | Methylmalonic aciduria type A homolog, mitochondrial | 0.46 | 0.017356 |
| P51881 | ADP/ATP translocase 2;ADP/ATP translocase 2, N-terminally processed | 0.15 | 0.017361 |
| Q08091 | Calponin-1 | -0.61 | 0.017405 |
| P54822 | Adenylosuccinate lyase | 0.14 | 0.017446 |
| Q8R086 | Sulfite oxidase, mitochondrial | 0.19 | 0.017494 |
| P43406 | Integrin alpha-V;Integrin alpha-V heavy chain;Integrin alpha-V light chain | 0.34 | 0.017499 |
| Q80W21 | Glutathione S-transferase Mu 7 | 0.19 | 0.017515 |
| Q9Z2G9 | Oxidoreductase HTATIP2 | 0.35 | 0.017622 |
| Q9QXK3 | Coatomer subunit gamma-2 | 0.54 | 0.017711 |
| O09005 | Sphingolipid delta(4)-desaturase DES1 | 0.64 | 0.017775 |
| Q91VK1 | Basic leucine zipper and W2 domain-containing protein 2 | 0.27 | 0.017796 |
| Q8C878 | NEDD8-activating enzyme E1 catalytic subunit | 0.50 | 0.017862 |

| | | | |
|---|---|---|---|
| P62259 | 14-3-3 protein epsilon | -0.19 | 0.017941 |
| Q9CPR5 | 39S ribosomal protein L15, mitochondrial | 0.25 | 0.017965 |
| P61982 | 14-3-3 protein gamma;14-3-3 protein gamma, N-terminally processed | -0.20 | 0.018006 |
| Q9CXW2 | 28S ribosomal protein S22, mitochondrial | 0.15 | 0.01808 |
| Q60854 | Serpin B6 | -0.18 | 0.018413 |
| Q3URE1 | Acyl-CoA synthetase family member 3, mitochondrial | 0.28 | 0.018453 |
| Q91YE3 | Egl nine homolog 1 | 0.40 | 0.018472 |
| P60603 | Reactive oxygen species modulator 1 | 0.54 | 0.018537 |
| Q9CYR6 | Phosphoacetylglucosamine mutase | -0.22 | 0.018737 |
| P43276 | Histone H1.5 | 0.30 | 0.018924 |
| Q6PDI5 | Proteasome-associated protein ECM29 homolog | 0.16 | 0.018998 |
| P60761 | Neurogranin;NEUG(55-78) | 1.18 | 0.019023 |
| P46412 | Glutathione peroxidase 3 | -0.53 | 0.01904 |
| P16675 | Lysosomal protective protein;Lysosomal protective protein 32 kDa chain;Lysosomal protective protein 20 kDa chain | -0.21 | 0.019163 |
| Q8BJU9 | Peptide chain release factor 1-like, mitochondrial | 0.82 | 0.01925 |
| Q5GIG6 | Serine/threonine-protein kinase TNNI3K | 0.23 | 0.019277 |
| Q9JIG7 | Coiled-coil domain-containing protein 22 | 0.45 | 0.019293 |
| Q9EQ06 | Estradiol 17-beta-dehydrogenase 11 | 0.25 | 0.019416 |
| P00493 | Hypoxanthine-guanine phosphoribosyltransferase | 0.23 | 0.019444 |
| Q8CHR6 | Dihydropyrimidine dehydrogenase [NADP(+)] | 0.23 | 0.019493 |
| Q8VCK3 | Tubulin gamma-2 chain;Tubulin gamma-1 chain | 0.28 | 0.019672 |
| O35685 | Nuclear migration protein nudC | 0.19 | 0.019723 |
| Q62165 | Dystroglycan;Alpha-dystroglycan;Beta-dystroglycan | -0.24 | 0.019758 |
| Q9D1Q6 | Endoplasmic reticulum resident protein 44 | 0.30 | 0.019824 |
| Q01815 | Voltage-dependent L-type calcium channel subunit alpha-1C;Voltage-dependent L-type calcium channel subunit alpha-1D | -0.29 | 0.019847 |
| Q9CQ75 | NADH dehydrogenase [ubiquinone] 1 alpha subcomplex subunit 2 | 0.44 | 0.019877 |
| P63001 | Ras-related C3 botulinum toxin substrate 1;Ras-related C3 botulinum toxin substrate 2;Ras-related C3 botulinum toxin substrate 3 | -0.31 | 0.019986 |
| Q920Q6 | RNA-binding protein Musashi homolog 2 | 0.44 | 0.020002 |
| Q99KP6 | Pre-mRNA-processing factor 19 | -0.20 | 0.020103 |
| Q99MR9 | Protein phosphatase 1 regulatory subunit 3A | -0.24 | 0.020237 |
| Q99MN9 | Propionyl-CoA carboxylase beta chain, mitochondrial | 0.17 | 0.020237 |
| Q501J6 | Probable ATP-dependent RNA helicase DDX17 | 0.15 | 0.02028 |
| P39447 | Tight junction protein ZO-1 | 0.23 | 0.020426 |
| Q8C6K9 | Collagen alpha-6(VI) chain | 0.41 | 0.020434 |
| Q80WW9 | DDRGK domain-containing protein 1 | 0.85 | 0.020451 |
| Q9DB60 | Prostamide/prostaglandin F synthase | -0.47 | 0.020467 |

| | | | |
|---|---|---|---|
| Q80VD1 | Protein FAM98B | 1.66 | 0.020542 |
| Q5EBG6 | Heat shock protein beta-6 | -0.32 | 0.020602 |
| Q8R4S0 | Protein phosphatase 1 regulatory subunit 14C | 1.07 | 0.020919 |
| P23242 | Gap junction alpha-1 protein | 0.36 | 0.020941 |
| P08032 | Spectrin alpha chain, erythrocytic 1 | 0.16 | 0.021075 |
| O55135 | Eukaryotic translation initiation factor 6 | 0.17 | 0.02116 |
| P53810 | Phosphatidylinositol transfer protein alpha isoform | 0.40 | 0.021245 |
| Q8CAK1 | Putative transferase CAF17 homolog, mitochondrial | 0.56 | 0.021304 |
| Q9D554 | Splicing factor 3A subunit 3 | 0.17 | 0.021378 |
| Q6A0D4 | Raftlin | 0.33 | 0.02149 |
| A3KMP2 | Tetratricopeptide repeat protein 38 | 0.15 | 0.021573 |
| Q8BGN2 | UPF0462 protein C4orf33 homolog | -0.20 | 0.02167 |
| O08585 | Clathrin light chain A | 0.18 | 0.021684 |
| P49586 | Choline-phosphate cytidylyltransferase A | -0.21 | 0.021696 |
| Q8BK30 | NADH dehydrogenase [ubiquinone] flavoprotein 3, mitochondrial | -0.42 | 0.021702 |
| Q3ULD5 | Methylcrotonoyl-CoA carboxylase beta chain, mitochondrial | 0.15 | 0.021712 |
| P35285 | Ras-related protein Rab-22A | -0.29 | 0.021719 |
| Q8BKC5 | Importin-5 | 0.17 | 0.021737 |
| Q8BHL3 | TBC1 domain family member 10B | 0.35 | 0.021899 |
| Q62087 | Serum paraoxonase/lactonase 3 | 0.71 | 0.021977 |
| P20918 | Plasminogen;Plasmin heavy chain A;Activation peptide;Angiostatin;Plasmin heavy chain A, short form;Plasmin light chain B | -0.15 | 0.021994 |
| Q3U0S6 | Ras-interacting protein 1 | 0.21 | 0.021998 |
| E9Q9K5 | Triadin | -0.47 | 0.022052 |
| Q80X50 | Ubiquitin-associated protein 2-like | 0.21 | 0.022117 |
| P61759 | Prefoldin subunit 3 | -0.22 | 0.022147 |
| Q60866 | Phosphotriesterase-related protein | 0.44 | 0.022204 |
| Q02257 | Junction plakoglobin | 0.28 | 0.022272 |
| P10922 | Histone H1.0;Histone H1.0, N-terminally processed | 0.45 | 0.022367 |
| Q61247 | Alpha-2-antiplasmin | -0.34 | 0.022377 |
| Q3UFY7 | 7-methylguanosine phosphate-specific 5-nucleotidase | 0.19 | 0.022619 |
| Q8BH86 | UPF0317 protein C14orf159 homolog, mitochondrial | 0.38 | 0.022695 |
| Q8VEE0 | Ribulose-phosphate 3-epimerase | 0.60 | 0.022789 |
| P03921 | NADH-ubiquinone oxidoreductase chain 5 | -0.28 | 0.022805 |
| Q8K4G1 | Latent-transforming growth factor beta-binding protein 4 | 0.29 | 0.02285 |
| P42208 | Septin-2 | 0.17 | 0.023261 |
| P83917 | Chromobox protein homolog 1 | -0.98 | 0.02348 |
| Q99JT1 | Glutamyl-tRNA(Gln) amidotransferase subunit B, mitochondrial | 0.25 | 0.023515 |

| | | | |
|---|---|---|---|
| O08749 | Dihydrolipoyl dehydrogenase, mitochondrial | 0.20 | 0.023685 |
| P49813 | Tropomodulin-1 | -0.26 | 0.023788 |
| Q9EPU0 | Regulator of nonsense transcripts 1 | -0.51 | 0.023869 |
| P40336 | Vacuolar protein sorting-associated protein 26A | 0.54 | 0.024019 |
| Q8CI59 | Metalloreductase STEAP3 | 0.23 | 0.024052 |
| P10126 | Elongation factor 1-alpha 1 | -0.19 | 0.024106 |
| Q91W90 | Thioredoxin domain-containing protein 5 | 0.54 | 0.024315 |
| P97390 | Vacuolar protein sorting-associated protein 45 | 0.35 | 0.024355 |
| A2AIL4 | NADH dehydrogenase (ubiquinone) complex I, assembly factor 6 | 0.16 | 0.024377 |
| Q8BUK6 | Protein Hook homolog 3 | 0.39 | 0.024413 |
| Q8BWW9 | Serine/threonine-protein kinase N2 | 1.03 | 0.024458 |
| P14131 | 40S ribosomal protein S16 | -0.17 | 0.024466 |
| Q60598 | Src substrate cortactin | -0.26 | 0.024511 |
| Q6ZQ38 | Cullin-associated NEDD8-dissociated protein 1 | 0.27 | 0.024638 |
| Q9D8B3 | Charged multivesicular body protein 4b | 0.15 | 0.024638 |
| O70433 | Four and a half LIM domains protein 2 | -0.16 | 0.024729 |
| Q922Q9 | Chitinase domain-containing protein 1 | 0.41 | 0.024786 |
| Q9CZN7 | Serine hydroxymethyltransferase | 0.33 | 0.024914 |
| Q9JIY5 | Serine protease HTRA2, mitochondrial | 0.37 | 0.025067 |
| Q9Z2I9 | Succinyl-CoA ligase [ADP-forming] subunit beta, mitochondrial | 0.14 | 0.025236 |
| Q99LD4 | COP9 signalosome complex subunit 1 | 0.24 | 0.025272 |
| Q8BQZ4 | Ral GTPase-activating protein subunit beta | 0.32 | 0.025347 |
| Q8VEJ9 | Vacuolar protein sorting-associated protein 4A | 0.33 | 0.025357 |
| Q8BTZ7 | Mannose-1-phosphate guanyltransferase beta | 0.27 | 0.025384 |
| Q9Z2M7 | Phosphomannomutase 2 | 0.41 | 0.02552 |
| F6TQD1 | Probable E3 SUMO-protein ligase RNF212 | -0.25 | 0.025555 |
| Q8K3W0 | BRCA1-A complex subunit BRE | 0.24 | 0.025586 |
| Q9JJ28 | Protein flightless-1 homolog | 0.24 | 0.025599 |
| Q9CQ89 | Protein CutA | 0.25 | 0.025646 |
| Q9JL62 | Glycolipid transfer protein | 0.68 | 0.025739 |
| Q62093 | Serine/arginine-rich splicing factor 2 | 0.20 | 0.02585 |
| P62257 | Ubiquitin-conjugating enzyme E2 H | 0.34 | 0.025912 |
| Q9WUR9 | Adenylate kinase 4, mitochondrial | 0.25 | 0.026178 |
| Q6ZWN5 | 40S ribosomal protein S9 | -0.31 | 0.026235 |
| P09528 | Ferritin heavy chain;Ferritin heavy chain, N-terminally processed | -0.24 | 0.026246 |
| Q8R0N6 | Hydroxyacid-oxoacid transhydrogenase, mitochondrial | 0.28 | 0.026301 |
| O55222 | Integrin-linked protein kinase | 0.23 | 0.026334 |
| Q9D6W8 | Uncharacterized protein C17orf59 homolog | 0.23 | 0.026346 |
| Q8BH95 | Enoyl-CoA hydratase, mitochondrial | 0.21 | 0.026474 |

| | | | |
|---|---|---|---|
| Q9CWG8 | NADH dehydrogenase [ubiquinone] complex I, assembly factor 7 | 0.30 | 0.026482 |
| Q01853 | Transitional endoplasmic reticulum ATPase | 0.19 | 0.026506 |
| Q80VP1 | Epsin-1 | 0.64 | 0.026547 |
| Q8BG05 | Heterogeneous nuclear ribonucleoprotein A3 | 0.28 | 0.026628 |
| Q9JHU2 | Palmdelphin | 0.34 | 0.026665 |
| P11352 | Glutathione peroxidase 1 | 0.15 | 0.026713 |
| Q6P3D0 | U8 snoRNA-decapping enzyme | -0.26 | 0.026746 |
| O35381 | Acidic leucine-rich nuclear phosphoprotein 32 family member A | 0.13 | 0.026897 |
| Q923D5 | WW domain-binding protein 11 | 0.75 | 0.027043 |
| Q6PFR5 | Transformer-2 protein homolog alpha;Transformer-2 protein homolog beta | -0.22 | 0.027073 |
| Q925F2 | Endothelial cell-selective adhesion molecule | 0.22 | 0.027243 |
| Q61937 | Nucleophosmin | 0.31 | 0.027339 |
| Q99LB6 | Methionine adenosyltransferase 2 subunit beta | 0.51 | 0.027401 |
| Q8BTI8 | Serine/arginine repetitive matrix protein 2 | 0.36 | 0.027413 |
| Q9R062 | Glycogenin-1 | 0.16 | 0.027506 |
| P56501 | Mitochondrial uncoupling protein 3 | 0.16 | 0.027645 |
| P19096 | Fatty acid synthase;[Acyl-carrier-protein] S-acetyltransferase;[Acyl-carrier-protein] S-malonyltransferase;3-oxoacyl-[acyl-carrier-protein] synthase;3-oxoacyl-[acyl-carrier-protein] reductase;3-hydroxyacyl-[acyl-carrier-protein] dehydratase;Enoyl-[acyl-carrier-protein] reductase;Oleoyl-[acyl-carrier-protein] hydrolase | 0.43 | 0.027831 |
| P53811 | Phosphatidylinositol transfer protein beta isoform | 0.30 | 0.028007 |
| P50752 | Troponin T, cardiac muscle | 0.14 | 0.028105 |
| Q99JB2 | Stomatin-like protein 2, mitochondrial | 0.44 | 0.028193 |
| Q9Z204 | Heterogeneous nuclear ribonucleoproteins C1/C2 | 0.15 | 0.028223 |
| Q8CI12 | Smoothelin-like protein 2 | 0.24 | 0.028381 |
| E9QA62 | Leiomodin-3 | 0.89 | 0.028462 |
| Q9CYT6 | Adenylyl cyclase-associated protein 2 | 0.16 | 0.028548 |
| Q8CJ53 | Cdc42-interacting protein 4 | 0.22 | 0.028568 |
| E9Q6P5 | Tetratricopeptide repeat protein 7B | 0.84 | 0.028574 |
| Q9DAT5 | Mitochondrial tRNA-specific 2-thiouridylase 1 | 0.22 | 0.028651 |
| Q61702 | Inter-alpha-trypsin inhibitor heavy chain H1 | 0.18 | 0.028695 |
| O35674 | Disintegrin and metalloproteinase domain-containing protein 19 | 0.77 | 0.028806 |
| P21981 | Protein-glutamine gamma-glutamyltransferase 2 | 0.15 | 0.028947 |
| B2RQC6 | CAD protein;Glutamine-dependent carbamoyl-phosphate synthase;Aspartate carbamoyltransferase;Dihydroorotase | 0.37 | 0.029235 |
| P80314 | T-complex protein 1 subunit beta | 0.24 | 0.029287 |

| | | | |
|---|---|---|---|
| Q9WTX6 | Cullin-1 | -0.19 | 0.029311 |
| P47941 | Crk-like protein | 0.26 | 0.029389 |
| Q8R2G4 | Ecto-ADP-ribosyltransferase 3 | 0.33 | 0.029473 |
| O35215 | D-dopachrome decarboxylase | 0.19 | 0.02959 |
| Q99MN1 | Lysine--tRNA ligase | 0.16 | 0.02962 |
| Q91WM2 | Cat eye syndrome critical region protein 5 homolog | 0.21 | 0.029666 |
| P46467 | Vacuolar protein sorting-associated protein 4B | -0.27 | 0.02978 |
| E9PVA8 | eIF-2-alpha kinase activator GCN1 | 0.20 | 0.029904 |
| Q6P5E4 | UDP-glucose:glycoprotein glucosyltransferase 1 | 0.43 | 0.029915 |
| O35459 | Delta(3,5)-Delta(2,4)-dienoyl-CoA isomerase, mitochondrial | 0.14 | 0.029971 |
| Q99K28 | ADP-ribosylation factor GTPase-activating protein 2 | 0.16 | 0.030027 |
| P62631 | Elongation factor 1-alpha 2 | -0.21 | 0.030238 |
| Q9D0J8 | Parathymosin | 0.17 | 0.03024 |
| P51175 | Protoporphyrinogen oxidase | 0.16 | 0.030322 |
| Q3TZZ7 | Extended synaptotagmin-2 | 0.20 | 0.030402 |
| Q00519 | Xanthine dehydrogenase/oxidase;Xanthine dehydrogenase;Xanthine oxidase | 0.28 | 0.030602 |
| Q9CPV4 | Glyoxalase domain-containing protein 4 | 0.17 | 0.030644 |
| O55111 | Desmoglein-2 | 0.18 | 0.030729 |
| P62320 | Small nuclear ribonucleoprotein Sm D3 | 0.26 | 0.030762 |
| Q9EP52 | Twisted gastrulation protein homolog 1 | 0.65 | 0.03082 |
| Q99JY3 | GTPase IMAP family member 4 | -0.52 | 0.030858 |
| Q8R5H1 | Ubiquitin carboxyl-terminal hydrolase 15 | 0.25 | 0.030978 |
| Q8BWB1 | Synaptopodin 2-like protein | 0.27 | 0.031 |
| P20029 | 78 kDa glucose-regulated protein | 0.15 | 0.031026 |
| P24668 | Cation-dependent mannose-6-phosphate receptor | 0.29 | 0.031126 |
| Q62448 | Eukaryotic translation initiation factor 4 gamma 2 | -0.31 | 0.031277 |
| P63328 | Serine/threonine-protein phosphatase 2B catalytic subunit alpha isoform | 0.33 | 0.031414 |
| Q14AI6 | RNA pseudouridylate synthase domain-containing protein 3 | 0.34 | 0.031421 |
| P34022 | Ran-specific GTPase-activating protein | -0.21 | 0.031444 |
| Q3TMH2 | Secernin-3 | -0.25 | 0.031469 |
| Q64442 | Sorbitol dehydrogenase | 0.13 | 0.031677 |
| Q9DCS9 | NADH dehydrogenase [ubiquinone] 1 beta subcomplex subunit 10 | 0.30 | 0.031696 |
| P68254 | 14-3-3 protein theta | 0.16 | 0.031735 |
| P63254 | Cysteine-rich protein 1 | -0.19 | 0.031911 |
| Q01341 | Adenylate cyclase type 6;Adenylate cyclase type 5 | 0.30 | 0.032108 |
| P57776 | Elongation factor 1-delta | 0.18 | 0.032346 |
| Q8BMS1 | Trifunctional enzyme subunit alpha, mitochondrial;Long-chain enoyl-CoA hydratase;Long chain 3-hydroxyacyl-CoA dehydrogenase | 0.15 | 0.032351 |

| | | | |
|---|---|---|---|
| Q3UH68 | LIM and calponin homology domains-containing protein 1 | 0.18 | 0.032434 |
| Q99KH8 | Serine/threonine-protein kinase 24;Serine/threonine-protein kinase 24 35 kDa subunit;Serine/threonine-protein kinase 24 12 kDa subunit | 0.18 | 0.032675 |
| Q9D0F9 | Phosphoglucomutase-1 | -0.21 | 0.032702 |
| A2APC3 | Probable tubulin polyglutamylase TTLL9 | 0.34 | 0.032703 |
| Q7TNS2 | MICOS complex subunit Mic10 | 0.40 | 0.032759 |
| Q9JJV2 | Profilin-2 | -0.28 | 0.032869 |
| Q923D3 | Prostate androgen-regulated mucin-like protein 1 homolog | 0.43 | 0.032953 |
| O70548 | Telethonin | -0.28 | 0.033207 |
| P15331 | Peripherin | 0.23 | 0.0333 |
| Q8BWY3 | Eukaryotic peptide chain release factor subunit 1 | 0.25 | 0.033493 |
| P97414 | Potassium voltage-gated channel subfamily KQT member 1 | 0.33 | 0.033506 |
| P80315 | T-complex protein 1 subunit delta | 0.20 | 0.033519 |
| P70296 | Phosphatidylethanolamine-binding protein 1;Hippocampal cholinergic neurostimulating peptide | -0.25 | 0.033521 |
| Q5I1X5 | RelA-associated inhibitor | 0.48 | 0.033724 |
| Q9DAR7 | m7GpppX diphosphatase | -0.50 | 0.033763 |
| Q8K3A0 | Iron-sulfur cluster co-chaperone protein HscB, mitochondrial | 0.29 | 0.0339 |
| Q62407 | Striated muscle-specific serine/threonine-protein kinase | 0.15 | 0.033934 |
| Q8BVE3 | V-type proton ATPase subunit H | 0.37 | 0.033953 |
| Q9CR41 | Huntingtin-interacting protein K | -0.23 | 0.034126 |
| P62827 | GTP-binding nuclear protein Ran | -0.21 | 0.034217 |
| P63260 | Actin, cytoplasmic 2;Actin, cytoplasmic 2, N-terminally processed | 0.34 | 0.034288 |
| Q9D0L4 | Uncharacterized aarF domain-containing protein kinase 1 | 0.57 | 0.034395 |
| Q03958 | Prefoldin subunit 6 | 0.37 | 0.034415 |
| P97372 | Proteasome activator complex subunit 2 | -0.24 | 0.034449 |
| Q8CI94 | Glycogen phosphorylase, brain form | 0.16 | 0.034627 |
| Q9WU78 | Programmed cell death 6-interacting protein | 0.15 | 0.034832 |
| Q9D823 | 60S ribosomal protein L37 | -0.52 | 0.034843 |
| P48999 | Arachidonate 5-lipoxygenase | 0.53 | 0.035019 |
| P04945 | Ig kappa chain V-VI region NQ2-6.1 | 0.50 | 0.035072 |
| Q99M71 | Mammalian ependymin-related protein 1 | -0.39 | 0.0354 |
| Q0II04 | Nebulette | 0.14 | 0.035485 |
| Q3U1N2 | Sterol regulatory element-binding protein 2;Processed sterol regulatory element-binding protein 2 | 0.88 | 0.035575 |
| P83940 | Transcription elongation factor B polypeptide 1 | 0.47 | 0.035584 |

| | | | |
|---|---|---|---|
| Q9JIX8 | Apoptotic chromatin condensation inducer in the nucleus | 0.50 | 0.035885 |
| P14602 | Heat shock protein beta-1 | -0.18 | 0.035918 |
| Q9EP89 | Serine beta-lactamase-like protein LACTB, mitochondrial | 0.17 | 0.03623 |
| Q8BP40 | Lysophosphatidic acid phosphatase type 6 | -0.20 | 0.036236 |
| Q61646 | Haptoglobin;Haptoglobin alpha chain;Haptoglobin beta chain | 0.49 | 0.03633 |
| P63276 | 40S ribosomal protein S17 | 0.28 | 0.036381 |
| Q64471 | Glutathione S-transferase theta-1 | -0.21 | 0.036426 |
| Q80U72 | Protein scribble homolog | 0.22 | 0.036589 |
| Q9JHU9 | Inositol-3-phosphate synthase 1 | 0.45 | 0.036711 |
| Q8BZW8 | NHL repeat-containing protein 2 | 0.15 | 0.036855 |
| Q8BL97 | Serine/arginine-rich splicing factor 7 | -0.28 | 0.036996 |
| P97314 | Cysteine and glycine-rich protein 2 | 0.43 | 0.03703 |
| Q4QQM4 | Tumor protein p53-inducible protein 11 | 0.47 | 0.037189 |
| P70362 | Ubiquitin fusion degradation protein 1 homolog | 0.37 | 0.037258 |
| P97930 | Thymidylate kinase | 0.46 | 0.037438 |
| P35293 | Ras-related protein Rab-18 | -0.17 | 0.03749 |
| P53996 | Cellular nucleic acid-binding protein | 0.17 | 0.037523 |
| Q9ES74 | Serine/threonine-protein kinase Nek7 | 0.34 | 0.038566 |
| O35551 | Rab GTPase-binding effector protein 1 | 0.31 | 0.03858 |
| Q9JHS9 | Spliceosome-associated protein CWC15 homolog | -0.79 | 0.038729 |
| Q99LD9 | Translation initiation factor eIF-2B subunit beta | 0.35 | 0.03874 |
| O09110 | Dual specificity mitogen-activated protein kinase kinase 3 | -0.23 | 0.038776 |
| Q3UZA1 | CapZ-interacting protein | 0.26 | 0.038842 |
| Q8BGA8 | Acyl-coenzyme A synthetase ACSM5, mitochondrial | 0.27 | 0.038932 |
| Q7TN29 | Stromal membrane-associated protein 2 | 0.67 | 0.03909 |
| Q3U487 | E3 ubiquitin-protein ligase HECTD3 | 0.24 | 0.039118 |
| Q9QZB7 | Actin-related protein 10 | 0.49 | 0.039174 |
| P15306 | Thrombomodulin | 0.20 | 0.039188 |
| Q5SRX1 | TOM1-like protein 2 | 0.14 | 0.039403 |
| P54116 | Erythrocyte band 7 integral membrane protein | 0.21 | 0.039408 |
| Q8VDT9 | 39S ribosomal protein L50, mitochondrial | -0.22 | 0.039495 |
| Q9CWZ3 | RNA-binding protein 8A | 0.22 | 0.039513 |
| P61211 | ADP-ribosylation factor-like protein 1 | -0.39 | 0.03954 |
| O88533 | Aromatic-L-amino-acid decarboxylase | 0.17 | 0.039557 |
| P19123 | Troponin C, slow skeletal and cardiac muscles | 0.27 | 0.039572 |
| Q93092 | Transaldolase | 0.17 | 0.039863 |
| Q80WJ7 | Protein LYRIC | 0.14 | 0.040039 |
| Q8K2L8 | Trafficking protein particle complex subunit 12 | 0.57 | 0.040073 |
| Q9D1G3 | Protein-cysteine N-palmitoyltransferase HHAT-like protein | 0.14 | 0.040153 |

| | | | |
|---|---|---|---|
| Q14CH7 | Alanine--tRNA ligase, mitochondrial | 0.36 | 0.040261 |
| Q9R0M6 | Ras-related protein Rab-9A | 0.18 | 0.040465 |
| O70572 | Sphingomyelin phosphodiesterase 2 | -0.56 | 0.040474 |
| Q99LC5 | Electron transfer flavoprotein subunit alpha, mitochondrial | 0.16 | 0.040532 |
| Q76MZ3 | Serine/threonine-protein phosphatase 2A 65 kDa regulatory subunit A alpha isoform | 0.29 | 0.040628 |
| P70695 | Fructose-1,6-bisphosphatase isozyme 2 | 0.20 | 0.040696 |
| Q91V41 | Ras-related protein Rab-14 | 0.12 | 0.040741 |
| Q8K1E6 | Alpha-ketoglutarate-dependent dioxygenase alkB homolog 3 | 0.20 | 0.040792 |
| P82348 | Gamma-sarcoglycan | 0.33 | 0.040905 |
| P21300 | Aldose reductase-related protein 1 | 0.26 | 0.040954 |
| Q91VD9 | NADH-ubiquinone oxidoreductase 75 kDa subunit, mitochondrial | 0.19 | 0.041003 |
| Q9CQH7 | Transcription factor BTF3 homolog 4 | -0.31 | 0.04104 |
| Q8CIB5 | Fermitin family homolog 2 | 0.13 | 0.041091 |
| O89116 | Vesicle transport through interaction with t-SNAREs homolog 1A | -0.22 | 0.041106 |
| Q05793 | Basement membrane-specific heparan sulfate proteoglycan core protein;Endorepellin;LG3 peptide | 0.17 | 0.041152 |
| P54797 | Transport and Golgi organization 2 homolog | 0.29 | 0.041183 |
| Q9D6J5 | NADH dehydrogenase [ubiquinone] 1 beta subcomplex subunit 8, mitochondrial | 0.34 | 0.041206 |
| Q9D273 | Cob(I)yrinic acid a,c-diamide adenosyltransferase, mitochondrial | 0.23 | 0.041414 |
| Q99PL5 | Ribosome-binding protein 1 | -0.16 | 0.041469 |
| Q8C2Q3 | RNA-binding protein 14 | 0.33 | 0.04153 |
| Q8BK08 | Transmembrane protein 11, mitochondrial | 0.25 | 0.041541 |
| Q9CR88 | 28S ribosomal protein S14, mitochondrial | -0.50 | 0.041608 |
| Q9WVJ2 | 26S proteasome non-ATPase regulatory subunit 13 | 0.21 | 0.041643 |
| Q9EPL9 | Peroxisomal acyl-coenzyme A oxidase 3 | 0.31 | 0.041925 |
| Q61171 | Peroxiredoxin-2 | -0.21 | 0.041962 |
| Q8BWZ3 | N-alpha-acetyltransferase 25, NatB auxiliary subunit | -0.22 | 0.042002 |
| O08600 | Endonuclease G, mitochondrial | 0.27 | 0.042016 |
| Q9D967 | Magnesium-dependent phosphatase 1 | 0.81 | 0.042217 |
| Q8BFZ1 | Trans-2,3-enoyl-CoA reductase-like | -0.28 | 0.042252 |
| Q8R2H9 | Phosphoethanolamine/phosphocholine phosphatase | 0.20 | 0.042263 |
| O88543 | COP9 signalosome complex subunit 3 | 0.17 | 0.042305 |
| Q6ZPJ3 | E2/E3 hybrid ubiquitin-protein ligase UBE2O | 1.90 | 0.042322 |
| Q8R1H0 | Homeodomain-only protein | 0.20 | 0.042628 |
| P16332 | Methylmalonyl-CoA mutase, mitochondrial | 0.24 | 0.042696 |
| Q99JY4 | TraB domain-containing protein | 0.27 | 0.042697 |

| | | | |
|---|---|---|---|
| Q9D8B4 | NADH dehydrogenase [ubiquinone] 1 alpha subcomplex subunit 11 | -0.21 | 0.042874 |
| Q99J25 | rRNA methyltransferase 1, mitochondrial | 0.17 | 0.042874 |
| Q9Z1Y4 | Thyroid receptor-interacting protein 6 | 0.38 | 0.043511 |
| Q9R0H0 | Peroxisomal acyl-coenzyme A oxidase 1 | 0.76 | 0.043519 |
| Q9CPT4 | Myeloid-derived growth factor | 0.27 | 0.043688 |
| Q60893 | Olfactory receptor 151 | 0.38 | 0.043726 |
| Q6ZWV3 | 60S ribosomal protein L10;60S ribosomal protein L10-like | 0.15 | 0.043788 |
| Q921M4 | Golgin subfamily A member 2 | 0.26 | 0.043855 |
| Q9DBG3 | AP-2 complex subunit beta | 0.25 | 0.043882 |
| P62932 | F-box only protein 40 | -0.24 | 0.043908 |
| Q9DCM2 | Glutathione S-transferase kappa 1 | 0.37 | 0.04398 |
| Q3UGR5 | Haloacid dehalogenase-like hydrolase domain-containing protein 2 | 0.15 | 0.044366 |
| P34928 | Apolipoprotein C-I;Truncated apolipoprotein C-I | -0.45 | 0.044493 |
| P17563 | Selenium-binding protein 1;Selenium-binding protein 2 | 0.12 | 0.044539 |
| Q9CQF8 | Ribosomal protein 63, mitochondrial | 0.30 | 0.044576 |
| B2RY56 | RNA-binding protein 25 | -0.39 | 0.044698 |
| Q8C025 | Cholinephosphotransferase 1 | 0.18 | 0.044724 |
| Q9Z0S1 | 3(2),5-bisphosphate nucleotidase 1 | -0.30 | 0.044755 |
| Q9DCZ4 | Apolipoprotein O | 0.22 | 0.044788 |
| Q9WVJ3 | Carboxypeptidase Q | 0.24 | 0.044797 |
| Q99MQ4 | Asporin | 0.16 | 0.044806 |
| Q9D8S9 | BolA-like protein 1 | 0.40 | 0.0449 |
| P50171 | Estradiol 17-beta-dehydrogenase 8 | 0.17 | 0.045054 |
| Q9ERB0 | Synaptosomal-associated protein 29 | 0.45 | 0.045148 |
| P21844 | Chymase | 0.44 | 0.04546 |
| Q811I0 | ATP synthase mitochondrial F1 complex assembly factor 1 | -0.21 | 0.045648 |
| Q9WV85 | Nucleoside diphosphate kinase 3 | 0.33 | 0.045896 |
| Q9CWM4 | Prefoldin subunit 1 | 0.30 | 0.045919 |
| Q9DBL7 | Bifunctional coenzyme A synthase;Phosphopantetheine adenylyltransferase;Dephospho-CoA kinase | 0.20 | 0.046066 |
| Q9WV92 | Band 4.1-like protein 3;Band 4.1-like protein 3, N-terminally processed | 0.36 | 0.0461 |
| Q8BXK9 | Chloride intracellular channel protein 5 | 0.31 | 0.04613 |
| Q8CIG8 | Protein arginine N-methyltransferase 5 | 0.40 | 0.04635 |
| P58281 | Dynamin-like 120 kDa protein, mitochondrial;Dynamin-like 120 kDa protein, form S1 | 0.12 | 0.046441 |
| Q9CPZ8 | COX assembly mitochondrial protein homolog | -0.26 | 0.046502 |
| Q80TM9 | Nischarin | 0.18 | 0.046788 |
| Q5SVL6 | Rap1 GTPase-activating protein 2 | 0.56 | 0.047121 |
| P10833 | Ras-related protein R-Ras | 0.18 | 0.047158 |

| | | | |
|---|---|---|---|
| Q8BY87 | Ubiquitin carboxyl-terminal hydrolase 47 | 0.22 | 0.047168 |
| Q5ND29 | Rab-interacting lysosomal protein | 0.15 | 0.047176 |
| Q9R0N0 | Galactokinase | -0.29 | 0.04727 |
| Q9DB34 | Charged multivesicular body protein 2a | -0.21 | 0.047339 |
| Q99LX0 | Protein deglycase DJ-1 | 0.18 | 0.047554 |
| P08113 | Endoplasmin | 0.14 | 0.047695 |
| P63011 | Ras-related protein Rab-3A | 0.71 | 0.047758 |
| Q8R3Q2 | Serine/threonine-protein phosphatase 6 regulatory subunit 2 | 0.24 | 0.047786 |
| Q7TQH0 | Ataxin-2-like protein | -0.62 | 0.047838 |
| O70503 | Very-long-chain 3-oxoacyl-CoA reductase | -0.22 | 0.047852 |
| Q9CZ13 | Cytochrome b-c1 complex subunit 1, mitochondrial | 0.28 | 0.048051 |
| Q99LC3 | NADH dehydrogenase [ubiquinone] 1 alpha subcomplex subunit 10, mitochondrial | -0.16 | 0.048075 |
| Q64669 | NAD(P)H dehydrogenase [quinone] 1 | 0.23 | 0.048322 |
| Q9CQN7 | 39S ribosomal protein L41, mitochondrial | 0.45 | 0.048527 |
| P58771 | Tropomyosin alpha-1 chain | 0.16 | 0.048698 |
| Q8BHN3 | Neutral alpha-glucosidase AB | 0.22 | 0.048902 |
| P48428 | Tubulin-specific chaperone A | 0.31 | 0.048965 |
| Q9ERK4 | Exportin-2 | 0.37 | 0.049033 |
| Q80UE6 | Serine/threonine-protein kinase WNK4;Serine/threonine-protein kinase WNK2;Serine/threonine-protein kinase WNK1 | 0.14 | 0.049461 |
| Q69Z23 | Dynein heavy chain 17, axonemal | 0.42 | 0.049678 |
| Q8BX70 | Vacuolar protein sorting-associated protein 13C | 0.35 | 0.049885 |
| P82350 | Alpha-sarcoglycan | 0.16 | 0.04996 |
| Q9D1D4 | Transmembrane emp24 domain-containing protein 10 | 0.32 | 0.050126 |
| Q91VE0 | Long-chain fatty acid transport protein 4 | 0.23 | 0.05034 |
| B1AY13 | Ubiquitin carboxyl-terminal hydrolase 24 | 0.21 | 0.050548 |
| Q69ZX8 | Actin-binding LIM protein 3 | 0.29 | 0.050685 |
| Q9CWJ9 | Bifunctional purine biosynthesis protein PURH;Phosphoribosylaminoimidazolecarboxamide formyltransferase;IMP cyclohydrolase | 0.16 | 0.050746 |
| P63038 | 60 kDa heat shock protein, mitochondrial | 0.13 | 0.050842 |
| Q922Q1 | Mitochondrial amidoxime reducing component 2 | 0.14 | 0.050855 |
| O88746 | Target of Myb protein 1 | 0.12 | 0.051041 |
| Q9D1J3 | SAP domain-containing ribonucleoprotein | 0.23 | 0.051147 |
| Q99KX1 | Myeloid leukemia factor 2 | -0.24 | 0.051223 |
| A2APY7 | NADH dehydrogenase [ubiquinone] 1 alpha subcomplex assembly factor 5 | 0.23 | 0.051803 |
| Q8BL65 | Actin-binding LIM protein 2 | -0.18 | 0.051981 |
| Q9CQJ8 | NADH dehydrogenase [ubiquinone] 1 beta subcomplex subunit 9 | 0.12 | 0.052165 |

| | | | |
|---|---|---|---|
| O09118 | Netrin-1 | 0.27 | 0.052202 |
| P11276 | Fibronectin;Anastellin | 0.13 | 0.052244 |
| O54998 | Peptidyl-prolyl cis-trans isomerase FKBP7 | 0.37 | 0.05247 |
| P31001 | Desmin | -0.15 | 0.052487 |
| Q60870 | Receptor expression-enhancing protein 5 | -0.44 | 0.052539 |
| Q8VCE1 | DnaJ homolog subfamily C member 28 | 0.16 | 0.052589 |
| Q9ET78 | Junctophilin-2 | -0.29 | 0.052844 |
| Q58A65 | C-Jun-amino-terminal kinase-interacting protein 4 | 0.20 | 0.052879 |
| Q60634 | Flotillin-2 | 0.25 | 0.052925 |
| D3Z6Q9 | Bridging integrator 2;Myc box-dependent-interacting protein 1;Amphiphysin | 0.17 | 0.053018 |
| Q8BTE0 | Succinate dehydrogenase assembly factor 4, mitochondrial | -0.22 | 0.053176 |
| Q5SSW2 | Proteasome activator complex subunit 4 | -0.20 | 0.053224 |
| Q9CPY7 | Cytosol aminopeptidase | 0.30 | 0.05331 |
| Q8K2C6 | NAD-dependent protein deacylase sirtuin-5, mitochondrial | 0.12 | 0.053328 |
| Q8C8U0 | Liprin-beta-1;Liprin-beta-2 | 0.27 | 0.053374 |
| P60122 | RuvB-like 1 | 0.16 | 0.05339 |
| Q91WS0 | CDGSH iron-sulfur domain-containing protein 1 | 0.14 | 0.053568 |
| Q8BR70 | Protein YIPF6 | 0.30 | 0.053846 |
| Q61749 | Translation initiation factor eIF-2B subunit delta | 0.21 | 0.053981 |
| P58252 | Elongation factor 2 | 0.12 | 0.05406 |
| Q9JMD0 | BUB3-interacting and GLEBS motif-containing protein ZNF207 | 0.41 | 0.054205 |
| O08739 | AMP deaminase 3 | -0.26 | 0.054241 |
| P61089 | Ubiquitin-conjugating enzyme E2 N | -0.22 | 0.05459 |
| Q9JMH9 | Unconventional myosin-XVIIIa | 0.12 | 0.054675 |
| P32233 | Developmentally-regulated GTP-binding protein 1 | 0.19 | 0.054684 |
| Q9CWX2 | Complex I intermediate-associated protein 30, mitochondrial | 0.13 | 0.054844 |
| Q99J45 | Nuclear receptor-binding protein | 0.33 | 0.054877 |
| P30999 | Catenin delta-1 | 0.30 | 0.054905 |
| Q8VCL2 | Protein SCO2 homolog, mitochondrial | 0.37 | 0.055015 |
| Q9WV96 | Mitochondrial import inner membrane translocase subunit Tim10 B | 0.21 | 0.055161 |
| P14206 | 40S ribosomal protein SA | 0.71 | 0.055182 |
| P55002 | Microfibrillar-associated protein 2 | 0.25 | 0.055577 |
| Q8R0G7 | Protein spinster homolog 1 | 0.29 | 0.055816 |
| Q3V009 | Transmembrane emp24 domain-containing protein 1 | -0.47 | 0.055899 |
| Q8BTY1 | Kynurenine--oxoglutarate transaminase 1 | 0.41 | 0.055906 |
| A6H6E2 | Multimerin-2 | -0.24 | 0.056058 |
| Q8BYJ6 | TBC1 domain family member 4 | 0.60 | 0.056259 |
| Q9CPX7 | 28S ribosomal protein S16, mitochondrial | 0.83 | 0.056448 |

| | | | |
|---|---|---|---|
| P01027 | Complement C3;Complement C3 beta chain;C3-beta-c;Complement C3 alpha chain;C3a anaphylatoxin;Acylation stimulating protein;Complement C3b alpha chain;Complement C3c alpha chain fragment 1;Complement C3dg fragment;Complement C3g fragment;Complement C3d fragment;Complement C3f fragment;Complement C3c alpha chain fragment 2 | -0.12 | 0.056507 |
| Q99LN9 | Deoxyhypusine hydroxylase | 0.28 | 0.056525 |
| P07091 | Protein S100-A4 | 0.33 | 0.0566 |
| P15327 | Bisphosphoglycerate mutase | -0.15 | 0.056627 |
| Q01405 | Protein transport protein Sec23A | 0.23 | 0.056778 |
| P70697 | Uroporphyrinogen decarboxylase | 0.31 | 0.056795 |
| Q60605 | Myosin light polypeptide 6 | -0.13 | 0.05689 |
| Q9CY57 | Chromatin target of PRMT1 protein | 0.67 | 0.057033 |
| P23780 | Beta-galactosidase | 0.45 | 0.057308 |
| Q61792 | LIM and SH3 domain protein 1 | 0.15 | 0.057484 |
| O88448 | Kinesin light chain 2 | 0.17 | 0.057693 |
| Q91VI7 | Ribonuclease inhibitor | 0.19 | 0.057766 |
| O08848 | 60 kDa SS-A/Ro ribonucleoprotein | 0.40 | 0.057789 |
| Q8BKY8 | Transcription termination factor 2, mitochondrial | 0.80 | 0.057833 |
| Q9CQS4 | Solute carrier family 25 member 46 | 0.39 | 0.05813 |
| Q9CYH2 | Redox-regulatory protein FAM213A | 0.35 | 0.05814 |
| E9Q557 | Desmoplakin | 0.12 | 0.058178 |
| P43023 | Cytochrome c oxidase subunit 6A2, mitochondrial | 0.25 | 0.05821 |
| Q9CQF4 | Uncharacterized protein C6orf203 homolog | -0.24 | 0.058218 |
| P97454 | Mothers against decapentaplegic homolog 5;Mothers against decapentaplegic homolog 1 | 0.70 | 0.058367 |
| Q8BJE2 | Butyrophilin-like protein 9 | 0.16 | 0.058448 |
| Q8K1Z0 | Ubiquinone biosynthesis protein COQ9, mitochondrial | 0.13 | 0.058674 |
| Q8QZY1 | Eukaryotic translation initiation factor 3 subunit L | -0.73 | 0.058812 |
| P70170 | ATP-binding cassette sub-family C member 9 | 0.40 | 0.058854 |
| Q5SW19 | Clustered mitochondria protein homolog | 0.27 | 0.058903 |
| Q9WVK4 | EH domain-containing protein 1 | 0.11 | 0.059076 |
| O70435 | Proteasome subunit alpha type-3 | -0.20 | 0.059314 |
| Q07456 | Protein AMBP;Alpha-1-microglobulin;Inter-alpha-trypsin inhibitor light chain;Trypstatin | 0.12 | 0.059337 |
| Q9CPQ3 | Mitochondrial import receptor subunit TOM22 homolog | -0.25 | 0.059434 |
| Q60770 | Syntaxin-binding protein 3 | 0.18 | 0.059504 |
| Q9JIM1 | Equilibrative nucleoside transporter 1 | 0.43 | 0.059576 |
| Q9CQI6 | Coactosin-like protein | -0.19 | 0.059724 |
| Q8BTE5 | Protein CEBPZOS | -0.47 | 0.060009 |

| | | | |
|---|---|---|---|
| Q8K424 | Transient receptor potential cation channel subfamily V member 3 | 0.25 | 0.060026 |
| Q3TIU4 | 2,5-phosphodiesterase 12 | 0.21 | 0.060309 |
| P70335 | Rho-associated protein kinase 1 | 0.21 | 0.060342 |
| B9EJA2 | Cortactin-binding protein 2 | -0.37 | 0.060368 |
| Q9CZ83 | 39S ribosomal protein L55, mitochondrial | 0.20 | 0.060492 |
| P62627 | Dynein light chain roadblock-type 1 | 0.12 | 0.06053 |
| Q8BIW1 | Protein prune homolog | 0.17 | 0.06058 |
| A6H611 | Mitochondrial intermediate peptidase | 0.17 | 0.060981 |
| P21126 | Ubiquitin-like protein 4A | -0.26 | 0.061167 |
| Q8R050 | Eukaryotic peptide chain release factor GTP-binding subunit ERF3A;Eukaryotic peptide chain release factor GTP-binding subunit ERF3B | -0.21 | 0.061314 |
| Q9DD18 | D-tyrosyl-tRNA(Tyr) deacylase 1 | 0.26 | 0.061395 |
| P11531 | Dystrophin | 0.11 | 0.061969 |
| P24452 | Macrophage-capping protein | 0.14 | 0.062046 |
| Q8K268 | ATP-binding cassette sub-family F member 3 | 0.14 | 0.062113 |
| Q8BGK2 | [Protein ADP-ribosylarginine] hydrolase-like protein 1 | 0.42 | 0.062136 |
| Q921F2 | TAR DNA-binding protein 43 | -0.19 | 0.062158 |
| Q9Z2D6 | Methyl-CpG-binding protein 2 | 0.32 | 0.062248 |
| Q9CQV1 | Mitochondrial import inner membrane translocase subunit TIM16 | -0.17 | 0.062614 |
| Q9EQQ9 | Protein O-GlcNAcase | 0.21 | 0.062637 |
| Q9DCF9 | Translocon-associated protein subunit gamma | 0.56 | 0.063005 |
| P31750 | RAC-alpha serine/threonine-protein kinase | 0.28 | 0.063048 |
| Q9D6M3 | Mitochondrial glutamate carrier 1 | 0.21 | 0.063277 |
| Q9CPP6 | NADH dehydrogenase [ubiquinone] 1 alpha subcomplex subunit 5 | -0.21 | 0.063413 |
| Q8VEH3 | ADP-ribosylation factor-like protein 8A | 0.14 | 0.06347 |
| P45377 | Aldose reductase-related protein 2 | -0.54 | 0.063566 |
| P62071 | Ras-related protein R-Ras2 | -0.13 | 0.063788 |
| P35385 | Heat shock protein beta-7 | -0.12 | 0.063878 |
| P70168 | Importin subunit beta-1 | 0.15 | 0.064311 |
| O09111 | NADH dehydrogenase [ubiquinone] 1 beta subcomplex subunit 11, mitochondrial | 0.17 | 0.064529 |
| Q9WV60 | Glycogen synthase kinase-3 beta | 0.40 | 0.064546 |
| Q99MR8 | Methylcrotonoyl-CoA carboxylase subunit alpha, mitochondrial | 0.17 | 0.06471 |
| P26638 | Serine--tRNA ligase, cytoplasmic | 0.12 | 0.06536 |
| Q9CZN8 | Glutamyl-tRNA(Gln) amidotransferase subunit A, mitochondrial | 0.35 | 0.065691 |
| Q9WV35 | Probable C->U-editing enzyme APOBEC-2 | -0.35 | 0.065922 |
| Q3UDE2 | Tubulin--tyrosine ligase-like protein 12 | 0.70 | 0.066087 |
| Q9WUA2 | Phenylalanine--tRNA ligase beta subunit | 0.14 | 0.066102 |

| | | | |
|---|---|---|---|
| P70122 | Ribosome maturation protein SBDS | -0.15 | 0.066157 |
| Q99L27 | GMP reductase 2 | 0.43 | 0.066846 |
| P97351 | 40S ribosomal protein S3a | 0.11 | 0.067088 |
| P26231 | Catenin alpha-1 | 0.12 | 0.067213 |
| Q9D0M3 | Cytochrome c1, heme protein, mitochondrial | 0.12 | 0.067405 |
| P29595 | NEDD8 | -0.56 | 0.067425 |
| Q8BMA6 | Signal recognition particle subunit SRP68 | -0.40 | 0.067474 |
| Q99JR6 | Nicotinamide/nicotinic acid mononucleotide adenylyltransferase 3 | 0.17 | 0.067633 |
| Q3UHD3 | Microtubule-associated tumor suppressor candidate 2 homolog | -0.22 | 0.067818 |
| P00920 | Carbonic anhydrase 2 | -0.26 | 0.067955 |
| Q9D8X1 | Copper homeostasis protein cutC homolog | 0.29 | 0.068571 |
| Q9D7H3 | RNA 3-terminal phosphate cyclase | -0.21 | 0.068742 |
| O35955 | Proteasome subunit beta type-10 | -0.20 | 0.069008 |
| P06151 | L-lactate dehydrogenase A chain | -0.20 | 0.069081 |
| P49935 | Pro-cathepsin H;Cathepsin H mini chain;Cathepsin H;Cathepsin H heavy chain;Cathepsin H light chain | 0.54 | 0.069146 |
| Q9QZ08 | N-acetyl-D-glucosamine kinase | 0.55 | 0.069153 |
| Q9JII6 | Alcohol dehydrogenase [NADP(+)] | 0.37 | 0.069532 |
| Q148W8 | Inactive dual specificity phosphatase 27 | -0.20 | 0.069543 |
| Q8K0L0 | Ankyrin repeat and SOCS box protein 2 | 0.16 | 0.069865 |
| Q99M87 | DnaJ homolog subfamily A member 3, mitochondrial | 0.15 | 0.069871 |
| Q61166 | Microtubule-associated protein RP/EB family member 1 | 0.18 | 0.069899 |
| Q8VBT1 | Beta-taxilin | 0.36 | 0.069956 |
| P51667 | Myosin regulatory light chain 2, ventricular/cardiac muscle isoform | -0.16 | 0.069988 |
| Q00612 | Glucose-6-phosphate 1-dehydrogenase X | 0.20 | 0.07008 |
| Q9D6Z1 | Nucleolar protein 56 | 0.69 | 0.070349 |
| P62960 | Nuclease-sensitive element-binding protein 1 | -0.25 | 0.070415 |
| Q99LR1 | Monoacylglycerol lipase ABHD12 | -0.24 | 0.070653 |
| P13597 | Intercellular adhesion molecule 1 | 0.44 | 0.070766 |
| S4R2P9 | Sodium/calcium exchanger 3 | -0.49 | 0.070986 |
| Q9CZ04 | COP9 signalosome complex subunit 7a | -0.23 | 0.071268 |
| P62322 | U6 snRNA-associated Sm-like protein LSm5 | 0.75 | 0.071586 |
| Q9CXJ1 | Probable glutamate--tRNA ligase, mitochondrial | 0.13 | 0.071745 |
| Q9JJG0 | Transforming acidic coiled-coil-containing protein 2 | -0.17 | 0.071906 |
| Q9CW03 | Structural maintenance of chromosomes protein 3 | 0.16 | 0.071906 |
| O70310 | Glycylpeptide N-tetradecanoyltransferase 1 | -0.18 | 0.071918 |
| P57746 | V-type proton ATPase subunit D | 0.30 | 0.071935 |
| Q9JK53 | Prolargin | 0.23 | 0.071991 |
| Q6PDM2 | Serine/arginine-rich splicing factor 1 | 0.25 | 0.072165 |
| Q69ZR2 | E3 ubiquitin-protein ligase HECTD1 | -0.26 | 0.07234 |

| | | | |
|---|---|---|---|
| Q9Z2I8 | Succinyl-CoA ligase [GDP-forming] subunit beta, mitochondrial | 0.13 | 0.07268 |
| Q91V16 | LYR motif-containing protein 5 | -0.19 | 0.072763 |
| P48962 | ADP/ATP translocase 1 | 0.14 | 0.072901 |
| Q8R0S2 | IQ motif and SEC7 domain-containing protein 1 | -0.57 | 0.073013 |
| Q62219 | Transforming growth factor beta-1-induced transcript 1 protein | 0.33 | 0.073064 |
| Q501J2 | Protein FAM173A | -0.20 | 0.073133 |
| Q99J77 | Sialic acid synthase | 0.20 | 0.073177 |
| Q9CR21 | Acyl carrier protein, mitochondrial | -0.34 | 0.073299 |
| Q9JHL1 | Na(+)/H(+) exchange regulatory cofactor NHE-RF2 | 0.16 | 0.073309 |
| P56812 | Programmed cell death protein 5 | 0.18 | 0.073477 |
| Q9JHI5 | Isovaleryl-CoA dehydrogenase, mitochondrial | 0.12 | 0.073538 |
| P08228 | Superoxide dismutase [Cu-Zn] | -0.21 | 0.07395 |
| Q9D023 | Mitochondrial pyruvate carrier 2 | -0.29 | 0.074144 |
| Q9DCT1 | 1,5-anhydro-D-fructose reductase | 0.16 | 0.074781 |
| Q8BH07 | ADP-ribosylation factor-like protein 6-interacting protein 6 | 0.21 | 0.075083 |
| P17047 | Lysosome-associated membrane glycoprotein 2 | -0.22 | 0.075165 |
| Q80YV4 | Pantothenate kinase 4 | 0.17 | 0.075473 |
| Q91ZA3 | Propionyl-CoA carboxylase alpha chain, mitochondrial | 0.15 | 0.075488 |
| P16546 | Spectrin alpha chain, non-erythrocytic 1 | 0.22 | 0.076021 |
| Q3UTJ2 | Sorbin and SH3 domain-containing protein 2 | 0.16 | 0.076075 |
| Q9Z1N5 | Spliceosome RNA helicase Ddx39b;ATP-dependent RNA helicase DDX39A | 0.13 | 0.076102 |
| Q921U8 | Smoothelin | 0.27 | 0.076138 |
| P45952 | Medium-chain specific acyl-CoA dehydrogenase, mitochondrial | 0.13 | 0.076164 |
| Q9Z0G0 | PDZ domain-containing protein GIPC1 | 0.32 | 0.076275 |
| Q99N87 | 28S ribosomal protein S5, mitochondrial | 0.31 | 0.076533 |
| Q9R0X4 | Acyl-coenzyme A thioesterase 9, mitochondrial;Acyl-coenzyme A thioesterase 10, mitochondrial | 0.25 | 0.077055 |
| Q9Z1G3 | V-type proton ATPase subunit C 1 | 0.23 | 0.077428 |
| Q8K3C3 | Protein LZIC | 0.19 | 0.077494 |
| P05132 | cAMP-dependent protein kinase catalytic subunit alpha | -0.18 | 0.077549 |
| P97450 | ATP synthase-coupling factor 6, mitochondrial | -0.23 | 0.077565 |
| Q8BYM8 | Probable cysteine--tRNA ligase, mitochondrial | 0.29 | 0.077592 |
| Q8R2Q4 | Ribosome-releasing factor 2, mitochondrial | 0.16 | 0.078299 |
| Q8BJY1 | 26S proteasome non-ATPase regulatory subunit 5 | 0.10 | 0.078337 |
| Q9CZL5 | Pterin-4-alpha-carbinolamine dehydratase 2;Pterin-4-alpha-carbinolamine dehydratase | 0.20 | 0.078573 |
| Q99LD8 | N(G),N(G)-dimethylarginine dimethylaminohydrolase 2 | 0.20 | 0.078735 |
| Q8BT42 | Small integral membrane protein 5 | 0.22 | 0.078814 |
| Q8BH35 | Complement component C8 beta chain | 0.47 | 0.079466 |

| | | | |
|---|---|---|---|
| Q8CIM3 | D-2-hydroxyglutarate dehydrogenase, mitochondrial | 0.17 | 0.079518 |
| Q8BP47 | Asparagine--tRNA ligase, cytoplasmic | 0.34 | 0.079552 |
| Q9CQ62 | 2,4-dienoyl-CoA reductase, mitochondrial | -0.13 | 0.079757 |
| Q8BMF3 | NADP-dependent malic enzyme, mitochondrial | 0.11 | 0.079776 |
| Q9QXS1 | Plectin | 0.10 | 0.079882 |
| Q02053 | Ubiquitin-like modifier-activating enzyme 1 | 0.13 | 0.080017 |
| P63024 | Vesicle-associated membrane protein 3 | -0.13 | 0.08033 |
| O35143 | ATPase inhibitor, mitochondrial | -0.20 | 0.08039 |
| P63073 | Eukaryotic translation initiation factor 4E | -0.35 | 0.080597 |
| Q80UY2 | E3 ubiquitin-protein ligase KCMF1 | -0.24 | 0.080773 |
| Q5U430 | E3 ubiquitin-protein ligase UBR3 | 0.38 | 0.081462 |
| Q6P5F9 | Exportin-1 | 0.38 | 0.081761 |
| P56399 | Ubiquitin carboxyl-terminal hydrolase 5 | 0.18 | 0.081813 |
| Q6Y5D8 | Rho GTPase-activating protein 10 | 0.41 | 0.081824 |
| Q80SU7 | Interferon-induced very large GTPase 1 | 0.14 | 0.081925 |
| Q60772 | Cyclin-dependent kinase 4 inhibitor C | 0.57 | 0.082163 |
| Q924X2 | Carnitine O-palmitoyltransferase 1, muscle isoform | 0.09 | 0.082281 |
| P52196 | Thiosulfate sulfurtransferase | 0.18 | 0.082374 |
| P12382 | ATP-dependent 6-phosphofructokinase, liver type | 0.32 | 0.082474 |
| Q9CXJ4 | ATP-binding cassette sub-family B member 8, mitochondrial | 0.16 | 0.082524 |
| Q9D172 | ES1 protein homolog, mitochondrial | 0.27 | 0.082526 |
| Q04519 | Sphingomyelin phosphodiesterase | 0.37 | 0.082569 |
| P62717 | 60S ribosomal protein L18a | -0.32 | 0.082805 |
| Q9DC71 | 28S ribosomal protein S15, mitochondrial | 0.13 | 0.082814 |
| P60897 | 26S proteasome complex subunit DSS1 | 0.26 | 0.082961 |
| P70663 | SPARC-like protein 1 | 0.16 | 0.083195 |
| Q9WTQ8 | Mitochondrial import inner membrane translocase subunit Tim23 | -0.20 | 0.083251 |
| O08740 | DNA-directed RNA polymerase II subunit RPB11 | 0.29 | 0.083368 |
| Q62188 | Dihydropyrimidinase-related protein 3 | 0.32 | 0.083573 |
| Q05186 | Reticulocalbin-1 | -0.24 | 0.083802 |
| Q9JLV1 | BAG family molecular chaperone regulator 3 | -0.29 | 0.083811 |
| P28659 | CUGBP Elav-like family member 1 | 0.23 | 0.083835 |
| Q91V76 | Ester hydrolase C11orf54 homolog | -0.36 | 0.084032 |
| O55028 | [3-methyl-2-oxobutanoate dehydrogenase [lipoamide]] kinase, mitochondrial | 0.12 | 0.084284 |
| P54726 | UV excision repair protein RAD23 homolog A | 0.21 | 0.084473 |
| Q8VDJ3 | Vigilin | 0.12 | 0.084595 |
| Q91W89 | Alpha-mannosidase 2C1 | 0.31 | 0.084754 |
| P61358 | 60S ribosomal protein L27 | 0.19 | 0.084799 |
| P97290 | Plasma protease C1 inhibitor | 0.13 | 0.084885 |

| | | | |
|---|---|---|---|
| Q7TPR4 | Alpha-actinin-1 | -0.15 | 0.085022 |
| Q9CWE0 | Mitochondrial fission regulator 1-like | -0.13 | 0.085133 |
| Q6GSS7 | Histone H2A type 2-A;Histone H2A type 2-C | -0.22 | 0.085478 |
| Q9JKV1 | Proteasomal ubiquitin receptor ADRM1 | -0.26 | 0.085694 |
| P62774 | Myotrophin | 0.65 | 0.085724 |
| P23116 | Eukaryotic translation initiation factor 3 subunit A | 0.16 | 0.085876 |
| P61979 | Heterogeneous nuclear ribonucleoprotein K | -0.28 | 0.086222 |
| Q9DCH4 | Eukaryotic translation initiation factor 3 subunit F | -0.20 | 0.086289 |
| P25444 | 40S ribosomal protein S2 | 0.20 | 0.08641 |
| Q3TJZ6 | Protein FAM98A | -0.29 | 0.086654 |
| Q60931 | Voltage-dependent anion-selective channel protein 3 | -0.15 | 0.086712 |
| O08529 | Calpain-2 catalytic subunit | 0.11 | 0.086724 |
| Q8BVU5 | ADP-ribose pyrophosphatase, mitochondrial | -0.12 | 0.086903 |
| P63005 | Platelet-activating factor acetylhydrolase IB subunit alpha | -0.27 | 0.087181 |
| O09174 | Alpha-methylacyl-CoA racemase | 0.15 | 0.087327 |
| Q6ZWX6 | Eukaryotic translation initiation factor 2 subunit 1 | 0.45 | 0.087477 |
| P61014 | Cardiac phospholamban | 0.13 | 0.087841 |
| Q9QZ23 | NFU1 iron-sulfur cluster scaffold homolog, mitochondrial | 0.28 | 0.087883 |
| P18242 | Cathepsin D | 0.15 | 0.087916 |
| P09671 | Superoxide dismutase [Mn], mitochondrial | -0.30 | 0.08817 |
| Q8CHH9 | Septin-8 | 0.33 | 0.088273 |
| P24369 | Peptidyl-prolyl cis-trans isomerase B | -0.12 | 0.088385 |
| Q69Z99 | Zinc finger protein 512 | 0.20 | 0.088648 |
| P01837 | Ig kappa chain C region | -0.36 | 0.088788 |
| Q9EQJ0 | Two pore calcium channel protein 1 | 0.25 | 0.088909 |
| Q8VCE6 | 5(3)-deoxyribonucleotidase, mitochondrial | 0.14 | 0.08891 |
| Q8BMP6 | Golgi resident protein GCP60 | 0.27 | 0.088984 |
| Q9D404 | 3-oxoacyl-[acyl-carrier-protein] synthase, mitochondrial | 0.19 | 0.08907 |
| Q9JII5 | DAZ-associated protein 1 | -0.84 | 0.089084 |
| Q9JJF9 | Signal peptide peptidase-like 2A | -0.26 | 0.089137 |
| Q8BXZ1 | Protein disulfide-isomerase TMX3 | 0.30 | 0.089217 |
| Q9ERP3 | Tripartite motif-containing protein 54 | 0.46 | 0.089251 |
| P97855 | Ras GTPase-activating protein-binding protein 1 | 0.23 | 0.089408 |
| P62737 | Actin, aortic smooth muscle;Actin, gamma-enteric smooth muscle | -0.16 | 0.090176 |
| Q9CQV7 | Mitochondrial import inner membrane translocase subunit TIM14 | 0.18 | 0.090248 |
| P61255 | 60S ribosomal protein L26 | 0.26 | 0.090326 |
| Q8K3W2 | Leucine-rich repeat-containing protein 10 | -0.21 | 0.090353 |
| Q9JMA1 | Ubiquitin carboxyl-terminal hydrolase 14 | -0.14 | 0.090593 |
| P62843 | 40S ribosomal protein S15 | 0.11 | 0.091573 |

| | | | |
|---|---|---|---|
| Q9Z1Z2 | Serine-threonine kinase receptor-associated protein | 0.17 | 0.091702 |
| Q8K3H0 | DCC-interacting protein 13-alpha | 0.18 | 0.091736 |
| P00397 | Cytochrome c oxidase subunit 1 | 0.18 | 0.091954 |
| Q61107 | Guanylate-binding protein 4 | 0.52 | 0.092126 |
| Q9DBZ5 | Eukaryotic translation initiation factor 3 subunit K | 0.50 | 0.092463 |
| P98192 | Dihydroxyacetone phosphate acyltransferase | 0.58 | 0.092569 |
| Q5XG73 | Acyl-CoA-binding domain-containing protein 5 | 0.22 | 0.092614 |
| Q9D8P4 | 39S ribosomal protein L17, mitochondrial | -0.26 | 0.092958 |
| P52480 | Pyruvate kinase PKM | 0.17 | 0.092988 |
| Q9DBM2 | Peroxisomal bifunctional enzyme;Enoyl-CoA hydratase/3,2-trans-enoyl-CoA isomerase;3-hydroxyacyl-CoA dehydrogenase | 0.43 | 0.093012 |
| Q9Z2A5 | Arginyl-tRNA--protein transferase 1 | -0.22 | 0.093538 |
| P36552 | Oxygen-dependent coproporphyrinogen-III oxidase, mitochondrial | 0.13 | 0.093562 |
| P51859 | Hepatoma-derived growth factor | 0.13 | 0.093599 |
| Q8R035 | Peptidyl-tRNA hydrolase ICT1, mitochondrial | 0.19 | 0.093678 |
| P10639 | Thioredoxin | -0.18 | 0.093694 |
| Q03249 | Galactose-1-phosphate uridylyltransferase | 0.31 | 0.093857 |
| P85094 | Isochorismatase domain-containing protein 2A, mitochondrial | 0.20 | 0.09391 |
| P63321 | Ras-related protein Ral-A | -0.46 | 0.09434 |
| Q3UN90 | LYR motif-containing protein 9 | 0.17 | 0.09441 |
| P53994 | Ras-related protein Rab-2A | 0.22 | 0.094709 |
| P24527 | Leukotriene A-4 hydrolase | 0.11 | 0.094787 |
| Q9CQM5 | Thioredoxin domain-containing protein 17 | -0.18 | 0.094855 |
| Q8VEL0 | Motile sperm domain-containing protein 1 | -0.36 | 0.094979 |
| Q9Z1Z0 | General vesicular transport factor p115 | 0.11 | 0.095308 |
| Q922R8 | Protein disulfide-isomerase A6 | 0.10 | 0.095335 |
| P55096 | ATP-binding cassette sub-family D member 3 | 0.25 | 0.095409 |
| Q8CBE3 | WD repeat-containing protein 37 | 0.23 | 0.09553 |
| Q9WVL3 | Solute carrier family 12 member 7 | -0.11 | 0.095533 |
| Q6W8Q3 | Purkinje cell protein 4-like protein 1 | -0.47 | 0.095616 |
| Q921I1 | Serotransferrin | -0.10 | 0.095885 |
| P52760 | Ribonuclease UK114 | 0.21 | 0.095958 |
| Q7TNV0 | Protein DEK | 0.26 | 0.096747 |
| Q9DB05 | Alpha-soluble NSF attachment protein | -0.21 | 0.097098 |
| Q9JKF1 | Ras GTPase-activating-like protein IQGAP1 | 0.30 | 0.097255 |
| Q8JZN5 | Acyl-CoA dehydrogenase family member 9, mitochondrial | 0.13 | 0.097436 |
| Q9DC69 | NADH dehydrogenase [ubiquinone] 1 alpha subcomplex subunit 9, mitochondrial | 0.16 | 0.097489 |
| Q60737 | Casein kinase II subunit alpha | 0.25 | 0.097565 |

| | | | |
|---|---|---|---|
| Q9EPX2 | Papilin | 0.38 | 0.097789 |
| Q8BFP9 | [Pyruvate dehydrogenase (acetyl-transferring)] kinase isozyme 1, mitochondrial | 0.21 | 0.097923 |
| P47968 | Ribose-5-phosphate isomerase | 0.26 | 0.098525 |
| Q9CQC9 | GTP-binding protein SAR1b | -0.28 | 0.098888 |
| P40124 | Adenylyl cyclase-associated protein 1 | -0.20 | 0.098912 |
| Q8VIJ6 | Splicing factor, proline- and glutamine-rich | -0.10 | 0.098952 |
| Q6PA06 | Atlastin-2 | 0.09 | 0.099149 |
| Q9JLH8 | Tropomodulin-4 | 0.19 | 0.099203 |
| P14873 | Microtubule-associated protein 1B;MAP1B heavy chain;MAP1 light chain LC1 | 0.16 | 0.099641 |
| Q8VEE1 | LIM and cysteine-rich domains protein 1 | -0.15 | 0.100132 |
| P52825 | Carnitine O-palmitoyltransferase 2, mitochondrial | 0.12 | 0.100145 |
| Q9JKR6 | Hypoxia up-regulated protein 1 | 0.11 | 0.10018 |
| Q6PEB6 | MOB-like protein phocein | 0.25 | 0.100554 |
| Q64475 | Histone H2B type 1-B | 0.51 | 0.100556 |
| Q9D0R8 | Protein LSM12 homolog | 0.21 | 0.100805 |
| Q9WUA3 | ATP-dependent 6-phosphofructokinase, platelet type | 0.22 | 0.101198 |
| P54823 | Probable ATP-dependent RNA helicase DDX6 | 0.19 | 0.101268 |
| Q9WUZ7 | SH3 domain-binding glutamic acid-rich protein | -0.48 | 0.101547 |
| O88456 | Calpain small subunit 1 | -0.17 | 0.101853 |
| Q9WVL0 | Maleylacetoacetate isomerase | -0.14 | 0.102693 |
| Q8VE22 | 28S ribosomal protein S23, mitochondrial | 0.12 | 0.102981 |
| P07214 | SPARC | 0.23 | 0.10299 |
| O35680 | 28S ribosomal protein S12, mitochondrial | 0.26 | 0.103026 |
| Q9R190 | Metastasis-associated protein MTA2 | 0.56 | 0.103146 |
| Q07076 | Annexin A7 | 0.12 | 0.103473 |
| P31725 | Protein S100-A9 | 0.23 | 0.103575 |
| Q9R233 | Tapasin | 0.29 | 0.103612 |
| Q8VD75 | Huntingtin-interacting protein 1 | 0.52 | 0.103732 |
| Q8QZS1 | 3-hydroxyisobutyryl-CoA hydrolase, mitochondrial | 0.12 | 0.103948 |
| Q9CQR2 | 40S ribosomal protein S21 | -0.20 | 0.10405 |
| Q8CHS7 | Dehydrogenase/reductase SDR family member 7C | -0.09 | 0.104316 |
| Q8BGB8 | Ubiquinone biosynthesis protein COQ4 homolog, mitochondrial | 0.17 | 0.1051 |
| P06797 | Cathepsin L1;Cathepsin L1 heavy chain;Cathepsin L1 light chain | 0.54 | 0.105382 |
| O35435 | Dihydroorotate dehydrogenase (quinone), mitochondrial | 0.54 | 0.105603 |
| Q9Z1F9 | SUMO-activating enzyme subunit 2 | 0.13 | 0.105688 |
| Q3U1J4 | DNA damage-binding protein 1 | 0.18 | 0.106318 |
| P63028 | Translationally-controlled tumor protein | 0.11 | 0.10662 |
| Q62189 | U1 small nuclear ribonucleoprotein A | 0.13 | 0.106767 |
| Q91VA6 | Polymerase delta-interacting protein 2 | -0.14 | 0.107698 |

| | | | |
|---|---|---|---|
| Q80UG5 | Septin-9 | -0.16 | 0.107804 |
| Q8BGD9 | Eukaryotic translation initiation factor 4B | -0.21 | 0.108085 |
| P63017 | Heat shock cognate 71 kDa protein | 0.11 | 0.108197 |
| Q8JZN7 | Mitochondrial Rho GTPase 2 | -0.29 | 0.108244 |
| Q91UZ5 | Inositol monophosphatase 2 | 0.30 | 0.108271 |
| Q63932 | Dual specificity mitogen-activated protein kinase kinase 2 | 0.09 | 0.108525 |
| O35658 | Complement component 1 Q subcomponent-binding protein, mitochondrial | 0.23 | 0.108616 |
| Q64516 | Glycerol kinase | 0.15 | 0.108874 |
| C0HKE9 | Histone H2A type 1-H;Histone H2A type 1-F;Histone H2A type 3;Histone H2A type 1-K | -0.25 | 0.108921 |
| P31938 | Dual specificity mitogen-activated protein kinase kinase 1 | -0.21 | 0.109086 |
| Q6P542 | ATP-binding cassette sub-family F member 1 | 0.26 | 0.10941 |
| Q62167 | ATP-dependent RNA helicase DDX3X | 0.34 | 0.110152 |
| Q80U58 | Pumilio homolog 2 | 0.41 | 0.110401 |
| Q9WU28 | Prefoldin subunit 5 | -0.30 | 0.110544 |
| Q9CX56 | 26S proteasome non-ATPase regulatory subunit 8 | -0.17 | 0.110788 |
| O89086 | RNA-binding protein 3 | 0.18 | 0.110903 |
| Q9EQN3 | TSC22 domain family protein 4 | 0.25 | 0.110921 |
| Q9ERI6 | Retinol dehydrogenase 14 | -0.15 | 0.111326 |
| Q60902 | Epidermal growth factor receptor substrate 15-like 1 | -0.27 | 0.111419 |
| Q78J03 | Methionine-R-sulfoxide reductase B2, mitochondrial | -0.15 | 0.111446 |
| Q9D8C4 | Interferon-induced 35 kDa protein homolog | 0.19 | 0.111546 |
| Q9QXL1 | Kinesin-like protein KIF21B | 0.77 | 0.111595 |
| O70423 | Membrane primary amine oxidase | 0.17 | 0.111624 |
| Q3TLP5 | Enoyl-CoA hydratase domain-containing protein 2, mitochondrial | -0.27 | 0.111857 |
| Q505D7 | Optic atrophy 3 protein homolog | -0.15 | 0.111969 |
| Q99MJ9 | ATP-dependent RNA helicase DDX50 | 0.21 | 0.112134 |
| Q9D2N4 | Dystrobrevin alpha | 0.21 | 0.112483 |
| Q9Z0P4 | Paralemmin-1 | 0.22 | 0.112652 |
| Q9CQ00 | Transmembrane protein 261 | -0.17 | 0.112972 |
| Q8VDK1 | Nitrilase homolog 1 | 0.12 | 0.113051 |
| Q9QZ06 | Toll-interacting protein | 0.26 | 0.113089 |
| Q99L45 | Eukaryotic translation initiation factor 2 subunit 2 | -0.15 | 0.113245 |
| Q9D6J6 | NADH dehydrogenase [ubiquinone] flavoprotein 2, mitochondrial | 0.15 | 0.11339 |
| P01670 | Ig kappa chain V-III region PC 6684;Ig kappa chain V-V regions;Ig kappa chain V-V region HP 91A3;Ig kappa chain V-V region HP 124E1;Ig kappa chain V-V region HP 123E6;Ig kappa chain V-V region HP 93G7;Ig | 0.31 | 0.113677 |

| | | | |
|---|---|---|---|
| | kappa chain V-V region HP R16.7;Ig kappa chain V-V region MOPC 173;Ig kappa chain V-III region PC 7210;Ig kappa chain V-III region 50S10.1;Ig kappa chain V-III region PC 2485/PC 4039;Ig kappa chain V-III region PC 7940;Ig kappa chain V-III region PC 7175;Ig kappa chain V-III region PC 7769;Ig kappa chain V-III region PC 6308;Ig kappa chain V-III region PC 7183;Ig kappa chain V-III region PC 7043;Ig kappa chain V-III region CBPC 101;Ig kappa chain V-III region PC 3741/TEPC 111;Ig kappa chain V-III region PC 2413;Ig kappa chain V-III region MOPC 70;Ig kappa chain V-III region PC 2880/PC 1229;Ig kappa chain V-III region TEPC 124;Ig kappa chain V-III region PC 7132 | | |
| Q8K183 | Pyridoxal kinase | 0.12 | 0.114148 |
| Q9CQF9 | Prenylcysteine oxidase | 0.30 | 0.114614 |
| P97328 | Ketohexokinase | -0.30 | 0.114631 |
| Q61733 | 28S ribosomal protein S31, mitochondrial | -0.22 | 0.115928 |
| P46460 | Vesicle-fusing ATPase | 0.25 | 0.116114 |
| Q9QYI5 | DnaJ homolog subfamily B member 2 | 0.38 | 0.116147 |
| Q64726 | Zinc-alpha-2-glycoprotein | 0.29 | 0.11626 |
| P40240 | CD9 antigen | 1.02 | 0.116263 |
| Q3V3R4 | Integrin alpha-1 | -0.28 | 0.116623 |
| P50580 | Proliferation-associated protein 2G4 | 0.10 | 0.116811 |
| Q9CQM9 | Glutaredoxin-3 | 0.12 | 0.116848 |
| Q9D7G0 | Ribose-phosphate pyrophosphokinase 1;Ribose-phosphate pyrophosphokinase 2 | -0.11 | 0.116892 |
| Q80UZ0 | FYVE, RhoGEF and PH domain-containing protein 5 | 0.79 | 0.117066 |
| Q9CQT1 | Methylthioribose-1-phosphate isomerase | -0.19 | 0.117531 |
| P62192 | 26S protease regulatory subunit 4 | 0.09 | 0.117639 |
| Q9DBG7 | Signal recognition particle receptor subunit alpha | -0.18 | 0.118056 |
| Q8BK03 | Protein FAM73B | -0.15 | 0.118287 |
| Q99LB2 | Dehydrogenase/reductase SDR family member 4 | -0.16 | 0.118805 |
| O88544 | COP9 signalosome complex subunit 4 | 0.10 | 0.119123 |
| Q9D7D7 | Claudin-23 | 0.79 | 0.119235 |
| Q921T2 | Torsin-1A-interacting protein 1 | -0.29 | 0.119483 |
| Q60692 | Proteasome subunit beta type-6 | -0.16 | 0.119646 |
| Q8VE37 | Regulator of chromosome condensation | 0.09 | 0.119757 |
| P55937 | Golgin subfamily A member 3 | 0.36 | 0.119898 |
| Q9WVT6 | Carbonic anhydrase 14 | -0.15 | 0.11994 |
| Q8BJU0 | Small glutamine-rich tetratricopeptide repeat-containing protein alpha | 0.44 | 0.119951 |
| Q78IK4 | MICOS complex subunit Mic27 | 0.13 | 0.120166 |
| Q9CZY2 | Transcription elongation factor A protein-like 8 | 0.19 | 0.120537 |
| Q9CQJ6 | Density-regulated protein | -0.34 | 0.12063 |

| | | | |
|---|---|---|---|
| Q8VC19 | 5-aminolevulinate synthase, nonspecific, mitochondrial | -0.18 | 0.120814 |
| Q91YX5 | Acyl-CoA:lysophosphatidylglycerol acyltransferase 1 | -0.25 | 0.121258 |
| A2ASS6 | Titin | 0.11 | 0.121264 |
| P31786 | Acyl-CoA-binding protein | -0.18 | 0.12153 |
| Q5FWH6 | Rho guanine nucleotide exchange factor 15 | 0.17 | 0.121607 |
| Q9ES46 | Beta-parvin | 0.14 | 0.121691 |
| O55029 | Coatomer subunit beta | 0.17 | 0.122402 |
| P01029 | Complement C4-B;Complement C4 beta chain;Complement C4 alpha chain;C4a anaphylatoxin;Complement C4 gamma chain | 0.16 | 0.122602 |
| O09159 | Lysosomal alpha-mannosidase | 0.15 | 0.122928 |
| Q6RUT7 | Protein CCSMST1 | 0.15 | 0.123051 |
| Q2TPA8 | Hydroxysteroid dehydrogenase-like protein 2 | 0.14 | 0.12357 |
| Q3TAS6 | ER membrane protein complex subunit 10 | 0.54 | 0.123634 |
| O88487 | Cytoplasmic dynein 1 intermediate chain 2 | 0.35 | 0.123921 |
| O88712 | C-terminal-binding protein 1;C-terminal-binding protein 2 | 0.21 | 0.123942 |
| Q6QD59 | Vesicle transport protein SEC20 | 0.12 | 0.124313 |
| Q61595 | Kinectin | 0.10 | 0.124882 |
| Q8BJF9 | Charged multivesicular body protein 2b | -0.35 | 0.124992 |
| Q9WV02 | RNA-binding motif protein, X chromosome;RNA-binding motif protein, X chromosome, N-terminally processed | 0.18 | 0.125075 |
| P62900 | 60S ribosomal protein L31 | -0.22 | 0.125737 |
| Q60649 | Caseinolytic peptidase B protein homolog | 0.26 | 0.125796 |
| P84104 | Serine/arginine-rich splicing factor 3 | 0.13 | 0.126022 |
| Q9D7J9 | Enoyl-CoA hydratase domain-containing protein 3, mitochondrial | -0.16 | 0.12607 |
| Q6P5H6 | FERM domain-containing protein 5 | 0.18 | 0.12615 |
| Q3UHX2 | 28 kDa heat- and acid-stable phosphoprotein | 0.17 | 0.126166 |
| Q9QUI0 | Transforming protein RhoA | -0.15 | 0.126293 |
| Q8BFR5 | Elongation factor Tu, mitochondrial | -0.12 | 0.12631 |
| P01831 | Thy-1 membrane glycoprotein | 0.30 | 0.126657 |
| P29788 | Vitronectin | 0.20 | 0.126803 |
| Q06890 | Clusterin;Clusterin beta chain;Clusterin alpha chain | -0.11 | 0.127814 |
| Q8R5C5 | Beta-centractin | 0.32 | 0.128425 |
| Q8K1R7 | Serine/threonine-protein kinase Nek9 | 0.13 | 0.128468 |
| P23506 | Protein-L-isoaspartate(D-aspartate) O-methyltransferase | -0.17 | 0.128906 |
| Q9EP69 | Phosphatidylinositide phosphatase SAC1 | 0.09 | 0.12908 |
| O88322 | Nidogen-2 | 0.09 | 0.129459 |
| Q8BG95 | Protein phosphatase 1 regulatory subunit 12B | -0.09 | 0.129803 |
| Q9D855 | Cytochrome b-c1 complex subunit 7 | -0.14 | 0.129827 |
| Q61578 | NADPH:adrenodoxin oxidoreductase, mitochondrial | 0.10 | 0.130031 |

| | | | |
|---|---|---|---|
| P17225 | Polypyrimidine tract-binding protein 1 | 0.21 | 0.13014 |
| Q6H1V1 | Bestrophin-3 | 1.77 | 0.13057 |
| O88986 | 2-amino-3-ketobutyrate coenzyme A ligase, mitochondrial | 0.14 | 0.130581 |
| Q62136 | Tyrosine-protein phosphatase non-receptor type 21 | 0.34 | 0.130609 |
| Q8CDN6 | Thioredoxin-like protein 1 | 0.17 | 0.130642 |
| Q3V132 | ADP/ATP translocase 4;ADP/ATP translocase 4, N-terminally processed | -0.22 | 0.130741 |
| Q62419 | Endophilin-A2;Endophilin-A1 | 0.24 | 0.131301 |
| Q8BLF1 | Neutral cholesterol ester hydrolase 1 | 0.20 | 0.131382 |
| O35683 | NADH dehydrogenase [ubiquinone] 1 alpha subcomplex subunit 1 | 0.65 | 0.131531 |
| P17665 | Cytochrome c oxidase subunit 7C, mitochondrial | -0.23 | 0.131808 |
| Q8BYL4 | Tyrosine--tRNA ligase, mitochondrial | -0.14 | 0.131975 |
| P97300 | Neuroplastin | 0.26 | 0.132056 |
| Q9Z2Q5 | 39S ribosomal protein L40, mitochondrial | 0.17 | 0.132118 |
| Q922S4 | cGMP-dependent 3,5-cyclic phosphodiesterase | 0.12 | 0.132358 |
| Q8R2K1 | Fucose mutarotase | -3.67 | 0.132417 |
| Q3U2A8 | Valine--tRNA ligase, mitochondrial | 0.19 | 0.132462 |
| Q9JMA2 | Queuine tRNA-ribosyltransferase | 0.41 | 0.132697 |
| Q38HM4 | E3 ubiquitin-protein ligase TRIM63 | 0.21 | 0.133111 |
| Q9Z1P6 | NADH dehydrogenase [ubiquinone] 1 alpha subcomplex subunit 7 | 0.09 | 0.13333 |
| Q8VCH8 | UBX domain-containing protein 4 | -0.27 | 0.133525 |
| Q8BND5 | Sulfhydryl oxidase 1 | 0.11 | 0.133539 |
| P18572 | Basigin | 1.39 | 0.133543 |
| Q64FW2 | All-trans-retinol 13,14-reductase | 0.19 | 0.133658 |
| P04370 | Myelin basic protein | 0.19 | 0.133956 |
| Q8CC21 | Tetratricopeptide repeat protein 19, mitochondrial | -0.15 | 0.134007 |
| P47199 | Quinone oxidoreductase | 0.13 | 0.134007 |
| Q9CXR1 | Dehydrogenase/reductase SDR family member 7 | 0.12 | 0.134133 |
| P40142 | Transketolase | 0.11 | 0.13485 |
| Q64737 | Trifunctional purine biosynthetic protein adenosine-3;Phosphoribosylamine--glycine ligase;Phosphoribosylformylglycinamidine cyclo-ligase;Phosphoribosylglycinamide formyltransferase | 0.10 | 0.134908 |
| Q9JIW9 | Ras-related protein Ral-B | 0.10 | 0.134949 |
| P03987 | Ig gamma-3 chain C region | 0.24 | 0.135372 |
| Q9D110 | 5-formyltetrahydrofolate cyclo-ligase | 0.33 | 0.135467 |
| P01868 | Ig gamma-1 chain C region secreted form;Ig gamma-1 chain C region, membrane-bound form | 0.16 | 0.1355 |
| P38060 | Hydroxymethylglutaryl-CoA lyase, mitochondrial | -0.15 | 0.136545 |
| Q9JKB3 | Y-box-binding protein 3 | -0.24 | 0.136568 |
| Q64521 | Glycerol-3-phosphate dehydrogenase, mitochondrial | 0.14 | 0.136626 |

| | | | |
|---|---|---|---|
| P49817 | Caveolin-1 | -0.22 | 0.136956 |
| O88738 | Baculoviral IAP repeat-containing protein 6 | 0.18 | 0.137112 |
| Q9CQH3 | NADH dehydrogenase [ubiquinone] 1 beta subcomplex subunit 5, mitochondrial | 0.19 | 0.137114 |
| P20444 | Protein kinase C alpha type | -0.21 | 0.137246 |
| E9PV24 | Fibrinogen alpha chain;Fibrinopeptide A;Fibrinogen alpha chain | 0.14 | 0.137485 |
| Q9D0F3 | Protein ERGIC-53 | 0.13 | 0.138026 |
| Q8C0E2 | Vacuolar protein sorting-associated protein 26B | 0.60 | 0.138117 |
| P26645 | Myristoylated alanine-rich C-kinase substrate | -0.27 | 0.13842 |
| Q99P72 | Reticulon-4 | -0.16 | 0.138597 |
| Q62059 | Versican core protein | 0.15 | 0.139614 |
| Q6URW6 | Myosin-14 | 0.08 | 0.1397 |
| Q9ESD7 | Dysferlin | 0.09 | 0.139748 |
| Q9ERG2 | Striatin-3 | 0.13 | 0.139821 |
| A2AN08 | E3 ubiquitin-protein ligase UBR4 | -0.10 | 0.139976 |
| P42227 | Signal transducer and activator of transcription 3 | -0.09 | 0.140055 |
| P26350 | Prothymosin alpha;Prothymosin alpha, N-terminally processed;Thymosin alpha | -0.11 | 0.140464 |
| Q8BML9 | Glutamine--tRNA ligase | 0.15 | 0.140679 |
| Q8R1B5 | Complexin-3 | -0.23 | 0.140687 |
| P13541 | Myosin-3 | 0.09 | 0.141023 |
| P47857 | ATP-dependent 6-phosphofructokinase, muscle type | 0.16 | 0.141417 |
| P43883 | Perilipin-2 | 0.09 | 0.141442 |
| P21460 | Cystatin-C | 0.09 | 0.141691 |
| Q9DBN5 | Lon protease homolog 2, peroxisomal | 0.30 | 0.141715 |
| Q62465 | Synaptic vesicle membrane protein VAT-1 homolog | -0.24 | 0.141984 |
| P11930 | Nucleoside diphosphate-linked moiety X motif 19, mitochondrial | 0.16 | 0.142126 |
| Q3U0V1 | Far upstream element-binding protein 2 | -0.25 | 0.142199 |
| P32921 | Tryptophan--tRNA ligase, cytoplasmic;T1-TrpRS;T2-TrpRS | 0.17 | 0.142238 |
| Q8VDG5 | Phosphopantothenate--cysteine ligase | 0.10 | 0.142431 |
| Q9ESX5 | H/ACA ribonucleoprotein complex subunit 4 | 0.12 | 0.142755 |
| Q9WUR2 | Enoyl-CoA delta isomerase 2, mitochondrial | 0.10 | 0.14277 |
| P43277 | Histone H1.3 | 0.10 | 0.14284 |
| Q5DTX6 | Junctional protein associated with coronary artery disease | 0.13 | 0.143486 |
| Q6P9R2 | Serine/threonine-protein kinase OSR1 | -0.28 | 0.143622 |
| Q8BVZ1 | Perilipin-5 | 0.15 | 0.143862 |
| Q3UHB1 | 5-nucleotidase domain-containing protein 3 | -0.24 | 0.144055 |
| Q9WVE8 | Protein kinase C and casein kinase substrate in neurons protein 2 | 0.14 | 0.144157 |

| | | | |
|---|---|---|---|
| P32883 | GTPase KRas;GTPase KRas, N-terminally processed;GTPase NRas | 0.11 | 0.144452 |
| Q8R1Q8 | Cytoplasmic dynein 1 light intermediate chain 1 | 0.16 | 0.144645 |
| Q9QXG4 | Acetyl-coenzyme A synthetase, cytoplasmic | 0.44 | 0.145179 |
| P67984 | 60S ribosomal protein L22 | -0.15 | 0.145265 |
| Q9D1C3 | Protein preY, mitochondrial | -0.29 | 0.145913 |
| Q9D6R2 | Isocitrate dehydrogenase [NAD] subunit alpha, mitochondrial | 0.18 | 0.145988 |
| Q6PGL7 | WASH complex subunit FAM21 | 0.11 | 0.146719 |
| Q62393 | Tumor protein D52 | 0.12 | 0.147202 |
| Q80UP8 | Sodium-dependent phosphate transporter 2 | 0.15 | 0.14726 |
| Q921S7 | 39S ribosomal protein L37, mitochondrial | 0.17 | 0.147331 |
| Q68FH0 | Plakophilin-4 | 0.32 | 0.147895 |
| Q9QYB5 | Gamma-adducin | 0.37 | 0.147962 |
| Q9CPX6 | Ubiquitin-like-conjugating enzyme ATG3 | 0.14 | 0.148047 |
| Q9D0B5 | Thiosulfate sulfurtransferase/rhodanese-like domain-containing protein 3 | 0.32 | 0.148127 |
| Q8BTJ4 | Bis(5-adenosyl)-triphosphatase enpp4 | 0.29 | 0.148152 |
| O35857 | Mitochondrial import inner membrane translocase subunit TIM44 | 0.68 | 0.148362 |
| Q8BG51 | Mitochondrial Rho GTPase 1 | 0.10 | 0.148605 |
| P62305 | Small nuclear ribonucleoprotein E | 0.20 | 0.148673 |
| Q9R0M4 | Podocalyxin | -0.17 | 0.148728 |
| P14824 | Annexin A6 | 0.11 | 0.148744 |
| Q61268 | Apolipoprotein C-IV | 0.30 | 0.149198 |
| Q9R059 | Four and a half LIM domains protein 3 | -0.29 | 0.149456 |
| Q8CHK3 | Lysophospholipid acyltransferase 7 | -0.31 | 0.150126 |
| Q9DCM0 | Persulfide dioxygenase ETHE1, mitochondrial | 0.14 | 0.150147 |
| Q06138 | Calcium-binding protein 39 | -0.09 | 0.150362 |
| P50544 | Very long-chain specific acyl-CoA dehydrogenase, mitochondrial | 0.17 | 0.150899 |
| P99027 | 60S acidic ribosomal protein P2 | -0.17 | 0.151328 |
| P05622 | Platelet-derived growth factor receptor beta | 0.15 | 0.151359 |
| Q9D2G2 | Dihydrolipoyllysine-residue succinyltransferase component of 2-oxoglutarate dehydrogenase complex, mitochondrial | 0.09 | 0.151429 |
| P70404 | Isocitrate dehydrogenase [NAD] subunit gamma 1, mitochondrial | 0.08 | 0.152644 |
| Q99LI8 | Hepatocyte growth factor-regulated tyrosine kinase substrate | -0.14 | 0.15378 |
| Q3TC33 | Coiled-coil domain-containing protein 127 | 0.16 | 0.153899 |
| Q80X73 | Protein pelota homolog | 0.11 | 0.15391 |
| Q80YD1 | ATP-dependent RNA helicase SUPV3L1, mitochondrial | -0.15 | 0.154533 |

| | | | |
|---|---|---|---|
| P19221 | Prothrombin;Activation peptide fragment 1;Activation peptide fragment 2;Thrombin light chain;Thrombin heavy chain | -0.14 | 0.154852 |
| Q8BP92 | Reticulocalbin-2 | 0.16 | 0.154911 |
| Q9JHK4 | Geranylgeranyl transferase type-2 subunit alpha | 0.29 | 0.155033 |
| Q8BMZ5 | tRNA-splicing endonuclease subunit Sen34 | 0.40 | 0.155227 |
| P04919 | Band 3 anion transport protein | 0.23 | 0.155417 |
| Q80YQ8 | Protein RMD5 homolog A;Protein RMD5 homolog B | 0.22 | 0.1558 |
| P56959 | RNA-binding protein FUS | -0.21 | 0.155929 |
| Q8BR92 | Paralemmin-2 | 0.44 | 0.156189 |
| Q80ZS3 | 28S ribosomal protein S26, mitochondrial | 0.16 | 0.156353 |
| Q5XKN4 | Protein jagunal homolog 1 | 0.26 | 0.15679 |
| O88447 | Kinesin light chain 1 | -0.16 | 0.157224 |
| P42232 | Signal transducer and activator of transcription 5B | 0.15 | 0.158495 |
| Q9CQ80 | Vacuolar protein-sorting-associated protein 25 | 0.19 | 0.158641 |
| Q8BWM0 | Prostaglandin E synthase 2;Prostaglandin E synthase 2 truncated form | 0.19 | 0.158722 |
| Q8BX10 | Serine/threonine-protein phosphatase PGAM5, mitochondrial | 0.11 | 0.158961 |
| Q9QZQ8 | Core histone macro-H2A.1 | 0.79 | 0.159593 |
| P49312 | Heterogeneous nuclear ribonucleoprotein A1;Heterogeneous nuclear ribonucleoprotein A1, N-terminally processed | 0.10 | 0.160019 |
| A2AMM0 | Muscle-related coiled-coil protein | 0.11 | 0.160224 |
| O55131 | Septin-7 | -0.13 | 0.160265 |
| P19324 | Serpin H1 | 0.10 | 0.161592 |
| Q61102 | ATP-binding cassette sub-family B member 7, mitochondrial | -0.13 | 0.161642 |
| Q8R2Z5 | von Willebrand factor A domain-containing protein 1 | 0.14 | 0.16238 |
| O35887 | Calumenin | -0.19 | 0.162493 |
| O08759 | Ubiquitin-protein ligase E3A | -0.15 | 0.162618 |
| Q8JZV7 | Putative N-acetylglucosamine-6-phosphate deacetylase | 0.27 | 0.162884 |
| P02088 | Hemoglobin subunit beta-1;Hemoglobin subunit beta-2 | 0.15 | 0.163294 |
| Q80XN0 | D-beta-hydroxybutyrate dehydrogenase, mitochondrial | -0.17 | 0.163484 |
| Q99MR6 | Serrate RNA effector molecule homolog | 0.09 | 0.163896 |
| P26041 | Moesin | -0.17 | 0.163953 |
| Q3U9G9 | Lamin-B receptor | 0.15 | 0.16414 |
| P35279 | Ras-related protein Rab-6A | 0.12 | 0.164183 |
| Q99L47 | Hsc70-interacting protein | -0.26 | 0.164265 |
| Q9D8N0 | Elongation factor 1-gamma | 0.16 | 0.164727 |
| Q9JIK9 | 28S ribosomal protein S34, mitochondrial | 0.20 | 0.164787 |
| Q06185 | ATP synthase subunit e, mitochondrial | 0.09 | 0.16486 |
| Q3B7Z2 | Oxysterol-binding protein 1 | 0.11 | 0.164966 |

| | | | |
|---|---|---|---|
| Q8C3S2 | Transport and Golgi organization protein 6 homolog | -0.75 | 0.16503 |
| Q9ER88 | 28S ribosomal protein S29, mitochondrial | -0.09 | 0.165039 |
| Q64518 | Sarcoplasmic/endoplasmic reticulum calcium ATPase 3 | -0.28 | 0.165345 |
| Q9CXA2 | Trans-L-3-hydroxyproline dehydratase | 0.13 | 0.165692 |
| P60335 | Poly(rC)-binding protein 1 | 0.09 | 0.165972 |
| Q61011 | Guanine nucleotide-binding protein G(I)/G(S)/G(T) subunit beta-3 | 0.22 | 0.166018 |
| P97478 | 5-demethoxyubiquinone hydroxylase, mitochondrial | 0.16 | 0.166185 |
| Q5BKP2 | Ubiquitin carboxyl-terminal hydrolase 13 | 0.37 | 0.166376 |
| Q99NB8 | Ubiquilin-4 | 0.37 | 0.167001 |
| Q923Z3 | Protein MTO1 homolog, mitochondrial | -0.27 | 0.167153 |
| A2ASQ1 | Agrin;Agrin N-terminal 110 kDa subunit;Agrin C-terminal 110 kDa subunit;Agrin C-terminal 90 kDa fragment;Agrin C-terminal 22 kDa fragment | 0.16 | 0.167875 |
| Q9D832 | DnaJ homolog subfamily B member 4 | 0.18 | 0.168476 |
| Q9CYN2 | Signal peptidase complex subunit 2 | 0.17 | 0.168532 |
| O89053 | Coronin-1A | 0.10 | 0.168568 |
| Q62418 | Drebrin-like protein | 0.17 | 0.168603 |
| P80318 | T-complex protein 1 subunit gamma | 0.07 | 0.168612 |
| P15116 | Cadherin-2 | -0.10 | 0.169374 |
| Q9QWV4 | Myeloid leukemia factor 1 | -0.15 | 0.169699 |
| Q9CZP5 | Mitochondrial chaperone BCS1 | 0.09 | 0.169806 |
| Q3THF9 | Coenzyme Q-binding protein COQ10 homolog B, mitochondrial | -0.25 | 0.170304 |
| Q9CXT8 | Mitochondrial-processing peptidase subunit beta | 0.07 | 0.170728 |
| P02469 | Laminin subunit beta-1 | 0.10 | 0.170846 |
| Q64735 | Complement component receptor 1-like protein | 0.24 | 0.171136 |
| Q8BV13 | COP9 signalosome complex subunit 7b | -0.16 | 0.171184 |
| Q9JIQ3 | Diablo homolog, mitochondrial | -0.12 | 0.171332 |
| Q9WVQ5 | Methylthioribulose-1-phosphate dehydratase | 0.09 | 0.171496 |
| Q9CQ45 | Neudesin | 0.14 | 0.171717 |
| P14094 | Sodium/potassium-transporting ATPase subunit beta-1 | -0.20 | 0.172249 |
| Q8R404 | Protein QIL1 | 0.08 | 0.172478 |
| Q9CR39 | WD repeat domain phosphoinositide-interacting protein 3 | 0.73 | 0.173347 |
| P62492 | Ras-related protein Rab-11A;Ras-related protein Rab-11B | -0.12 | 0.173483 |
| O88545 | COP9 signalosome complex subunit 6 | 0.12 | 0.173702 |
| Q3UQ28 | Peroxidasin homolog | 0.12 | 0.173928 |
| Q14C51 | Pentatricopeptide repeat domain-containing protein 3, mitochondrial | 0.21 | 0.174093 |
| Q9QXA5 | U6 snRNA-associated Sm-like protein LSm4 | 0.17 | 0.17413 |
| Q9R1P0 | Proteasome subunit alpha type-4 | -0.18 | 0.174377 |
| P17710 | Hexokinase-1 | 0.07 | 0.174499 |

| | | | |
|---|---|---|---|
| Q9D0R2 | Threonine--tRNA ligase, cytoplasmic | -0.21 | 0.174554 |
| E9QAT4 | Protein transport protein Sec16A | -0.26 | 0.174924 |
| P56213 | FAD-linked sulfhydryl oxidase ALR | 0.27 | 0.175005 |
| P97807 | Fumarate hydratase, mitochondrial | 0.10 | 0.175364 |
| P35762 | CD81 antigen | -0.10 | 0.175402 |
| P12787 | Cytochrome c oxidase subunit 5A, mitochondrial | 0.12 | 0.175403 |
| Q62159 | Rho-related GTP-binding protein RhoC | 0.55 | 0.175514 |
| Q9CXD6 | Mitochondrial calcium uniporter regulator 1 | -0.17 | 0.175813 |
| Q8R2Y8 | Peptidyl-tRNA hydrolase 2, mitochondrial | 0.21 | 0.175939 |
| Q9WTX5 | S-phase kinase-associated protein 1 | -0.21 | 0.176301 |
| Q08857 | Platelet glycoprotein 4 | -0.23 | 0.176322 |
| Q9JJW5 | Myozenin-2 | 0.10 | 0.176477 |
| Q62261 | Spectrin beta chain, non-erythrocytic 1 | 0.10 | 0.176531 |
| Q64012 | RNA-binding protein Raly | -0.11 | 0.176627 |
| Q9ER00 | Syntaxin-12 | 0.14 | 0.177295 |
| Q922F4 | Tubulin beta-6 chain | 0.16 | 0.177349 |
| Q9DBH5 | Vesicular integral-membrane protein VIP36 | -0.17 | 0.177822 |
| Q9QZ73 | DCN1-like protein 1;DCN1-like protein 2 | -0.10 | 0.178085 |
| Q9CRC3 | UPF0235 protein C15orf40 homolog | -0.16 | 0.178807 |
| Q9EPJ9 | ADP-ribosylation factor GTPase-activating protein 1 | 0.87 | 0.180164 |
| Q9CPQ1 | Cytochrome c oxidase subunit 6C | -0.21 | 0.180583 |
| Q8BH58 | TIP41-like protein | 0.23 | 0.180935 |
| Q60790 | Ras GTPase-activating protein 3 | 0.19 | 0.181467 |
| O08738 | Caspase-6;Caspase-6 subunit p18;Caspase-6 subunit p11 | 0.30 | 0.182028 |
| Q8BGT6 | MICAL-like protein 1 | 0.39 | 0.182344 |
| Q9Z2C5 | Myotubularin | 0.12 | 0.182617 |
| Q7TQ48 | Sarcalumenin | 0.08 | 0.182965 |
| Q7TMW6 | Cytosolic Fe-S cluster assembly factor NARFL | 0.13 | 0.18309 |
| P14142 | Solute carrier family 2, facilitated glucose transporter member 4 | 0.26 | 0.183341 |
| Q8R1G2 | Carboxymethylenebutenolidase homolog | -0.15 | 0.18357 |
| Q9JHU4 | Cytoplasmic dynein 1 heavy chain 1 | 0.07 | 0.18394 |
| P39749 | Flap endonuclease 1 | 0.27 | 0.183999 |
| Q9QXZ0 | Microtubule-actin cross-linking factor 1 | -0.07 | 0.184344 |
| Q9CY16 | 28S ribosomal protein S28, mitochondrial | -0.14 | 0.184497 |
| Q8BH04 | Phosphoenolpyruvate carboxykinase [GTP], mitochondrial | 0.22 | 0.184575 |
| P05202 | Aspartate aminotransferase, mitochondrial | -0.08 | 0.184831 |
| Q9CSU0 | Regulation of nuclear pre-mRNA domain-containing protein 1B | 0.11 | 0.184999 |
| O88990 | Alpha-actinin-3 | 0.30 | 0.185111 |
| A2AUC9 | Kelch-like protein 41 | 0.15 | 0.185569 |

| | | | |
|---|---|---|---|
| Q9ESL4 | Mitogen-activated protein kinase kinase kinase MLT | -0.13 | 0.186014 |
| P56392 | Cytochrome c oxidase subunit 7A1, mitochondrial | 0.15 | 0.186161 |
| Q8BMJ2 | Leucine--tRNA ligase, cytoplasmic | 0.13 | 0.186393 |
| Q3TVI8 | Pre-B-cell leukemia transcription factor-interacting protein 1 | 0.19 | 0.186498 |
| Q8C167 | Prolyl endopeptidase-like | -0.12 | 0.186924 |
| Q9CQW2 | ADP-ribosylation factor-like protein 8B | -0.25 | 0.187126 |
| P04186 | Complement factor B;Complement factor B Ba fragment;Complement factor B Bb fragment | 0.17 | 0.1872 |
| Q9CXW4 | 60S ribosomal protein L11 | -0.08 | 0.187298 |
| P23198 | Chromobox protein homolog 3 | 0.11 | 0.188552 |
| P22315 | Ferrochelatase, mitochondrial | 0.12 | 0.188736 |
| Q9R1Z7 | 6-pyruvoyl tetrahydrobiopterin synthase | 0.12 | 0.189504 |
| Q9CXW3 | Calcyclin-binding protein | -0.10 | 0.189531 |
| Q9CR62 | Mitochondrial 2-oxoglutarate/malate carrier protein | 0.09 | 0.190112 |
| Q6PGC1 | ATP-dependent RNA helicase Dhx29 | 0.29 | 0.190849 |
| Q9DBC7 | cAMP-dependent protein kinase type I-alpha regulatory subunit;cAMP-dependent protein kinase type I-alpha regulatory subunit, N-terminally processed | -0.11 | 0.191095 |
| Q6PE15 | Mycophenolic acid acyl-glucuronide esterase, mitochondrial | 0.11 | 0.191759 |
| Q9D0I9 | Arginine--tRNA ligase, cytoplasmic | 0.10 | 0.192154 |
| Q9D898 | Actin-related protein 2/3 complex subunit 5-like protein | 0.22 | 0.192226 |
| Q9R0L6 | Pericentriolar material 1 protein | -0.29 | 0.193159 |
| Q61990 | Poly(rC)-binding protein 2;Poly(rC)-binding protein 3 | -0.20 | 0.193448 |
| P97429 | Annexin A4 | -0.10 | 0.193516 |
| Q9CQE2 | Transmembrane protein 223 | 0.10 | 0.194527 |
| Q91WK5 | Glycine cleavage system H protein, mitochondrial | -0.13 | 0.19471 |
| P07901 | Heat shock protein HSP 90-alpha | 0.18 | 0.195091 |
| Q8BKX6 | Serine/threonine-protein kinase SMG1 | 0.50 | 0.195255 |
| Q8BH93 | MAPK-interacting and spindle-stabilizing protein-like | 0.09 | 0.195357 |
| Q99PR8 | Heat shock protein beta-2 | 0.24 | 0.195544 |
| O08528 | Hexokinase-2 | 0.07 | 0.197254 |
| P42932 | T-complex protein 1 subunit theta | 0.11 | 0.19737 |
| Q8CC86 | Nicotinate phosphoribosyltransferase | 0.14 | 0.197664 |
| Q9WTL7 | Acyl-protein thioesterase 2 | -0.12 | 0.197832 |
| P97863 | Nuclear factor 1 B-type;Nuclear factor 1 C-type;Nuclear factor 1 X-type | 0.20 | 0.19843 |
| Q61235 | Beta-2-syntrophin | 0.12 | 0.198439 |
| Q80UY1 | UPF0586 protein C9orf41 homolog | 0.18 | 0.198639 |
| P61971 | Nuclear transport factor 2 | 0.15 | 0.198803 |
| P84228 | Histone H3.2;Histone H3.1;Histone H3.3;Histone H3.3C | 0.19 | 0.198842 |
| P67778 | Prohibitin | 0.14 | 0.198964 |

| | | | |
|---|---|---|---|
| O08917 | Flotillin-1 | -0.10 | 0.199006 |
| Q61425 | Hydroxyacyl-coenzyme A dehydrogenase, mitochondrial | -0.16 | 0.199097 |
| Q9DBC0 | Selenoprotein O | 0.40 | 0.199102 |
| Q8VHY0 | Chondroitin sulfate proteoglycan 4 | 0.10 | 0.199256 |
| O35682 | Myeloid-associated differentiation marker | -0.19 | 0.199361 |
| Q9R111 | Guanine deaminase | 0.27 | 0.199473 |
| P97467 | Peptidyl-glycine alpha-amidating monooxygenase;Peptidylglycine alpha-hydroxylating monooxygenase;Peptidyl-alpha-hydroxyglycine alpha-amidating lyase | -0.16 | 0.199705 |
| O88492 | Perilipin-4 | -0.10 | 0.199828 |
| Q99P30 | Peroxisomal coenzyme A diphosphatase NUDT7 | -0.26 | 0.199987 |
| P51885 | Lumican | -0.08 | 0.200147 |
| Q9Z1P7 | KN motif and ankyrin repeat domain-containing protein 3 | -0.13 | 0.200518 |
| Q9CQC7 | NADH dehydrogenase [ubiquinone] 1 beta subcomplex subunit 4 | 0.22 | 0.201381 |
| P70195 | Proteasome subunit beta type-7 | 0.34 | 0.202197 |
| Q8K3J1 | NADH dehydrogenase [ubiquinone] iron-sulfur protein 8, mitochondrial | 0.08 | 0.202754 |
| Q9D8X2 | Coiled-coil domain-containing protein 124 | 0.08 | 0.202805 |
| Q9CQB2 | Protein FAM195A | -0.41 | 0.202966 |
| Q9CQ56 | Vesicle transport protein USE1 | 0.12 | 0.203159 |
| P02463 | Collagen alpha-1(IV) chain;Arresten | 0.22 | 0.204447 |
| O70325 | Phospholipid hydroperoxide glutathione peroxidase, mitochondrial | 0.17 | 0.204474 |
| Q8K182 | Complement component C8 alpha chain | -0.18 | 0.204835 |
| Q923D2 | Flavin reductase (NADPH) | -0.09 | 0.204977 |
| P50462 | Cysteine and glycine-rich protein 3 | 0.43 | 0.205166 |
| P0C605 | cGMP-dependent protein kinase 1 | 0.24 | 0.205333 |
| Q9D2V7 | Coronin-7 | -0.15 | 0.205871 |
| P30115 | Glutathione S-transferase A3;Glutathione S-transferase A1;Glutathione S-transferase A1, N-terminally processed | -0.16 | 0.206275 |
| P62880 | Guanine nucleotide-binding protein G(I)/G(S)/G(T) subunit beta-2 | 0.08 | 0.206281 |
| P26516 | 26S proteasome non-ATPase regulatory subunit 7 | -0.11 | 0.206631 |
| P51855 | Glutathione synthetase | 0.10 | 0.206806 |
| Q3TJD7 | PDZ and LIM domain protein 7 | -0.15 | 0.206981 |
| P35235 | Tyrosine-protein phosphatase non-receptor type 11 | -0.14 | 0.20701 |
| Q8VEK3 | Heterogeneous nuclear ribonucleoprotein U | -0.17 | 0.207115 |
| Q9WU84 | Copper chaperone for superoxide dismutase | 0.21 | 0.207139 |
| Q99LT0 | Protein dpy-30 homolog | -0.10 | 0.208065 |

| | | | |
|---|---|---|---|
| P07309 | Transthyretin | -0.14 | 0.208568 |
| P62908 | 40S ribosomal protein S3 | 0.12 | 0.209191 |
| Q8C8R3 | Ankyrin-2 | 0.15 | 0.209206 |
| A2AWA9 | Rab GTPase-activating protein 1 | -0.10 | 0.209323 |
| Q923K4 | tRNA modification GTPase GTPBP3, mitochondrial | 0.30 | 0.209559 |
| A3KFX0 | Cytosolic 5-nucleotidase 1A | 0.30 | 0.209994 |
| P04117 | Fatty acid-binding protein, adipocyte | 0.09 | 0.210282 |
| P70460 | Vasodilator-stimulated phosphoprotein | 0.28 | 0.2104 |
| Q8BJW6 | Eukaryotic translation initiation factor 2A;Eukaryotic translation initiation factor 2A, N-terminally processed | 0.40 | 0.210934 |
| Q9QYA2 | Mitochondrial import receptor subunit TOM40 homolog | 0.26 | 0.211403 |
| Q6PDG5 | SWI/SNF complex subunit SMARCC2 | 0.34 | 0.212057 |
| Q9WTZ1 | RING-box protein 2 | 0.17 | 0.21209 |
| P45591 | Cofilin-2 | 0.08 | 0.21319 |
| O09131 | Glutathione S-transferase omega-1 | -0.12 | 0.213391 |
| Q9CPW2 | Adrenodoxin-like protein, mitochondrial | 0.50 | 0.213514 |
| Q80TV8 | CLIP-associating protein 1 | -0.09 | 0.213872 |
| P43274 | Histone H1.4 | -0.17 | 0.214546 |
| P11499 | Heat shock protein HSP 90-beta | -0.08 | 0.214558 |
| P28474 | Alcohol dehydrogenase class-3 | 0.08 | 0.214679 |
| Q9R0Y5 | Adenylate kinase isoenzyme 1 | 0.13 | 0.214846 |
| P28653 | Biglycan | 0.12 | 0.215322 |
| P97742 | Carnitine O-palmitoyltransferase 1, liver isoform | 0.10 | 0.215473 |
| Q8VDM6 | Heterogeneous nuclear ribonucleoprotein U-like protein 1 | 0.19 | 0.216272 |
| Q9WTI7 | Unconventional myosin-Ic | 0.23 | 0.216709 |
| Q9JKS4 | LIM domain-binding protein 3 | 0.10 | 0.216949 |
| O88342 | WD repeat-containing protein 1 | -0.09 | 0.216949 |
| P11087 | Collagen alpha-1(I) chain | -0.33 | 0.217158 |
| Q91VM5 | RNA binding motif protein, X-linked-like-1 | 0.07 | 0.217176 |
| Q8BHC4 | Dephospho-CoA kinase domain-containing protein | 0.17 | 0.217769 |
| Q9CQ22 | Ragulator complex protein LAMTOR1 | 0.20 | 0.218164 |
| P26043 | Radixin | 0.19 | 0.218424 |
| P97447 | Four and a half LIM domains protein 1 | -0.12 | 0.2185 |
| P39053 | Dynamin-1 | 0.27 | 0.219525 |
| Q3TXS7 | 26S proteasome non-ATPase regulatory subunit 1 | -0.08 | 0.220278 |
| P06728 | Apolipoprotein A-IV | 0.08 | 0.22045 |
| Q68FL4 | Putative adenosylhomocysteinase 3;Putative adenosylhomocysteinase 2 | -0.12 | 0.22049 |
| P47740 | Fatty aldehyde dehydrogenase | 0.10 | 0.220662 |
| P61202 | COP9 signalosome complex subunit 2 | -0.11 | 0.221172 |
| O88587 | Catechol O-methyltransferase | 0.08 | 0.221421 |
| Q6NZJ6 | Eukaryotic translation initiation factor 4 gamma 1 | 0.12 | 0.221997 |

| | | | |
|---|---|---|---|
| O09061 | Proteasome subunit beta type-1 | 0.15 | 0.222708 |
| Q8BWG8 | Beta-arrestin-1 | 0.33 | 0.222751 |
| Q9D8Z2 | TP53-regulated inhibitor of apoptosis 1 | 0.13 | 0.224302 |
| Q64525 | Histone H2B type 2-B | 0.84 | 0.224358 |
| Q60722 | Transcription factor 4;Transcription factor 12 | 0.51 | 0.224522 |
| Q8C129 | Leucyl-cystinyl aminopeptidase | 0.11 | 0.224855 |
| Q9D7X8 | Gamma-glutamylcyclotransferase | -0.11 | 0.22517 |
| Q9JLB0 | MAGUK p55 subfamily member 6 | 0.12 | 0.225641 |
| Q9CY66 | H/ACA ribonucleoprotein complex subunit 1 | 0.27 | 0.225735 |
| Q9DAK9 | 14 kDa phosphohistidine phosphatase | -0.13 | 0.226004 |
| P24547 | Inosine-5-monophosphate dehydrogenase 2 | -0.18 | 0.22619 |
| Q9QUR6 | Prolyl endopeptidase | 0.16 | 0.226248 |
| P35585 | AP-1 complex subunit mu-1 | 0.33 | 0.226496 |
| O70209 | PDZ and LIM domain protein 3 | -0.12 | 0.226525 |
| P07758 | Alpha-1-antitrypsin 1-1 | -0.14 | 0.226565 |
| P17751 | Triosephosphate isomerase | -0.09 | 0.226749 |
| D3Z1D3 | Cardiac-enriched FHL2-interacting protein | 0.27 | 0.226765 |
| Q9D009 | Putative lipoyltransferase 2, mitochondrial | -0.34 | 0.227153 |
| Q9JJU8 | SH3 domain-binding glutamic acid-rich-like protein | 0.19 | 0.227194 |
| Q11136 | Xaa-Pro dipeptidase | 0.14 | 0.22912 |
| Q922Y1 | UBX domain-containing protein 1 | 0.08 | 0.229267 |
| Q3UMR5 | Calcium uniporter protein, mitochondrial | 0.09 | 0.229432 |
| Q04736 | Tyrosine-protein kinase Yes;Tyrosine-protein kinase Fyn | -0.11 | 0.229731 |
| Q61152 | Tyrosine-protein phosphatase non-receptor type 18 | -0.19 | 0.230181 |
| P50247 | Adenosylhomocysteinase | 0.13 | 0.231274 |
| O70194 | Eukaryotic translation initiation factor 3 subunit D | -0.17 | 0.231284 |
| P56376 | Acylphosphatase-1 | -0.07 | 0.231696 |
| P51150 | Ras-related protein Rab-7a | 0.30 | 0.232046 |
| Q6Q477 | Calcium-transporting ATPase | 0.10 | 0.232138 |
| P52430 | Serum paraoxonase/arylesterase 1 | -0.17 | 0.232832 |
| P47754 | F-actin-capping protein subunit alpha-2 | 0.07 | 0.232844 |
| P37040 | NADPH--cytochrome P450 reductase | -0.09 | 0.233155 |
| O35344 | Importin subunit alpha-4 | -0.15 | 0.233492 |
| O55186 | CD59A glycoprotein | 0.22 | 0.233758 |
| Q62446 | Peptidyl-prolyl cis-trans isomerase FKBP3 | 0.09 | 0.233961 |
| O35678 | Monoglyceride lipase | 0.15 | 0.234932 |
| A3KG59 | Peptidase M20 domain-containing protein 2 | 0.07 | 0.235036 |
| Q9D0M1 | Phosphoribosyl pyrophosphate synthase-associated protein 1 | 0.08 | 0.23519 |
| Q91VJ4 | Serine/threonine-protein kinase 38 | 0.15 | 0.235326 |
| Q924M7 | Mannose-6-phosphate isomerase | -0.07 | 0.236124 |

| | | | |
|---|---|---|---|
| Q9CQ54 | NADH dehydrogenase [ubiquinone] 1 subunit C2 | 0.10 | 0.23653 |
| Q9DCL8 | Protein phosphatase inhibitor 2 | -0.12 | 0.236896 |
| P47757 | F-actin-capping protein subunit beta | 0.11 | 0.237893 |
| P0DP28 | Calmodulin-like protein 3 | -0.19 | 0.237951 |
| Q9QZB9 | Dynactin subunit 5 | 0.40 | 0.23797 |
| Q9DCX2 | ATP synthase subunit d, mitochondrial | 0.25 | 0.238096 |
| Q8VD04 | GRIP1-associated protein 1 | 0.25 | 0.238293 |
| Q8R313 | Exocyst complex component 6 | 0.45 | 0.239188 |
| Q9QZ88 | Vacuolar protein sorting-associated protein 29 | -0.14 | 0.239512 |
| P60229 | Eukaryotic translation initiation factor 3 subunit E | -0.08 | 0.239513 |
| P62311 | U6 snRNA-associated Sm-like protein LSm3 | 0.13 | 0.23952 |
| Q9R1S8 | Calpain-7 | 0.15 | 0.239903 |
| P47791 | Glutathione reductase, mitochondrial | -0.08 | 0.240018 |
| Q9CPX8 | Cytochrome b-c1 complex subunit 10 | -0.32 | 0.240091 |
| Q91WP6 | Serine protease inhibitor A3N;Serine protease inhibitor A3G | -0.16 | 0.240269 |
| Q91VC9 | Growth hormone-inducible transmembrane protein | -0.22 | 0.240714 |
| Q99N92 | 39S ribosomal protein L27, mitochondrial | 0.17 | 0.240948 |
| O55143 | Sarcoplasmic/endoplasmic reticulum calcium ATPase 2 | 0.07 | 0.241019 |
| Q61879 | Myosin-10 | 0.09 | 0.241163 |
| Q3TMP8 | Trimeric intracellular cation channel type A | 0.43 | 0.241194 |
| Q9Z1D1 | Eukaryotic translation initiation factor 3 subunit G | 0.21 | 0.241291 |
| O88441 | Metaxin-2 | 0.18 | 0.241343 |
| E1U8D0 | Protein SOGA1;N-terminal form;C-terminal 80 kDa form | -0.34 | 0.241708 |
| Q9CY64 | Biliverdin reductase A | -0.17 | 0.242136 |
| Q9Z2U1 | Proteasome subunit alpha type-5 | 0.15 | 0.242309 |
| Q9CQX2 | Cytochrome b5 type B | 0.15 | 0.242362 |
| Q9D517 | 1-acyl-sn-glycerol-3-phosphate acyltransferase gamma | -0.11 | 0.242481 |
| O08677 | Kininogen-1;Kininogen-1 heavy chain;Bradykinin;Kininogen-1 light chain | -0.09 | 0.242913 |
| Q61584 | Fragile X mental retardation syndrome-related protein 1 | 0.16 | 0.243499 |
| Q02357 | Ankyrin-1 | 0.29 | 0.245275 |
| Q68FD5 | Clathrin heavy chain 1 | 0.12 | 0.245293 |
| Q8VDC1 | FYVE and coiled-coil domain-containing protein 1 | 0.09 | 0.245727 |
| Q62376 | U1 small nuclear ribonucleoprotein 70 kDa | -0.14 | 0.245741 |
| Q9ERS2 | NADH dehydrogenase [ubiquinone] 1 alpha subcomplex subunit 13 | 0.07 | 0.245765 |
| Q9EQK5 | Major vault protein | 0.09 | 0.245879 |
| P56135 | ATP synthase subunit f, mitochondrial | 0.08 | 0.246437 |
| Q61330 | Contactin-2 | 0.47 | 0.246547 |
| Q9DBB5 | Eukaryotic translation initiation factor 4E type 3 | 0.16 | 0.247068 |
| P83882 | 60S ribosomal protein L36a | 0.09 | 0.247645 |

| | | | |
|---|---|---|---|
| Q9CR10 | Oxidoreductase-like domain-containing protein 1 | 0.19 | 0.247753 |
| P70699 | Lysosomal alpha-glucosidase | 0.07 | 0.24777 |
| Q9DCL9 | Multifunctional protein ADE2;Phosphoribosylaminoimidazole-succinocarboxamide synthase;Phosphoribosylaminoimidazole carboxylase | 0.13 | 0.248874 |
| O35864 | COP9 signalosome complex subunit 5 | -0.24 | 0.250806 |
| Q1HFZ0 | tRNA (cytosine(34)-C(5))-methyltransferase | 0.13 | 0.250927 |
| Q9DB20 | ATP synthase subunit O, mitochondrial | 0.09 | 0.251371 |
| Q9DCN2 | NADH-cytochrome b5 reductase 3;NADH-cytochrome b5 reductase 3 membrane-bound form;NADH-cytochrome b5 reductase 3 soluble form | 0.15 | 0.25161 |
| O54988 | STE20-like serine/threonine-protein kinase | 0.07 | 0.251839 |
| Q7TSS2 | Ubiquitin-conjugating enzyme E2 Q1 | 0.23 | 0.253094 |
| Q8VEH8 | Endoplasmic reticulum lectin 1 | 0.25 | 0.254513 |
| Q1XH17 | Tripartite motif-containing protein 72 | 0.06 | 0.254653 |
| Q71FD7 | Filamin-binding LIM protein 1 | 0.06 | 0.254742 |
| Q9CQV5 | 28S ribosomal protein S24, mitochondrial | -0.10 | 0.254834 |
| P0DPB4 | Schwannomin-interacting protein 1 | -0.14 | 0.256059 |
| Q9DAS9 | Guanine nucleotide-binding protein G(I)/G(S)/G(O) subunit gamma-12 | -0.13 | 0.256895 |
| Q9CR68 | Cytochrome b-c1 complex subunit Rieske, mitochondrial;Cytochrome b-c1 complex subunit 11 | 0.06 | 0.256947 |
| Q61696 | Heat shock 70 kDa protein 1A;Heat shock 70 kDa protein 1B | 0.10 | 0.257072 |
| Q4VAA2 | Protein CDV3 | 0.12 | 0.257216 |
| P01867 | Ig gamma-2B chain C region | 0.19 | 0.257508 |
| Q5XJY5 | Coatomer subunit delta | -0.09 | 0.257606 |
| Q99PL6 | UBX domain-containing protein 6 | -0.16 | 0.257935 |
| Q8VCA8 | Secernin-2 | 0.12 | 0.257938 |
| P62889 | 60S ribosomal protein L30 | -0.07 | 0.2581 |
| P0CW02 | Lymphocyte antigen 6C1;Lymphocyte antigen 6C2 | 0.07 | 0.258169 |
| Q8BK64 | Activator of 90 kDa heat shock protein ATPase homolog 1 | 0.07 | 0.258308 |
| Q9D1C8 | Vacuolar protein sorting-associated protein 28 homolog | 0.13 | 0.258523 |
| Q7TPW1 | Nexilin | -0.08 | 0.258664 |
| Q68FE6 | Protein FAM65A | 0.15 | 0.258972 |
| Q61081 | Hsp90 co-chaperone Cdc37;Hsp90 co-chaperone Cdc37, N-terminally processed | 0.07 | 0.259028 |
| Q9JLI6 | Selenocysteine lyase | 0.10 | 0.259179 |
| Q80X90 | Filamin-B | 0.08 | 0.259263 |
| Q01730 | Ras suppressor protein 1 | -0.11 | 0.25935 |
| Q61743 | ATP-sensitive inward rectifier potassium channel 11 | 0.18 | 0.259749 |

| | | | |
|---|---|---|---|
| Q60749 | KH domain-containing, RNA-binding, signal transduction-associated protein 1 | -0.12 | 0.259769 |
| P99028 | Cytochrome b-c1 complex subunit 6, mitochondrial | 0.17 | 0.259913 |
| Q9CQQ7 | ATP synthase F(0) complex subunit B1, mitochondrial | -0.12 | 0.260198 |
| Q91ZF0 | DnaJ homolog subfamily C member 24 | 0.30 | 0.261731 |
| Q9D1R9 | 60S ribosomal protein L34 | 0.08 | 0.261832 |
| Q61206 | Platelet-activating factor acetylhydrolase IB subunit beta | 0.10 | 0.262296 |
| Q61337 | Bcl2-associated agonist of cell death | 2.13 | 0.262605 |
| O89114 | DnaJ homolog subfamily B member 5 | 0.16 | 0.262974 |
| Q8C7E7 | Starch-binding domain-containing protein 1 | 0.15 | 0.263724 |
| P51637 | Caveolin-3 | 0.09 | 0.263725 |
| Q9WUM3 | Coronin-1B | 0.17 | 0.264161 |
| Q3UHZ5 | Leiomodin-2 | 0.22 | 0.264613 |
| Q9CR42 | Ankyrin repeat domain-containing protein 1 | -0.21 | 0.264663 |
| O70400 | PDZ and LIM domain protein 1 | -0.08 | 0.265295 |
| Q71LX4 | Talin-2 | 0.08 | 0.265405 |
| Q8CCJ3 | E3 UFM1-protein ligase 1 | 0.13 | 0.265784 |
| Q9DBB8 | Trans-1,2-dihydrobenzene-1,2-diol dehydrogenase | 0.16 | 0.266346 |
| P61222 | ATP-binding cassette sub-family E member 1 | -0.12 | 0.266959 |
| Q8BIL5 | Protein Hook homolog 1 | 0.12 | 0.267854 |
| Q9ERU9 | E3 SUMO-protein ligase RanBP2 | -0.12 | 0.268047 |
| P97315 | Cysteine and glycine-rich protein 1 | 0.16 | 0.26842 |
| Q5SSL4 | Active breakpoint cluster region-related protein;Breakpoint cluster region protein | 0.11 | 0.268594 |
| Q9D6K8 | FUN14 domain-containing protein 2 | -0.23 | 0.269601 |
| P28650 | Adenylosuccinate synthetase isozyme 1 | 0.07 | 0.270093 |
| P49615 | Cyclin-dependent-like kinase 5 | -0.12 | 0.270727 |
| Q80X85 | 28S ribosomal protein S7, mitochondrial | 0.08 | 0.271003 |
| P49443 | Protein phosphatase 1A | 0.06 | 0.271013 |
| Q8BVG4 | Dipeptidyl peptidase 9 | -0.08 | 0.27158 |
| P33622 | Apolipoprotein C-III | -0.11 | 0.27169 |
| P00416 | Cytochrome c oxidase subunit 3 | -0.23 | 0.271699 |
| Q62318 | Transcription intermediary factor 1-beta | 0.14 | 0.272819 |
| Q6PHN9 | Ras-related protein Rab-35 | -0.11 | 0.2729 |
| Q99KB8 | Hydroxyacylglutathione hydrolase, mitochondrial | -0.16 | 0.273042 |
| A2AJI0 | MAP7 domain-containing protein 1 | -0.12 | 0.273286 |
| Q99KW3 | TRIO and F-actin-binding protein | 0.24 | 0.27339 |
| Q9DCT2 | NADH dehydrogenase [ubiquinone] iron-sulfur protein 3, mitochondrial | 0.07 | 0.275389 |
| Q9WTK3 | Glycosylphosphatidylinositol anchor attachment 1 protein | 0.16 | 0.27562 |
| Q60875 | Rho guanine nucleotide exchange factor 2 | 0.24 | 0.275732 |
| Q60930 | Voltage-dependent anion-selective channel protein 2 | -0.11 | 0.2759 |

| | | | |
|---|---|---|---|
| Q9DBG5 | Perilipin-3 | -0.11 | 0.276986 |
| P21614 | Vitamin D-binding protein | 0.15 | 0.27808 |
| Q9D2M8 | Ubiquitin-conjugating enzyme E2 variant 2 | 0.11 | 0.278519 |
| Q8R5J9 | PRA1 family protein 3 | 0.25 | 0.278724 |
| A3KGS3 | Ral GTPase-activating protein subunit alpha-2 | 0.48 | 0.280333 |
| P14576 | Signal recognition particle 54 kDa protein | 0.10 | 0.280778 |
| P47915 | 60S ribosomal protein L29 | -0.16 | 0.280969 |
| P82349 | Beta-sarcoglycan | -0.09 | 0.281873 |
| Q6P8M1 | Putative deoxyribonuclease TATDN1 | 0.52 | 0.282276 |
| O88653 | Ragulator complex protein LAMTOR3 | -0.10 | 0.282309 |
| P12970 | 60S ribosomal protein L7a | -0.09 | 0.282773 |
| B2RXS4 | Plexin-B2 | -0.09 | 0.28341 |
| P54071 | Isocitrate dehydrogenase [NADP], mitochondrial | -0.07 | 0.283416 |
| P56393 | Cytochrome c oxidase subunit 7B, mitochondrial | 0.13 | 0.283723 |
| Q70KF4 | Cardiomyopathy-associated protein 5 | 0.06 | 0.285183 |
| P56391 | Cytochrome c oxidase subunit 6B1 | -0.10 | 0.286787 |
| Q8K4Z3 | NAD(P)H-hydrate epimerase | -0.07 | 0.287237 |
| Q6P4T2 | U5 small nuclear ribonucleoprotein 200 kDa helicase | 0.16 | 0.28729 |
| Q3UMB9 | WASH complex subunit 7 | 0.22 | 0.287452 |
| Q9DCS3 | Trans-2-enoyl-CoA reductase, mitochondrial | 0.14 | 0.287456 |
| Q9JJI8 | 60S ribosomal protein L38 | -0.25 | 0.288076 |
| Q8BMK4 | Cytoskeleton-associated protein 4 | 0.06 | 0.288487 |
| Q8BH59 | Calcium-binding mitochondrial carrier protein Aralar1 | 0.07 | 0.289574 |
| Q61335 | B-cell receptor-associated protein 31 | -0.23 | 0.289847 |
| Q91VW5 | Golgin subfamily A member 4 | 0.09 | 0.289952 |
| Q8K2Y7 | 39S ribosomal protein L47, mitochondrial | -0.08 | 0.290289 |
| Q3ULJ0 | Glycerol-3-phosphate dehydrogenase 1-like protein | 0.09 | 0.290485 |
| P29699 | Alpha-2-HS-glycoprotein | 0.07 | 0.29049 |
| P56380 | Bis(5-nucleosyl)-tetraphosphatase [asymmetrical] | 0.07 | 0.290518 |
| P99029 | Peroxiredoxin-5, mitochondrial | -0.10 | 0.290918 |
| Q921H8 | 3-ketoacyl-CoA thiolase A, peroxisomal;3-ketoacyl-CoA thiolase B, peroxisomal | -0.06 | 0.291131 |
| Q8BXN7 | Protein phosphatase 1K, mitochondrial | 0.12 | 0.2913 |
| Q149B8 | PGC-1 and ERR-induced regulator in muscle protein 1 | -0.08 | 0.29165 |
| O55026 | Ectonucleoside triphosphate diphosphohydrolase 2 | 0.09 | 0.291787 |
| Q9CZR8 | Elongation factor Ts, mitochondrial | -0.10 | 0.291917 |
| Q91WK0 | Leucine-rich repeat flightless-interacting protein 2 | 0.11 | 0.292076 |
| Q99L13 | 3-hydroxyisobutyrate dehydrogenase, mitochondrial | 0.07 | 0.292534 |
| Q8R0P4 | Mth938 domain-containing protein | 0.07 | 0.292583 |
| Q91V64 | Isochorismatase domain-containing protein 1 | 0.10 | 0.293053 |
| Q9QXC1 | Fetuin-B | 0.15 | 0.293069 |
| Q7TQ95 | Protein lunapark | -0.23 | 0.295227 |

| | | | |
|---|---|---|---|
| Q8BTG7 | Protein NDRG4 | 0.11 | 0.295396 |
| O35226 | 26S proteasome non-ATPase regulatory subunit 4 | -0.22 | 0.296153 |
| Q8BUV3 | Gephyrin;Molybdopterin adenylyltransferase;Molybdopterin molybdenumtransferase | -0.23 | 0.29617 |
| Q3KNJ2 | Non-homologous end-joining factor 1 | -0.12 | 0.296562 |
| Q04690 | Neurofibromin | 0.29 | 0.297192 |
| Q00724 | Retinol-binding protein 4 | -0.17 | 0.298269 |
| P35564 | Calnexin | 0.07 | 0.298814 |
| Q08481 | Platelet endothelial cell adhesion molecule | 0.11 | 0.298855 |
| P58059 | 28S ribosomal protein S21, mitochondrial | 0.21 | 0.298966 |
| Q91YQ5 | Dolichyl-diphosphooligosaccharide--protein glycosyltransferase subunit 1 | 0.06 | 0.299103 |
| Q61147 | Ceruloplasmin | -0.13 | 0.299508 |
| Q9DCT5 | Stromal cell-derived factor 2 | 0.15 | 0.299575 |
| P50518 | V-type proton ATPase subunit E 1 | 0.07 | 0.303057 |
| Q8R3V5 | Endophilin-B2 | -0.11 | 0.303428 |
| Q61035 | Histidine--tRNA ligase, cytoplasmic | 0.06 | 0.303555 |
| Q9CQ71 | Replication protein A 14 kDa subunit | 0.13 | 0.30474 |
| Q61233 | Plastin-2 | 0.32 | 0.30541 |
| Q7TSC1 | Protein PRRC2A | -0.09 | 0.306311 |
| Q8K021 | Secretory carrier-associated membrane protein 1 | 0.16 | 0.30642 |
| Q99JB8 | Protein kinase C and casein kinase II substrate protein 3 | 0.05 | 0.307933 |
| P61027 | Ras-related protein Rab-10 | -0.09 | 0.308018 |
| Q8CBY8 | Dynactin subunit 4 | 0.11 | 0.309013 |
| Q03141 | MAP/microtubule affinity-regulating kinase 3 | -0.20 | 0.309241 |
| Q9CR16 | Peptidyl-prolyl cis-trans isomerase D | 0.39 | 0.309295 |
| O70468 | Myosin-binding protein C, cardiac-type | -0.05 | 0.309349 |
| Q60864 | Stress-induced-phosphoprotein 1 | 0.07 | 0.30958 |
| Q8VE95 | UPF0598 protein C8orf82 homolog | 0.14 | 0.309725 |
| Q8R4H2 | Rho guanine nucleotide exchange factor 12 | 0.13 | 0.310156 |
| Q8CI51 | PDZ and LIM domain protein 5 | 0.07 | 0.310295 |
| P21278 | Guanine nucleotide-binding protein subunit alpha-11 | 0.17 | 0.311088 |
| Q9EPM5 | Syncoilin | 0.10 | 0.311233 |
| Q920Q8 | Influenza virus NS1A-binding protein homolog | 0.24 | 0.312589 |
| Q61191 | Host cell factor 1;HCF N-terminal chain 1;HCF N-terminal chain 2;HCF N-terminal chain 3;HCF N-terminal chain 4;HCF N-terminal chain 5;HCF N-terminal chain 6;HCF C-terminal chain 1;HCF C-terminal chain 2;HCF C-terminal chain 3;HCF C-terminal chain 4;HCF C-terminal chain 5;HCF C-terminal chain 6 | 0.23 | 0.313445 |
| Q9D1N9 | 39S ribosomal protein L21, mitochondrial | 0.19 | 0.313538 |
| Q7TNC4 | Putative RNA-binding protein Luc7-like 2 | 0.08 | 0.314439 |

| | | | |
|---|---|---|---|
| O35639 | Annexin A3 | 0.05 | 0.314538 |
| Q9D287 | Pre-mRNA-splicing factor SPF27 | 0.17 | 0.315774 |
| Q01768 | Nucleoside diphosphate kinase B | 0.09 | 0.315797 |
| Q80ZW2 | Protein THEM6 | 0.16 | 0.316976 |
| P05063 | Fructose-bisphosphate aldolase C | 0.56 | 0.31739 |
| P41105 | 60S ribosomal protein L28 | -0.17 | 0.317419 |
| P61082 | NEDD8-conjugating enzyme Ubc12 | -0.07 | 0.317576 |
| P10854 | Histone H2B type 1-M;Histone H2B type 1-P;Histone H2B type 1-A | 0.67 | 0.317776 |
| Q63918 | Serum deprivation-response protein | -0.06 | 0.318205 |
| P17426 | AP-2 complex subunit alpha-1 | 0.17 | 0.318437 |
| Q62048 | Astrocytic phosphoprotein PEA-15 | -0.11 | 0.31908 |
| P70302 | Stromal interaction molecule 1 | 0.47 | 0.319816 |
| Q8K2Q5 | Coiled-coil-helix-coiled-coil-helix domain-containing protein 7 | 0.11 | 0.320432 |
| Q61207 | Prosaposin | 0.07 | 0.320705 |
| Q9D1K7 | UPF0687 protein C20orf27 homolog | -0.19 | 0.321075 |
| Q9D1H8 | 39S ribosomal protein L53, mitochondrial | 0.08 | 0.321593 |
| P47962 | 60S ribosomal protein L5 | 0.06 | 0.322802 |
| Q00PI9 | Heterogeneous nuclear ribonucleoprotein U-like protein 2 | 0.12 | 0.322853 |
| Q91VW3 | SH3 domain-binding glutamic acid-rich-like protein 3 | 0.12 | 0.323001 |
| P03930 | ATP synthase protein 8 | 0.17 | 0.323273 |
| Q9Z1Q9 | Valine--tRNA ligase | 0.05 | 0.323422 |
| Q9CZ44 | NSFL1 cofactor p47 | -0.14 | 0.32408 |
| P97461 | 40S ribosomal protein S5;40S ribosomal protein S5, N-terminally processed | 0.11 | 0.324393 |
| Q80U63 | Mitofusin-2 | -0.08 | 0.325094 |
| Q9R1Q7 | Proteolipid protein 2 | -0.33 | 0.325251 |
| Q8VCM7 | Fibrinogen gamma chain | -0.13 | 0.325497 |
| O35387 | HCLS1-associated protein X-1 | -0.08 | 0.325512 |
| P27773 | Protein disulfide-isomerase A3 | 0.05 | 0.325923 |
| Q9EQH3 | Vacuolar protein sorting-associated protein 35 | -0.12 | 0.326631 |
| Q61210 | Rho guanine nucleotide exchange factor 1 | -0.10 | 0.326644 |
| Q9JJE7 | Fatty acid desaturase 3 | 0.07 | 0.326794 |
| Q3TLH4 | Protein PRRC2C | 0.10 | 0.326844 |
| Q7TT50 | Serine/threonine-protein kinase MRCK beta | -0.16 | 0.327047 |
| Q9Z2Z6 | Mitochondrial carnitine/acylcarnitine carrier protein | 0.08 | 0.327051 |
| B1AR13 | CDGSH iron-sulfur domain-containing protein 3, mitochondrial | 0.17 | 0.327259 |
| P63248 | cAMP-dependent protein kinase inhibitor alpha | 0.14 | 0.327384 |
| P10518 | Delta-aminolevulinic acid dehydratase | 0.11 | 0.328306 |
| O35643 | AP-1 complex subunit beta-1 | -0.09 | 0.328503 |

| | | | |
|---|---|---|---|
| Q9JMC3 | DnaJ homolog subfamily A member 4 | -0.05 | 0.328608 |
| Q922E4 | Ethanolamine-phosphate cytidylyltransferase | -0.10 | 0.329595 |
| P62274 | 40S ribosomal protein S29 | -0.12 | 0.329943 |
| Q8VEM8 | Phosphate carrier protein, mitochondrial | 0.10 | 0.330067 |
| Q62433 | Protein NDRG1 | 0.20 | 0.331175 |
| Q04857 | Collagen alpha-1(VI) chain | 0.08 | 0.331597 |
| Q8BSF4 | Phosphatidylserine decarboxylase proenzyme;Phosphatidylserine decarboxylase alpha chain;Phosphatidylserine decarboxylase beta chain | 0.11 | 0.332674 |
| Q8CFI5 | Probable proline--tRNA ligase, mitochondrial | 0.33 | 0.332718 |
| Q9WVA4 | Transgelin-2 | 0.26 | 0.332819 |
| Q9D7J4 | Cytochrome c oxidase protein 20 homolog | 0.21 | 0.333658 |
| P31230 | Aminoacyl tRNA synthase complex-interacting multifunctional protein 1;Endothelial monocyte-activating polypeptide 2 | -0.08 | 0.334411 |
| P84078 | ADP-ribosylation factor 1;ADP-ribosylation factor 3;ADP-ribosylation factor 2 | 0.19 | 0.33493 |
| P28660 | Nck-associated protein 1 | 0.08 | 0.335464 |
| P62075 | Mitochondrial import inner membrane translocase subunit Tim13 | -0.08 | 0.336197 |
| Q8K1A6 | Coiled-coil and C2 domain-containing protein 1A | 0.27 | 0.336637 |
| P61148 | Fibroblast growth factor 1 | 0.14 | 0.33721 |
| P11152 | Lipoprotein lipase | 0.06 | 0.337419 |
| Q91YM4 | Protein TBRG4 | -0.09 | 0.338012 |
| Q9QZH6 | Evolutionarily conserved signaling intermediate in Toll pathway, mitochondrial | -0.10 | 0.338442 |
| Q9JKP5 | Muscleblind-like protein 1 | -0.09 | 0.339374 |
| P55264 | Adenosine kinase | -0.07 | 0.339622 |
| P35550 | rRNA 2-O-methyltransferase fibrillarin | 0.06 | 0.339806 |
| Q8C5H8 | NAD kinase 2, mitochondrial | 0.10 | 0.340109 |
| Q9CQZ5 | NADH dehydrogenase [ubiquinone] 1 alpha subcomplex subunit 6 | 0.06 | 0.340881 |
| Q9ES83 | Blood vessel epicardial substance | -0.08 | 0.340938 |
| Q9Z1Q2 | Abhydrolase domain-containing protein 16A | -0.16 | 0.34137 |
| O70591 | Prefoldin subunit 2 | -0.07 | 0.341845 |
| Q61941 | NAD(P) transhydrogenase, mitochondrial | 0.13 | 0.341967 |
| O35737 | Heterogeneous nuclear ribonucleoprotein H;Heterogeneous nuclear ribonucleoprotein H, N-terminally processed;Heterogeneous nuclear ribonucleoprotein H2 | -0.15 | 0.3435 |
| Q9Z2A9 | Gamma-glutamyltransferase 5;Gamma-glutamyltransferase 5 heavy chain;Gamma-glutamyltransferase 5 light chain | 0.08 | 0.343907 |
| Q99K48 | Non-POU domain-containing octamer-binding protein | 0.08 | 0.343915 |
| Q9JIF9 | Myotilin | 0.12 | 0.344139 |

| | | | |
|---|---|---|---|
| P59017 | Bcl-2-like protein 13 | 0.11 | 0.344538 |
| Q5FWK3 | Rho GTPase-activating protein 1 | 0.09 | 0.344691 |
| Q9D7A8 | Armadillo repeat-containing protein 1 | -0.08 | 0.344692 |
| P14685 | 26S proteasome non-ATPase regulatory subunit 3 | 0.08 | 0.345015 |
| Q8CHQ0 | F-box only protein 4 | 0.26 | 0.34537 |
| Q9CR09 | Ubiquitin-fold modifier-conjugating enzyme 1 | -0.08 | 0.345501 |
| Q7TMF3 | NADH dehydrogenase [ubiquinone] 1 alpha subcomplex subunit 12 | 0.12 | 0.346722 |
| A2AQ25 | Sickle tail protein | 0.10 | 0.347012 |
| Q9R0H2 | Endomucin | -0.27 | 0.347018 |
| Q8R0H9 | ADP-ribosylation factor-binding protein GGA1 | -0.11 | 0.34751 |
| Q9DC07 | LIM zinc-binding domain-containing Nebulette | 0.11 | 0.347865 |
| P62911 | 60S ribosomal protein L32 | -0.10 | 0.348483 |
| Q9EQS3 | C-Myc-binding protein | -0.13 | 0.349062 |
| Q8VE38 | Oxidoreductase NAD-binding domain-containing protein 1 | -0.11 | 0.349146 |
| Q9D338 | 39S ribosomal protein L19, mitochondrial | 0.09 | 0.350695 |
| Q8BPB5 | EGF-containing fibulin-like extracellular matrix protein 1 | 0.17 | 0.351299 |
| Q99LY9 | NADH dehydrogenase [ubiquinone] iron-sulfur protein 5;NADH dehydrogenase [ubiquinone] iron-sulfur protein 5, N-terminally processed | -0.16 | 0.35143 |
| Q9JKF7 | 39S ribosomal protein L39, mitochondrial | 0.06 | 0.351556 |
| P14211 | Calreticulin | 0.09 | 0.352366 |
| Q9CQR4 | Acyl-coenzyme A thioesterase 13;Acyl-coenzyme A thioesterase 13, N-terminally processed | -0.07 | 0.35241 |
| P47963 | 60S ribosomal protein L13 | -0.19 | 0.354185 |
| Q6P5H2 | Nestin | -0.06 | 0.356149 |
| Q8BWW4 | La-related protein 4 | 0.10 | 0.357834 |
| Q9DCJ5 | NADH dehydrogenase [ubiquinone] 1 alpha subcomplex subunit 8 | 0.06 | 0.358093 |
| Q6ZQ58 | La-related protein 1 | 0.14 | 0.358549 |
| O08788 | Dynactin subunit 1 | 0.06 | 0.358923 |
| Q7TSH2 | Phosphorylase b kinase regulatory subunit beta | 0.15 | 0.359711 |
| O88569 | Heterogeneous nuclear ribonucleoproteins A2/B1 | 0.08 | 0.360518 |
| Q91ZU6 | Dystonin | -0.07 | 0.360701 |
| P62307 | Small nuclear ribonucleoprotein F | -0.27 | 0.361212 |
| Q01339 | Beta-2-glycoprotein 1 | 0.05 | 0.361421 |
| Q61830 | Macrophage mannose receptor 1 | -0.09 | 0.361519 |
| Q9CX00 | IST1 homolog | 0.09 | 0.362611 |
| Q3U0B3 | Dehydrogenase/reductase SDR family member 11 | 0.07 | 0.36278 |
| Q99N84 | 28S ribosomal protein S18b, mitochondrial | 0.15 | 0.363433 |
| Q61768 | Kinesin-1 heavy chain | 0.06 | 0.363586 |

| | | | |
|---|---|---|---|
| Q5SUC9 | Protein SCO1 homolog, mitochondrial | -0.11 | 0.363728 |
| P70452 | Syntaxin-4 | -0.13 | 0.364432 |
| Q9Z1X4 | Interleukin enhancer-binding factor 3 | 0.06 | 0.36488 |
| Q6NSR8 | Probable aminopeptidase NPEPL1 | -0.09 | 0.36584 |
| P62281 | 40S ribosomal protein S11 | 0.05 | 0.367081 |
| Q9CXI0 | 2-methoxy-6-polyprenyl-1,4-benzoquinol methylase, mitochondrial | 0.09 | 0.367214 |
| P70398 | Probable ubiquitin carboxyl-terminal hydrolase FAF-X | 0.10 | 0.367279 |
| Q9DBS1 | Transmembrane protein 43 | -0.06 | 0.367706 |
| Q91W86 | Vacuolar protein sorting-associated protein 11 homolog | -0.11 | 0.367981 |
| P16125 | L-lactate dehydrogenase B chain | 0.05 | 0.369101 |
| P47934 | Carnitine O-acetyltransferase | 0.05 | 0.36973 |
| Q8BK72 | 28S ribosomal protein S27, mitochondrial | 0.17 | 0.370111 |
| Q62417 | Sorbin and SH3 domain-containing protein 1 | 0.10 | 0.370126 |
| Q91ZX7 | Prolow-density lipoprotein receptor-related protein 1;Low-density lipoprotein receptor-related protein 1 85 kDa subunit;Low-density lipoprotein receptor-related protein 1 515 kDa subunit;Low-density lipoprotein receptor-related protein 1 intracellular domain | -0.15 | 0.370535 |
| Q9WUB3 | Glycogen phosphorylase, muscle form | -0.06 | 0.370751 |
| Q9QZ85 | Interferon-inducible GTPase 1 | -0.12 | 0.370844 |
| Q4PJX1 | Protein odr-4 homolog | 0.13 | 0.371153 |
| P0DN34 | | 0.13 | 0.371158 |
| Q91VC3 | Eukaryotic initiation factor 4A-III;Eukaryotic initiation factor 4A-III, N-terminally processed | 0.05 | 0.371971 |
| Q91XC8 | Death-associated protein 1 | 0.07 | 0.37237 |
| Q91XU3 | Phosphatidylinositol 5-phosphate 4-kinase type-2 gamma | -0.21 | 0.373094 |
| Q8BK63 | Casein kinase I isoform alpha | 0.11 | 0.373217 |
| Q9DC70 | NADH dehydrogenase [ubiquinone] iron-sulfur protein 7, mitochondrial | 0.06 | 0.375177 |
| Q9D024 | Coiled-coil domain-containing protein 47 | 0.08 | 0.376553 |
| Q9D6Y7 | Mitochondrial peptide methionine sulfoxide reductase | 0.09 | 0.376638 |
| P51163 | Uroporphyrinogen-III synthase | 0.21 | 0.377021 |
| Q9DBF1 | Alpha-aminoadipic semialdehyde dehydrogenase | 0.10 | 0.377634 |
| O55023 | Inositol monophosphatase 1 | 0.15 | 0.377778 |
| Q91WK1 | SPRY domain-containing protein 4 | -0.08 | 0.378538 |
| Q99KQ4 | Nicotinamide phosphoribosyltransferase | -0.05 | 0.378918 |
| Q8K3K8 | Optineurin | 0.13 | 0.378989 |
| Q9D328 | Transmembrane protein 35 | 0.13 | 0.379504 |
| Q9CQW1 | Synaptobrevin homolog YKT6 | -0.07 | 0.379557 |
| P00329 | Alcohol dehydrogenase 1 | 0.09 | 0.381023 |
| Q9CR00 | 26S proteasome non-ATPase regulatory subunit 9 | 0.11 | 0.381348 |
| Q9CWS0 | N(G),N(G)-dimethylarginine dimethylaminohydrolase 1 | 0.09 | 0.382065 |

| | | | |
|---|---|---|---|
| Q8VCM3 | Zinc finger FYVE domain-containing protein 21 | -0.09 | 0.382737 |
| Q9CX30 | Protein YIF1B | 0.08 | 0.383343 |
| P05064 | Fructose-bisphosphate aldolase A | 0.05 | 0.383932 |
| Q8BGS2 | BolA-like protein 2 | 0.06 | 0.384394 |
| P07759 | Serine protease inhibitor A3K | 0.07 | 0.384889 |
| O35593 | 26S proteasome non-ATPase regulatory subunit 14 | 0.35 | 0.385548 |
| O88207 | Collagen alpha-1(V) chain;Collagen alpha-1(XI) chain | 0.17 | 0.385562 |
| O08663 | Methionine aminopeptidase 2 | 0.08 | 0.387054 |
| Q8C854 | Myelin expression factor 2 | -1.80 | 0.387636 |
| P55302 | Alpha-2-macroglobulin receptor-associated protein | 0.05 | 0.387941 |
| P24270 | Catalase | 0.07 | 0.38834 |
| Q8BMS4 | Ubiquinone biosynthesis O-methyltransferase, mitochondrial | 0.07 | 0.388466 |
| Q99N96 | 39S ribosomal protein L1, mitochondrial | 0.10 | 0.389159 |
| Q8BU30 | Isoleucine--tRNA ligase, cytoplasmic | -0.21 | 0.38972 |
| P17156 | Heat shock-related 70 kDa protein 2 | 0.05 | 0.390726 |
| Q9CQ92 | Mitochondrial fission 1 protein | 0.09 | 0.392087 |
| Q811D0 | Disks large homolog 1 | -0.06 | 0.39261 |
| Q9D773 | 39S ribosomal protein L2, mitochondrial | 0.05 | 0.395731 |
| Q9R1Z8 | Vinexin | 0.28 | 0.395753 |
| Q9CZ30 | Obg-like ATPase 1 | 0.05 | 0.396409 |
| Q9DCG9 | Multifunctional methyltransferase subunit TRM112-like protein | 0.10 | 0.396639 |
| P08074 | Carbonyl reductase [NADPH] 2 | 0.07 | 0.397292 |
| Q9CYZ2 | Tumor protein D54 | 0.18 | 0.398037 |
| Q99MD9 | Nuclear autoantigenic sperm protein | 0.10 | 0.398263 |
| P61087 | Ubiquitin-conjugating enzyme E2 K | -0.12 | 0.398498 |
| Q9JMH6 | Thioredoxin reductase 1, cytoplasmic | 0.16 | 0.398991 |
| Q9JLT4 | Thioredoxin reductase 2, mitochondrial | -0.12 | 0.399343 |
| Q91VT4 | Carbonyl reductase family member 4 | 0.05 | 0.401457 |
| P42669 | Transcriptional activator protein Pur-alpha | -0.07 | 0.401569 |
| Q8VHK1 | Caskin-2 | 0.52 | 0.403214 |
| Q9DCT8 | Cysteine-rich protein 2 | 0.06 | 0.403844 |
| Q9QYJ0 | DnaJ homolog subfamily A member 2 | 0.08 | 0.40504 |
| Q6P3A8 | 2-oxoisovalerate dehydrogenase subunit beta, mitochondrial | 0.04 | 0.406653 |
| O35345 | Importin subunit alpha-7 | 0.15 | 0.406753 |
| Q91YD9 | Neural Wiskott-Aldrich syndrome protein | 0.14 | 0.407244 |
| Q9QZJ6 | Microfibrillar-associated protein 5 | -0.08 | 0.407277 |
| Q62009 | Periostin | 0.07 | 0.407602 |
| P07356 | Annexin A2 | 0.04 | 0.407987 |
| Q5IRJ6 | Zinc transporter 9 | -0.06 | 0.408526 |

| | | | |
|---|---|---|---|
| Q08093 | Calponin-2 | 0.09 | 0.409752 |
| Q9DB27 | Malignant T-cell-amplified sequence 1;Malignant T-cell-amplified sequence 2 | 0.10 | 0.409915 |
| P35330 | Intercellular adhesion molecule 2 | -0.06 | 0.410123 |
| P50285 | Dimethylaniline monooxygenase [N-oxide-forming] 1 | -0.10 | 0.410192 |
| Q9D3R6 | Katanin p60 ATPase-containing subunit A-like 2;Spermatogenesis-associated protein 5 | -0.15 | 0.41055 |
| Q8C5W3 | Tubulin-specific chaperone cofactor E-like protein | 0.21 | 0.410757 |
| Q2NL51 | Glycogen synthase kinase-3 alpha | 0.10 | 0.410774 |
| A2AAJ9 | Obscurin | 0.06 | 0.411929 |
| Q9QYC0 | Alpha-adducin | 0.14 | 0.412348 |
| Q80W54 | CAAX prenyl protease 1 homolog | 0.17 | 0.412423 |
| Q6A0A9 | Constitutive coactivator of PPAR-gamma-like protein 1 | 0.06 | 0.41245 |
| Q8R1V4 | Transmembrane emp24 domain-containing protein 4 | -0.05 | 0.413022 |
| Q60597 | 2-oxoglutarate dehydrogenase, mitochondrial | 0.04 | 0.413076 |
| Q6P8I4 | PEST proteolytic signal-containing nuclear protein | 0.13 | 0.414617 |
| Q8VD26 | Transmembrane protein 143 | -0.06 | 0.415563 |
| P08249 | Malate dehydrogenase, mitochondrial | 0.06 | 0.41607 |
| P13634 | Carbonic anhydrase 1 | 0.11 | 0.416319 |
| Q9D1G1 | Ras-related protein Rab-1B | -0.07 | 0.416695 |
| Q6A0A2 | La-related protein 4B | 0.10 | 0.417246 |
| P70336 | Rho-associated protein kinase 2 | -0.05 | 0.41811 |
| P53026 | 60S ribosomal protein L10a | -0.08 | 0.418324 |
| P68510 | 14-3-3 protein eta | -0.15 | 0.418489 |
| Q6PCP5 | Mitochondrial fission factor | -0.05 | 0.418569 |
| O35153 | BET1-like protein | 0.08 | 0.420112 |
| Q6ZPE2 | Myotubularin-related protein 5 | 0.07 | 0.420518 |
| Q8CIE6 | Coatomer subunit alpha;Xenin;Proxenin | 0.05 | 0.420566 |
| P35700 | Peroxiredoxin-1 | 0.05 | 0.420666 |
| Q924C5 | Alpha-protein kinase 3 | -0.06 | 0.420829 |
| P20357 | Microtubule-associated protein 2 | 0.35 | 0.421347 |
| Q8CGY8 | UDP-N-acetylglucosamine--peptide N-acetylglucosaminyltransferase 110 kDa subunit | 0.11 | 0.422572 |
| Q91Z53 | Glyoxylate reductase/hydroxypyruvate reductase | 0.05 | 0.423258 |
| Q9Z0G2 | SRSF protein kinase 3 | -0.07 | 0.423644 |
| Q76LL6 | FH1/FH2 domain-containing protein 3 | 0.11 | 0.425175 |
| Q922B1 | O-acetyl-ADP-ribose deacetylase MACROD1 | 0.06 | 0.425493 |
| Q91VR5 | ATP-dependent RNA helicase DDX1 | 0.07 | 0.426472 |
| P09542 | Myosin light chain 3 | 0.05 | 0.426553 |
| Q91YN9 | BAG family molecular chaperone regulator 2 | 0.49 | 0.427174 |
| O35295 | Transcriptional activator protein Pur-beta | -0.09 | 0.427237 |
| P50396 | Rab GDP dissociation inhibitor alpha | 0.05 | 0.427637 |

| | | | |
|---|---|---|---|
| P17742 | Peptidyl-prolyl cis-trans isomerase A;Peptidyl-prolyl cis-trans isomerase A, N-terminally processed | -0.04 | 0.428818 |
| P10605 | Cathepsin B;Cathepsin B light chain;Cathepsin B heavy chain | -0.14 | 0.429665 |
| O09161 | Calsequestrin-2 | -0.05 | 0.429802 |
| O54734 | Dolichyl-diphosphooligosaccharide--protein glycosyltransferase 48 kDa subunit | -0.13 | 0.43005 |
| Q5SV77 | Gametogenetin-binding protein 2 | 0.17 | 0.43009 |
| P97371 | Proteasome activator complex subunit 1 | -0.10 | 0.43036 |
| Q91VJ2 | Protein kinase C delta-binding protein | 0.23 | 0.430386 |
| P07310 | Creatine kinase M-type | 0.04 | 0.430503 |
| Q6ZWU9 | 40S ribosomal protein S27;40S ribosomal protein S27-like | -0.06 | 0.430707 |
| O55126 | Protein NipSnap homolog 2 | -0.05 | 0.430761 |
| Q9DB26 | Phytanoyl-CoA dioxygenase domain-containing protein 1 | 0.40 | 0.430801 |
| Q61656 | Probable ATP-dependent RNA helicase DDX5 | 0.08 | 0.430885 |
| Q64105 | Sepiapterin reductase | 0.09 | 0.431453 |
| Q9D924 | Iron-sulfur cluster assembly 1 homolog, mitochondrial | -0.05 | 0.432698 |
| Q91WC3 | Long-chain-fatty-acid--CoA ligase 6 | 0.16 | 0.43271 |
| Q9CQI3 | Glia maturation factor beta;Glia maturation factor gamma | -0.11 | 0.434214 |
| Q9CWD8 | Iron-sulfur protein NUBPL | 0.05 | 0.434608 |
| Q02013 | Aquaporin-1 | -0.08 | 0.434962 |
| P32020 | Non-specific lipid-transfer protein | 0.08 | 0.435341 |
| Q99N93 | 39S ribosomal protein L16, mitochondrial | -0.24 | 0.435741 |
| Q61699 | Heat shock protein 105 kDa | 0.14 | 0.435851 |
| Q63961 | Endoglin | 0.08 | 0.436207 |
| Q6P069 | Sorcin | 0.06 | 0.437225 |
| P15508 | Spectrin beta chain, erythrocytic | 0.05 | 0.437508 |
| P11983 | T-complex protein 1 subunit alpha | -0.06 | 0.438645 |
| P09925 | Surfeit locus protein 1 | -0.10 | 0.43889 |
| Q9JL56 | Glycerophosphodiester phosphodiesterase 1 | -0.08 | 0.439457 |
| P70698 | CTP synthase 1 | -0.16 | 0.440813 |
| O70404 | Vesicle-associated membrane protein 8 | -1.45 | 0.440826 |
| Q99LF4 | tRNA-splicing ligase RtcB homolog | 0.04 | 0.441876 |
| P26040 | Ezrin | 0.07 | 0.442299 |
| Q9D8T7 | SRA stem-loop-interacting RNA-binding protein, mitochondrial | 0.07 | 0.442942 |
| Q8BGY7 | Protein FAM210A | -0.09 | 0.44301 |
| Q8BWF0 | Succinate-semialdehyde dehydrogenase, mitochondrial | 0.06 | 0.443261 |
| Q9Z2P8 | Vesicle-associated membrane protein 5 | 0.16 | 0.444158 |

| | | | |
|---|---|---|---|
| Q9R1T2 | SUMO-activating enzyme subunit 1;SUMO-activating enzyme subunit 1, N-terminally processed | 0.04 | 0.444264 |
| Q78PY7 | Staphylococcal nuclease domain-containing protein 1 | 0.04 | 0.445732 |
| P43024 | Cytochrome c oxidase subunit 6A1, mitochondrial | 0.11 | 0.445772 |
| Q499X9 | Methionine--tRNA ligase, mitochondrial | -0.05 | 0.446155 |
| Q3UIZ8 | Myosin light chain kinase 3 | -0.07 | 0.446533 |
| O35855 | Branched-chain-amino-acid aminotransferase, mitochondrial | -0.09 | 0.44694 |
| P14869 | 60S acidic ribosomal protein P0 | -0.08 | 0.447536 |
| Q9QZZ6 | Dermatopontin | 0.17 | 0.447939 |
| Q9D8W5 | 26S proteasome non-ATPase regulatory subunit 12 | -0.08 | 0.448614 |
| Q9EQ80 | NIF3-like protein 1 | -0.17 | 0.448685 |
| P80317 | T-complex protein 1 subunit zeta | -0.04 | 0.448725 |
| P63094 | Guanine nucleotide-binding protein G(s) subunit alpha isoforms short;Guanine nucleotide-binding protein G(s) subunit alpha isoforms XLas | 0.07 | 0.449175 |
| Q9CPW4 | Actin-related protein 2/3 complex subunit 5 | 0.21 | 0.44947 |
| Q03265 | ATP synthase subunit alpha, mitochondrial | -0.05 | 0.44998 |
| Q60932 | Voltage-dependent anion-selective channel protein 1 | -0.06 | 0.45037 |
| Q61097 | Kinase suppressor of Ras 1 | -0.08 | 0.451619 |
| Q80UU1 | Ankyrin repeat and zinc finger domain-containing protein 1 | 0.24 | 0.451778 |
| Q9DCC4 | Pyrroline-5-carboxylate reductase 3 | 0.06 | 0.452146 |
| P17427 | AP-2 complex subunit alpha-2 | -0.08 | 0.45309 |
| Q4U4S6 | Xin actin-binding repeat-containing protein 2 | 0.06 | 0.453336 |
| Q9D6K5 | Synaptojanin-2-binding protein | -0.10 | 0.453527 |
| B1AXP6 | Mitochondrial import receptor subunit TOM5 homolog | -0.07 | 0.457576 |
| Q8CI08 | SLAIN motif-containing protein 2 | 0.22 | 0.45798 |
| Q9QZ57 | Heat shock protein beta-3 | -0.07 | 0.458054 |
| P28665 | Murinoglobulin-1 | -0.04 | 0.458248 |
| Q9CZY3 | Ubiquitin-conjugating enzyme E2 variant 1 | 0.05 | 0.458303 |
| Q61730 | Interleukin-1 receptor accessory protein | -0.07 | 0.459122 |
| P60843 | Eukaryotic initiation factor 4A-I | -0.05 | 0.459885 |
| Q8K411 | Presequence protease, mitochondrial | 0.05 | 0.460136 |
| Q8BRF7 | Sec1 family domain-containing protein 1 | -0.10 | 0.460221 |
| P26883 | Peptidyl-prolyl cis-trans isomerase FKBP1A | 0.05 | 0.461903 |
| P51410 | 60S ribosomal protein L9 | 0.05 | 0.462404 |
| P08226 | Apolipoprotein E | -0.04 | 0.462554 |
| P26369 | Splicing factor U2AF 65 kDa subunit | 0.05 | 0.462712 |
| Q9CZW5 | Mitochondrial import receptor subunit TOM70 | -0.10 | 0.464134 |
| Q148V7 | LisH domain and HEAT repeat-containing protein KIAA1468 | 0.52 | 0.464403 |
| Q9Z1T1 | AP-3 complex subunit beta-1 | 0.04 | 0.464804 |
| Q6A4J8 | Ubiquitin carboxyl-terminal hydrolase 7 | -0.04 | 0.465283 |

| | | | |
|---|---|---|---|
| Q02566 | Myosin-6 | 0.04 | 0.465702 |
| Q6GQT9 | Nodal modulator 1 | -0.06 | 0.465727 |
| Q9Z2I0 | LETM1 and EF-hand domain-containing protein 1, mitochondrial | 0.05 | 0.466319 |
| Q9D0W5 | Peptidyl-prolyl cis-trans isomerase-like 1 | -0.07 | 0.466534 |
| Q9JLV5 | Cullin-3 | 0.05 | 0.467999 |
| Q8K215 | LYR motif-containing protein 4 | -0.07 | 0.470674 |
| Q91W50 | Cold shock domain-containing protein E1 | 0.05 | 0.471228 |
| Q8VED9 | Galectin-related protein | -0.07 | 0.471773 |
| Q9D1B9 | 39S ribosomal protein L28, mitochondrial | -0.15 | 0.47261 |
| Q91XE4 | N-acyl-aromatic-L-amino acid amidohydrolase (carboxylate-forming) | 0.05 | 0.472803 |
| P16045 | Galectin-1 | 0.05 | 0.472815 |
| Q61316 | Heat shock 70 kDa protein 4 | 0.04 | 0.473129 |
| P11404 | Fatty acid-binding protein, heart | -0.10 | 0.473206 |
| Q9WVH9 | Fibulin-5 | -0.05 | 0.474628 |
| O89079 | Coatomer subunit epsilon | 0.06 | 0.47525 |
| Q91WK2 | Eukaryotic translation initiation factor 3 subunit H | 0.12 | 0.4767 |
| Q9Z0V7 | Mitochondrial import inner membrane translocase subunit Tim17-B | 0.09 | 0.47838 |
| Q99KK7 | Dipeptidyl peptidase 3 | 0.31 | 0.478943 |
| P63325 | 40S ribosomal protein S10 | -0.05 | 0.479236 |
| P61164 | Alpha-centractin | 0.05 | 0.480394 |
| Q59J78 | Mimitin, mitochondrial | 0.67 | 0.481216 |
| P35282 | Ras-related protein Rab-21 | -0.07 | 0.48162 |
| P68040 | Guanine nucleotide-binding protein subunit beta-2-like 1;Guanine nucleotide-binding protein subunit beta-2-like 1, N-terminally processed | -0.05 | 0.481749 |
| O08547 | Vesicle-trafficking protein SEC22b | -0.05 | 0.482592 |
| P43275 | Histone H1.1 | 0.10 | 0.483231 |
| Q07113 | Cation-independent mannose-6-phosphate receptor | -0.14 | 0.484186 |
| P62830 | 60S ribosomal protein L23 | -0.06 | 0.484808 |
| Q9CPQ8 | ATP synthase subunit g, mitochondrial | 0.06 | 0.485127 |
| Q9CR56 | NF-kappa-B inhibitor-interacting Ras-like protein 2 | -0.06 | 0.486509 |
| Q9JJZ2 | Tubulin alpha-8 chain | 0.07 | 0.488213 |
| O55003 | BCL2/adenovirus E1B 19 kDa protein-interacting protein 3 | -0.10 | 0.488819 |
| Q8CJG1 | Protein argonaute-1;Protein argonaute-2;Protein argonaute-4 | 0.10 | 0.490977 |
| F6ZDS4 | Nucleoprotein TPR | 0.05 | 0.491647 |
| Q9WUM4 | Coronin-1C | 0.13 | 0.491702 |
| Q8BRT1 | CLIP-associating protein 2 | 0.07 | 0.49191 |

| | | | |
|---|---|---|---|
| Q80YC5 | Coagulation factor XII;Coagulation factor XIIa heavy chain;Coagulation factor XIIa light chain | 0.12 | 0.493006 |
| Q8C7B8 | Zinc finger SWIM domain-containing protein 4 | -0.13 | 0.493118 |
| Q9EPE9 | Manganese-transporting ATPase 13A1 | 0.08 | 0.493557 |
| Q8CH25 | SAFB-like transcription modulator | 0.12 | 0.494084 |
| P80313 | T-complex protein 1 subunit eta | 0.04 | 0.49536 |
| Q8CEE7 | Retinol dehydrogenase 13 | 0.05 | 0.495783 |
| Q8BGU5 | Cyclin-Y | -0.12 | 0.496022 |
| Q9QXX4 | Calcium-binding mitochondrial carrier protein Aralar2 | -0.05 | 0.496159 |
| P28352 | DNA-(apurinic or apyrimidinic site) lyase;DNA-(apurinic or apyrimidinic site) lyase, mitochondrial | -0.11 | 0.497438 |
| Q8K124 | Pleckstrin homology domain-containing family O member 2 | 0.08 | 0.497939 |
| P60766 | Cell division control protein 42 homolog | 0.05 | 0.498378 |
| Q80SZ7 | Guanine nucleotide-binding protein G(I)/G(S)/G(O) subunit gamma-5 | 0.04 | 0.499352 |
| Q91YE5 | Bromodomain adjacent to zinc finger domain protein 2A | -0.16 | 0.499908 |
| Q3UMF0 | Cordon-bleu protein-like 1 | -0.14 | 0.5004 |
| D3YYU8 | Obscurin-like protein 1 | 0.11 | 0.500655 |
| Q3U186 | Probable arginine--tRNA ligase, mitochondrial | 0.15 | 0.501194 |
| Q9CQZ6 | NADH dehydrogenase [ubiquinone] 1 beta subcomplex subunit 3 | -0.06 | 0.501766 |
| Q7TMM9 | Tubulin beta-2A chain;Tubulin beta-2B chain | 0.06 | 0.502841 |
| Q8WTY4 | Anamorsin | -0.08 | 0.50361 |
| Q9CRA7 | ATP synthase subunit s, mitochondrial | -0.12 | 0.504506 |
| Q99KF1 | Transmembrane emp24 domain-containing protein 9 | 0.14 | 0.504734 |
| E9Q5C9 | Nucleolar and coiled-body phosphoprotein 1 | -0.06 | 0.504938 |
| Q3UBX0 | Transmembrane protein 109 | -0.05 | 0.506142 |
| Q8CHU3 | Epsin-2 | -0.15 | 0.506519 |
| Q8BXV2 | BRI3-binding protein | -0.15 | 0.506703 |
| P63101 | 14-3-3 protein zeta/delta | 0.03 | 0.507111 |
| P47738 | Aldehyde dehydrogenase, mitochondrial | 0.04 | 0.507464 |
| Q8K370 | Acyl-CoA dehydrogenase family member 10 | -0.06 | 0.509496 |
| Q9JJC6 | RILP-like protein 1 | -0.08 | 0.510725 |
| Q7TQD2 | Tubulin polymerization-promoting protein | -0.06 | 0.512389 |
| Q3TLS3 | GDP-D-glucose phosphorylase 1 | 0.07 | 0.513363 |
| P28656 | Nucleosome assembly protein 1-like 1 | 0.09 | 0.513425 |
| Q8C0L0 | Thioredoxin-related transmembrane protein 4 | -0.06 | 0.514936 |
| Q8K2H2 | OTU domain-containing protein 6B | 0.05 | 0.515109 |
| P70265 | 6-phosphofructo-2-kinase/fructose-2,6-bisphosphatase 2;6-phosphofructo-2-kinase;Fructose-2,6-bisphosphatase | -0.06 | 0.51551 |
| P05201 | Aspartate aminotransferase, cytoplasmic | 0.03 | 0.516092 |
| Q8R146 | Acylamino-acid-releasing enzyme | -0.04 | 0.517235 |

| | | | |
|---|---|---|---|
| Q8BYK6 | YTH domain-containing family protein 3;YTH domain-containing family protein 1 | -0.13 | 0.518324 |
| P62196 | 26S protease regulatory subunit 8 | -0.04 | 0.519656 |
| Q6NZC7 | SEC23-interacting protein | 0.07 | 0.520602 |
| P10637 | Microtubule-associated protein tau | -0.04 | 0.521274 |
| Q9CR57 | 60S ribosomal protein L14 | 0.05 | 0.521754 |
| Q9DCD0 | 6-phosphogluconate dehydrogenase, decarboxylating | 0.05 | 0.521923 |
| Q8JZQ9 | Eukaryotic translation initiation factor 3 subunit B | 0.05 | 0.522063 |
| O70250 | Phosphoglycerate mutase 2 | -0.03 | 0.52521 |
| P48725 | Pericentrin | -0.22 | 0.525869 |
| O35382 | Exocyst complex component 4 | 0.08 | 0.52612 |
| Q9CQB5 | CDGSH iron-sulfur domain-containing protein 2 | -0.08 | 0.526609 |
| Q99020 | Heterogeneous nuclear ribonucleoprotein A/B | -0.11 | 0.526776 |
| P10630 | Eukaryotic initiation factor 4A-II;Eukaryotic initiation factor 4A-II, N-terminally processed | -0.14 | 0.526804 |
| P47802 | Metaxin-1 | 0.07 | 0.526934 |
| Q9D1P0 | 39S ribosomal protein L13, mitochondrial | -0.06 | 0.527357 |
| Q80UM3 | N-alpha-acetyltransferase 15, NatA auxiliary subunit | 0.06 | 0.527464 |
| Q8C0C7 | Phenylalanine--tRNA ligase alpha subunit | 0.05 | 0.528107 |
| Q9D358 | Low molecular weight phosphotyrosine protein phosphatase | 0.05 | 0.53057 |
| Q9D7V1 | SH2 domain-containing protein 4A | 0.09 | 0.53164 |
| Q99JW4 | LIM and senescent cell antigen-like-containing domain protein 1 | 0.03 | 0.532589 |
| Q921C5 | Protein bicaudal D homolog 2 | 0.08 | 0.5337 |
| Q61387 | Cytochrome c oxidase subunit 7A-related protein, mitochondrial | -0.10 | 0.534006 |
| Q8CG72 | Poly(ADP-ribose) glycohydrolase ARH3 | 0.04 | 0.534132 |
| Q8BHG1 | Nardilysin | -0.11 | 0.534663 |
| Q8VBT0 | Thioredoxin-related transmembrane protein 1 | 0.22 | 0.536634 |
| Q91WD5 | NADH dehydrogenase [ubiquinone] iron-sulfur protein 2, mitochondrial | 0.04 | 0.536738 |
| P27048 | Small nuclear ribonucleoprotein-associated protein B;Small nuclear ribonucleoprotein-associated protein N | 0.25 | 0.538246 |
| P14152 | Malate dehydrogenase, cytoplasmic | 0.04 | 0.53839 |
| Q61581 | Insulin-like growth factor-binding protein 7 | -0.06 | 0.538495 |
| P17182 | Alpha-enolase | 0.05 | 0.538952 |
| Q9DB15 | 39S ribosomal protein L12, mitochondrial | 0.06 | 0.539006 |
| Q8R001 | Microtubule-associated protein RP/EB family member 2 | -0.06 | 0.539518 |
| Q01149 | Collagen alpha-2(I) chain | 0.10 | 0.53994 |
| Q9JMG1 | Endothelial differentiation-related factor 1 | 0.08 | 0.540925 |
| Q8R127 | Saccharopine dehydrogenase-like oxidoreductase | -0.04 | 0.541871 |
| P37804 | Transgelin | 0.15 | 0.542397 |

| | | | |
|---|---|---|---|
| Q80X82 | Symplekin | 0.08 | 0.542646 |
| P53702 | Cytochrome c-type heme lyase | 0.04 | 0.543124 |
| Q9R269 | Periplakin | 0.31 | 0.543296 |
| P56528 | ADP-ribosyl cyclase/cyclic ADP-ribose hydrolase 1 | 0.16 | 0.543379 |
| Q9CPY1 | 39S ribosomal protein L51, mitochondrial | -0.06 | 0.543831 |
| P49138 | MAP kinase-activated protein kinase 2 | -0.06 | 0.54437 |
| Q8JZS9 | 39S ribosomal protein L48, mitochondrial | 0.04 | 0.544581 |
| O09044 | Synaptosomal-associated protein 23 | -0.15 | 0.54495 |
| Q6IRU2 | Tropomyosin alpha-4 chain | 0.04 | 0.544982 |
| Q9D113 | DNL-type zinc finger protein | 0.04 | 0.546791 |
| Q9JLN9 | Serine/threonine-protein kinase mTOR | -0.06 | 0.546918 |
| Q61703 | Inter-alpha-trypsin inhibitor heavy chain H2 | -0.06 | 0.548521 |
| P32507 | Nectin-2 | 0.12 | 0.550283 |
| O08553 | Dihydropyrimidinase-related protein 2 | -0.04 | 0.550311 |
| Q8C3X4 | Translation factor Guf1, mitochondrial | -0.05 | 0.551107 |
| P47911 | 60S ribosomal protein L6 | -0.06 | 0.552045 |
| Q920A7 | AFG3-like protein 1 | -0.08 | 0.552344 |
| Q9DAW9 | Calponin-3 | -0.04 | 0.552506 |
| P99024 | Tubulin beta-5 chain | 0.04 | 0.553489 |
| P08122 | Collagen alpha-2(IV) chain;Canstatin | -0.06 | 0.554264 |
| Q4ACU6 | SH3 and multiple ankyrin repeat domains protein 3 | -0.19 | 0.554403 |
| P97443 | Histone-lysine N-methyltransferase Smyd1 | 0.05 | 0.556271 |
| Q9WV54 | Acid ceramidase;Acid ceramidase subunit alpha;Acid ceramidase subunit beta | -0.07 | 0.556357 |
| Q8BX02 | KN motif and ankyrin repeat domain-containing protein 2 | 0.04 | 0.558694 |
| Q9CY27 | Very-long-chain enoyl-CoA reductase | 0.06 | 0.558726 |
| P28654 | Decorin | 0.05 | 0.560907 |
| P14115 | 60S ribosomal protein L27a | 0.16 | 0.562551 |
| Q9CY58 | Plasminogen activator inhibitor 1 RNA-binding protein | 0.12 | 0.563791 |
| Q8JZQ2 | AFG3-like protein 2 | 0.03 | 0.564574 |
| Q8BFY9 | Transportin-1;Transportin-2 | 0.04 | 0.565043 |
| Q9D819 | Inorganic pyrophosphatase | 0.04 | 0.56516 |
| P20152 | Vimentin | -0.04 | 0.56703 |
| Q91YT0 | NADH dehydrogenase [ubiquinone] flavoprotein 1, mitochondrial | -0.06 | 0.568828 |
| Q9CRB9 | MICOS complex subunit Mic19 | 0.08 | 0.569605 |
| Q91WQ3 | Tyrosine--tRNA ligase, cytoplasmic;Tyrosine--tRNA ligase, cytoplasmic, N-terminally processed | 0.07 | 0.57112 |
| P36993 | Protein phosphatase 1B | -0.05 | 0.571637 |
| P70271 | PDZ and LIM domain protein 4 | 0.08 | 0.571639 |
| Q6ZWV7 | 60S ribosomal protein L35 | 0.17 | 0.57167 |
| Q9JKB1 | Ubiquitin carboxyl-terminal hydrolase isozyme L3;Ubiquitin carboxyl-terminal hydrolase isozyme L4 | 0.12 | 0.572346 |

| | | | |
|---|---|---|---|
| P01942 | Hemoglobin subunit alpha | -0.06 | 0.574202 |
| P68368 | Tubulin alpha-4A chain | 0.03 | 0.574717 |
| P24549 | Retinal dehydrogenase 1 | -0.07 | 0.575564 |
| P70670 | Nascent polypeptide-associated complex subunit alpha, muscle-specific form | -0.05 | 0.575811 |
| Q8CI32 | BAG family molecular chaperone regulator 5 | -0.09 | 0.576481 |
| P84091 | AP-2 complex subunit mu | -0.04 | 0.576704 |
| Q3UX61 | N-alpha-acetyltransferase 11;N-alpha-acetyltransferase 10 | 0.07 | 0.582607 |
| Q9D1J1 | Adaptin ear-binding coat-associated protein 2 | -0.15 | 0.582751 |
| P56379 | 6.8 kDa mitochondrial proteolipid | 0.04 | 0.583053 |
| P48771 | Cytochrome c oxidase subunit 7A2, mitochondrial | -0.06 | 0.585104 |
| Q8BI84 | Melanoma inhibitory activity protein 3 | 0.13 | 0.58556 |
| Q924T2 | 28S ribosomal protein S2, mitochondrial | 0.11 | 0.585632 |
| Q8BHD7 | Polypyrimidine tract-binding protein 3 | -0.09 | 0.585735 |
| Q9JM76 | Actin-related protein 2/3 complex subunit 3 | -0.06 | 0.586154 |
| Q63844 | Mitogen-activated protein kinase 3 | 0.06 | 0.587824 |
| P27005 | Protein S100-A8 | 0.14 | 0.588019 |
| Q8CH40 | Nucleoside diphosphate-linked moiety X motif 6 | -0.11 | 0.588565 |
| Q922Q8 | Leucine-rich repeat-containing protein 59 | -0.07 | 0.589401 |
| P06745 | Glucose-6-phosphate isomerase | -0.06 | 0.589423 |
| Q9QYG0 | Protein NDRG2 | 0.04 | 0.590013 |
| P48774 | Glutathione S-transferase Mu 5 | -0.04 | 0.590197 |
| O35386 | Phytanoyl-CoA dioxygenase, peroxisomal | 0.05 | 0.590309 |
| Q5FW52 | Muscular LMNA-interacting protein | -0.03 | 0.59075 |
| Q8BZA9 | Fructose-2,6-bisphosphatase TIGAR | 0.04 | 0.591936 |
| P22907 | Porphobilinogen deaminase | -0.06 | 0.59203 |
| Q3TC72 | Fumarylacetoacetate hydrolase domain-containing protein 2A | 0.05 | 0.592381 |
| P59325 | Eukaryotic translation initiation factor 5 | -0.03 | 0.595229 |
| Q99JY9 | Actin-related protein 3 | -0.11 | 0.595968 |
| Q3TC46 | Protein PAT1 homolog 1 | -0.17 | 0.596049 |
| O09106 | Histone deacetylase 1;Histone deacetylase 2 | 0.07 | 0.596385 |
| Q8C1B7 | Septin-11 | -0.08 | 0.596546 |
| Q99PT1 | Rho GDP-dissociation inhibitor 1 | -0.04 | 0.596602 |
| Q91VN4 | MICOS complex subunit Mic25 | -0.07 | 0.597234 |
| Q8BGC0 | HIV Tat-specific factor 1 homolog | -0.05 | 0.597347 |
| Q9JLJ2 | 4-trimethylaminobutyraldehyde dehydrogenase | -0.04 | 0.598678 |
| Q9D1G5 | Leucine-rich repeat-containing protein 57 | -0.05 | 0.598974 |
| P09411 | Phosphoglycerate kinase 1 | 0.03 | 0.599177 |
| Q920E5 | Farnesyl pyrophosphate synthase | 0.18 | 0.599297 |
| Q9D880 | Mitochondrial import inner membrane translocase subunit TIM50 | 0.09 | 0.599341 |

| | | | |
|---|---|---|---|
| Q571I9 | Aldehyde dehydrogenase family 16 member A1 | 0.13 | 0.601984 |
| Q9DC77 | Small muscular protein | -0.03 | 0.602762 |
| Q9DBS5 | Kinesin light chain 4 | -0.07 | 0.603012 |
| P18760 | Cofilin-1 | 0.04 | 0.603385 |
| P62267 | 40S ribosomal protein S23 | 0.07 | 0.603825 |
| Q9JLQ0 | CD2-associated protein | -0.10 | 0.60493 |
| G5E8K5 | Ankyrin-3 | 0.08 | 0.605012 |
| Q9Z2U0 | Proteasome subunit alpha type-7;Proteasome subunit alpha type-7-like | -0.08 | 0.60657 |
| Q9ER35 | Fructosamine-3-kinase | -0.04 | 0.606708 |
| Q9DCZ1 | GMP reductase 1 | -0.06 | 0.607286 |
| P46938 | Transcriptional coactivator YAP1 | 0.06 | 0.607795 |
| Q9D1I5 | Methylmalonyl-CoA epimerase, mitochondrial | 0.03 | 0.608035 |
| Q9CXS4 | Centromere protein V | 0.03 | 0.608241 |
| Q9QY76 | Vesicle-associated membrane protein-associated protein B | 0.06 | 0.608332 |
| Q91Z49 | UAP56-interacting factor | -0.08 | 0.609048 |
| Q9EQI8 | 39S ribosomal protein L46, mitochondrial | 0.05 | 0.609144 |
| P46062 | Signal-induced proliferation-associated protein 1 | 0.08 | 0.610218 |
| Q8K199 | COX assembly mitochondrial protein 2 homolog | 0.05 | 0.61138 |
| Q52KI8 | Serine/arginine repetitive matrix protein 1 | 0.08 | 0.611868 |
| Q8BH64 | EH domain-containing protein 2 | -0.03 | 0.612125 |
| O54724 | Polymerase I and transcript release factor | 0.06 | 0.612159 |
| O55234 | Proteasome subunit beta type-5 | 0.04 | 0.612813 |
| Q9QWW1 | Homer protein homolog 2 | -0.05 | 0.613594 |
| P24472 | Glutathione S-transferase A4 | -0.06 | 0.615067 |
| Q8K274 | Ketosamine-3-kinase | -0.07 | 0.616555 |
| Q8BSZ2 | AP-3 complex subunit sigma-2 | -0.06 | 0.618033 |
| Q9QWK4 | CD5 antigen-like | -0.07 | 0.61842 |
| Q9JI39 | ATP-binding cassette sub-family B member 10, mitochondrial | 0.04 | 0.618624 |
| P05213 | Tubulin alpha-1B chain | -0.03 | 0.61871 |
| Q60766 | Immunity-related GTPase family M protein 1 | 0.07 | 0.618819 |
| P62897 | Cytochrome c, somatic | -0.07 | 0.619515 |
| P68181 | cAMP-dependent protein kinase catalytic subunit beta | -0.28 | 0.619927 |
| Q9D0G0 | 28S ribosomal protein S30, mitochondrial | 0.04 | 0.620049 |
| Q8R1Q9 | Ribokinase | -0.05 | 0.62043 |
| O08638 | Myosin-11 | 0.04 | 0.622185 |
| Q5SSK3 | Transcription elongation factor, mitochondrial | 0.06 | 0.624285 |
| Q8CCK0 | Core histone macro-H2A.2 | 0.08 | 0.624628 |
| P09405 | Nucleolin | -0.06 | 0.626718 |
| Q00560 | Interleukin-6 receptor subunit beta | 0.06 | 0.627884 |
| Q5EBG8 | Uncharacterized protein C1orf50 homolog | -0.10 | 0.629012 |

| | | | |
|---|---|---|---|
| P53395 | Lipoamide acyltransferase component of branched-chain alpha-keto acid dehydrogenase complex, mitochondrial | 0.03 | 0.629086 |
| Q8R570 | Synaptosomal-associated protein 47 | -0.12 | 0.629976 |
| P97927 | Laminin subunit alpha-4 | 0.04 | 0.630183 |
| A2A5R2 | Brefeldin A-inhibited guanine nucleotide-exchange protein 2 | 0.06 | 0.630985 |
| Q9CVB6 | Actin-related protein 2/3 complex subunit 2 | -0.04 | 0.631427 |
| Q9D7S9 | Charged multivesicular body protein 5 | -0.05 | 0.633355 |
| Q8CI95 | Oxysterol-binding protein-related protein 11 | 0.09 | 0.633568 |
| Q8C1Q6 | Small integral membrane protein 4 | 0.05 | 0.63428 |
| Q8R550 | SH3 domain-containing kinase-binding protein 1 | 0.03 | 0.634513 |
| P60824 | Cold-inducible RNA-binding protein | 0.05 | 0.635248 |
| P0C7L0 | WAS/WASL-interacting protein family member 3 | -0.03 | 0.635559 |
| Q05D44 | Eukaryotic translation initiation factor 5B | 0.04 | 0.63582 |
| Q8BJ71 | Nuclear pore complex protein Nup93 | -0.11 | 0.636096 |
| Q8K0Z7 | Translational activator of cytochrome c oxidase 1 | 0.04 | 0.636511 |
| P62702 | 40S ribosomal protein S4, X isoform | 0.10 | 0.636636 |
| P16858 | Glyceraldehyde-3-phosphate dehydrogenase | 0.02 | 0.639222 |
| Q9D0I4 | Syntaxin-17 | 0.07 | 0.639463 |
| P0DJE0 | Protein PET100 homolog, mitochondrial | 0.06 | 0.639525 |
| P48787 | Troponin I, cardiac muscle | -0.03 | 0.639771 |
| Q8VCT4 | Carboxylesterase 1D | -0.04 | 0.640674 |
| Q9R061 | Cytosolic Fe-S cluster assembly factor NUBP2 | 0.08 | 0.640814 |
| Q8VH51 | RNA-binding protein 39 | 0.04 | 0.641354 |
| Q9D1L0 | Coiled-coil-helix-coiled-coil-helix domain-containing protein 2 | -0.03 | 0.641904 |
| Q99N89 | 39S ribosomal protein L43, mitochondrial | 0.04 | 0.642051 |
| Q9CQE8 | UPF0568 protein C14orf166 homolog | -0.04 | 0.642412 |
| Q8K1L5 | Protein phosphatase 1 regulatory subunit 11 | 0.14 | 0.643357 |
| Q6A068 | Cell division cycle 5-like protein | -0.04 | 0.643556 |
| Q8R3Q6 | Coiled-coil domain-containing protein 58 | -0.04 | 0.643654 |
| P61021 | Ras-related protein Rab-5B | -0.10 | 0.644486 |
| O70492 | Sorting nexin-3 | 0.10 | 0.644876 |
| Q9EQP2 | EH domain-containing protein 4 | -0.03 | 0.647204 |
| P08003 | Protein disulfide-isomerase A4 | 0.06 | 0.648533 |
| O09164 | Extracellular superoxide dismutase [Cu-Zn] | -0.07 | 0.648847 |
| P06684 | Complement C5;Complement C5 beta chain;Complement C5 alpha chain;C5a anaphylatoxin;Complement C5 alpha chain | -0.06 | 0.649552 |
| Q8BZF8 | Phosphoglucomutase-like protein 5 | 0.04 | 0.650196 |
| Q9CPU4 | Microsomal glutathione S-transferase 3 | 0.03 | 0.650265 |
| Q8K4G5 | Actin-binding LIM protein 1 | 0.03 | 0.65041 |
| Q9R000 | Integrin beta-1-binding protein 2 | 0.03 | 0.651749 |

| | | | |
|---|---|---|---|
| O35129 | Prohibitin-2 | 0.02 | 0.65268 |
| P27612 | Phospholipase A-2-activating protein | -0.03 | 0.654296 |
| P62077 | Mitochondrial import inner membrane translocase subunit Tim8 B | 0.04 | 0.654603 |
| Q9CQN1 | Heat shock protein 75 kDa, mitochondrial | 0.03 | 0.654849 |
| Q3UIJ9 | Myocardial zonula adherens protein | -0.03 | 0.655374 |
| Q9CQ69 | Cytochrome b-c1 complex subunit 8 | 0.02 | 0.655515 |
| Q8R361 | Rab11 family-interacting protein 5 | -0.03 | 0.65589 |
| Q5XPI3 | E3 ubiquitin-protein ligase RNF123 | 0.05 | 0.656448 |
| P10711 | Transcription elongation factor A protein 1 | 0.05 | 0.657458 |
| P46935 | E3 ubiquitin-protein ligase NEDD4 | 0.04 | 0.658012 |
| Q9R0P9 | Ubiquitin carboxyl-terminal hydrolase isozyme L1 | 0.04 | 0.658487 |
| Q11011 | Puromycin-sensitive aminopeptidase | 0.03 | 0.659405 |
| Q99KR7 | Peptidyl-prolyl cis-trans isomerase F, mitochondrial | -0.04 | 0.659547 |
| Q8VCR7 | Alpha/beta hydrolase domain-containing protein 14B | 0.07 | 0.659752 |
| Q9CQA3 | Succinate dehydrogenase [ubiquinone] iron-sulfur subunit, mitochondrial | 0.04 | 0.660203 |
| Q8CGE8 | Interferon-activable protein 205-A | 0.08 | 0.661765 |
| Q61586 | Glycerol-3-phosphate acyltransferase 1, mitochondrial | 0.08 | 0.662063 |
| Q7TMY8 | E3 ubiquitin-protein ligase HUWE1 | 0.05 | 0.662893 |
| Q9Z1E4 | Glycogen [starch] synthase, muscle | -0.03 | 0.66295 |
| Q8C7X2 | ER membrane protein complex subunit 1 | 0.07 | 0.66317 |
| Q99KJ0 | UBX domain-containing protein 2A | 0.07 | 0.663992 |
| P84102 | Small EDRK-rich factor 2 | -0.05 | 0.664424 |
| Q9R0P5 | Destrin | -0.06 | 0.664451 |
| Q52KR3 | Protein prune homolog 2 | 0.05 | 0.664902 |
| Q921G7 | Electron transfer flavoprotein-ubiquinone oxidoreductase, mitochondrial | -0.04 | 0.666432 |
| P84089 | Enhancer of rudimentary homolog | -0.05 | 0.666473 |
| Q8R1B4 | Eukaryotic translation initiation factor 3 subunit C | -0.03 | 0.666844 |
| Q8K1B8 | Fermitin family homolog 3 | -0.05 | 0.667049 |
| Q9EQU5 | Protein SET | -0.07 | 0.668128 |
| Q8C0L9 | Glycerophosphocholine phosphodiesterase GPCPD1 | -0.02 | 0.6705 |
| Q9CQD1 | Ras-related protein Rab-5A | 0.04 | 0.670929 |
| P80316 | T-complex protein 1 subunit epsilon | -0.03 | 0.671615 |
| P54923 | [Protein ADP-ribosylarginine] hydrolase | 0.07 | 0.672444 |
| Q9JHS4 | ATP-dependent Clp protease ATP-binding subunit clpX-like, mitochondrial | 0.03 | 0.672589 |
| Q9CZM2 | 60S ribosomal protein L15 | 0.05 | 0.672877 |
| Q9D0L7 | Armadillo repeat-containing protein 10 | 0.07 | 0.674779 |
| Q9CR76 | Transmembrane protein 186 | -0.04 | 0.674873 |
| P32261 | Antithrombin-III | -0.04 | 0.674944 |
| Q9Z239 | Phospholemman | -0.14 | 0.675858 |

| | | | |
|---|---|---|---|
| Q9R0P4 | Small acidic protein | 0.05 | 0.677564 |
| P35123 | Ubiquitin carboxyl-terminal hydrolase 4 | 0.10 | 0.677918 |
| Q8C0M9 | Isoaspartyl peptidase/L-asparaginase;Isoaspartyl peptidase/L-asparaginase alpha chain;Isoaspartyl peptidase/L-asparaginase beta chain | 0.06 | 0.680061 |
| P70290 | 55 kDa erythrocyte membrane protein | 0.03 | 0.681288 |
| P62858 | 40S ribosomal protein S28 | -0.03 | 0.681738 |
| Q8K0E8 | Fibrinogen beta chain;Fibrinopeptide B;Fibrinogen beta chain | 0.04 | 0.682584 |
| O70571 | [Pyruvate dehydrogenase (acetyl-transferring)] kinase isozyme 4, mitochondrial | -0.05 | 0.683379 |
| Q8BH61 | Coagulation factor XIII A chain | -0.05 | 0.68402 |
| Q8BTY8 | Sec1 family domain-containing protein 2 | -0.03 | 0.687309 |
| Q9R1P4 | Proteasome subunit alpha type-1 | -0.05 | 0.687808 |
| Q9D5T0 | ATPase family AAA domain-containing protein 1 | 0.04 | 0.690902 |
| P62073 | Mitochondrial import inner membrane translocase subunit Tim10 | -0.04 | 0.690991 |
| O08997 | Copper transport protein ATOX1 | 0.03 | 0.692723 |
| Q78IK2 | Up-regulated during skeletal muscle growth protein 5 | -0.07 | 0.693231 |
| Q9CQ65 | S-methyl-5-thioadenosine phosphorylase | 0.02 | 0.694533 |
| Q9EPB5 | Serine hydrolase-like protein | -0.03 | 0.694717 |
| Q99JP6 | Homer protein homolog 3 | -0.09 | 0.696908 |
| Q9JK42 | [Pyruvate dehydrogenase (acetyl-transferring)] kinase isozyme 2, mitochondrial | 0.04 | 0.699362 |
| Q64727 | Vinculin | 0.03 | 0.700158 |
| Q9Z0N1 | Eukaryotic translation initiation factor 2 subunit 3, X-linked | 0.03 | 0.701712 |
| Q64511 | DNA topoisomerase 2-beta | 0.06 | 0.702116 |
| O35326 | Serine/arginine-rich splicing factor 5 | -0.03 | 0.702301 |
| P35979 | 60S ribosomal protein L12 | -0.02 | 0.703734 |
| Q9JI75 | Ribosyldihydronicotinamide dehydrogenase [quinone] | 0.07 | 0.704746 |
| P34884 | Macrophage migration inhibitory factor | -0.03 | 0.705092 |
| Q6PB66 | Leucine-rich PPR motif-containing protein, mitochondrial | 0.04 | 0.707557 |
| P01887 | Beta-2-microglobulin | -0.04 | 0.709057 |
| Q8VCI5 | Peroxisomal biogenesis factor 19 | -0.06 | 0.709461 |
| Q05BC3 | Echinoderm microtubule-associated protein-like 1 | 0.04 | 0.709776 |
| Q99NB9 | Splicing factor 3B subunit 1 | 0.05 | 0.712551 |
| Q05816 | Fatty acid-binding protein, epidermal | 0.04 | 0.712986 |
| O88696 | ATP-dependent Clp protease proteolytic subunit, mitochondrial | 0.03 | 0.713485 |
| Q8VDI7 | Ubiquitin-associated domain-containing protein 1 | -0.05 | 0.71463 |
| Q8VCX5 | Calcium uptake protein 1, mitochondrial | -0.07 | 0.714746 |

| | | | |
|---|---|---|---|
| P56394 | Cytochrome c oxidase copper chaperone | -0.03 | 0.716917 |
| Q9DAV9 | Trimeric intracellular cation channel type B | -0.07 | 0.717437 |
| Q9CQ06 | 39S ribosomal protein L24, mitochondrial | 0.03 | 0.717953 |
| Q9D0E1 | Heterogeneous nuclear ribonucleoprotein M | 0.02 | 0.719243 |
| P97352 | Protein S100-A13 | -0.06 | 0.719527 |
| Q8K4F5 | Alpha/beta hydrolase domain-containing protein 11 | -0.03 | 0.719871 |
| Q9D7B6 | Isobutyryl-CoA dehydrogenase, mitochondrial | 0.03 | 0.720454 |
| Q99J99 | 3-mercaptopyruvate sulfurtransferase | -0.05 | 0.720585 |
| Q8BW75 | Amine oxidase [flavin-containing] B | 0.02 | 0.721001 |
| Q00897 | Alpha-1-antitrypsin 1-4 | 0.02 | 0.721729 |
| P21550 | Beta-enolase | 0.02 | 0.723593 |
| Q91VM9 | Inorganic pyrophosphatase 2, mitochondrial | -0.03 | 0.726527 |
| Q9QX60 | Deoxyguanosine kinase, mitochondrial | -0.11 | 0.728701 |
| Q80XB4 | Nebulin-related-anchoring protein | -0.02 | 0.730094 |
| Q9CR98 | Protein FAM136A | -0.03 | 0.730278 |
| Q61129 | Complement factor I;Complement factor I heavy chain;Complement factor I light chain | 0.02 | 0.730384 |
| Q8C1A5 | Thimet oligopeptidase | 0.04 | 0.731138 |
| Q7TMK9 | Heterogeneous nuclear ribonucleoprotein Q | 0.02 | 0.731194 |
| Q3URS9 | Coiled-coil domain-containing protein 51 | -0.03 | 0.734078 |
| Q99KN9 | Clathrin interactor 1 | -0.05 | 0.734121 |
| Q7TQI3 | Ubiquitin thioesterase OTUB1 | -0.02 | 0.738435 |
| P15626 | Glutathione S-transferase Mu 2 | -0.03 | 0.738816 |
| Q99JI4 | 26S proteasome non-ATPase regulatory subunit 6 | 0.04 | 0.741534 |
| P10649 | Glutathione S-transferase Mu 1 | 0.02 | 0.742199 |
| P35278 | Ras-related protein Rab-5C | 0.03 | 0.743127 |
| P03893 | NADH-ubiquinone oxidoreductase chain 2 | -0.04 | 0.743599 |
| Q9JJL8 | Serine--tRNA ligase, mitochondrial | 0.03 | 0.745645 |
| Q9QZQ1 | Afadin | -0.02 | 0.746332 |
| Q9D6Y9 | 1,4-alpha-glucan-branching enzyme | -0.03 | 0.747771 |
| Q61655 | ATP-dependent RNA helicase DDX19A | -0.04 | 0.748045 |
| P23927 | Alpha-crystallin B chain | -0.04 | 0.749344 |
| Q9D385 | ADP-ribosylation factor-like protein 2-binding protein | 0.08 | 0.749519 |
| Q9CXV1 | Succinate dehydrogenase [ubiquinone] cytochrome b small subunit, mitochondrial | -0.10 | 0.749754 |
| Q9Z2X2 | 26S proteasome non-ATPase regulatory subunit 10 | 0.06 | 0.749808 |
| O89020 | Afamin | 0.02 | 0.750271 |
| P54227 | Stathmin | 0.04 | 0.75206 |
| Q8CAS9 | Poly [ADP-ribose] polymerase 9 | -0.06 | 0.752305 |
| Q8BSK8 | Ribosomal protein S6 kinase beta-1 | 0.05 | 0.753023 |
| Q7M6Y3 | Phosphatidylinositol-binding clathrin assembly protein | -0.02 | 0.753186 |
| Q6ZQB6 | Inositol hexakisphosphate and diphosphoinositol-pentakisphosphate kinase 2 | -0.03 | 0.753761 |

| | | | |
|---|---|---|---|
| Q02780 | Nuclear factor 1 A-type | -0.07 | 0.755517 |
| P62334 | 26S protease regulatory subunit 10B | 0.02 | 0.756341 |
| Q9Z266 | SNARE-associated protein Snapin | -0.07 | 0.756557 |
| P18654 | Ribosomal protein S6 kinase alpha-3 | 0.02 | 0.756816 |
| Q5HZI9 | Solute carrier family 25 member 51 | -0.09 | 0.758116 |
| P27546 | Microtubule-associated protein 4 | -0.02 | 0.758766 |
| P59999 | Actin-related protein 2/3 complex subunit 4 | 0.02 | 0.75988 |
| P59016 | Vacuolar protein sorting-associated protein 33B | 0.03 | 0.760135 |
| Q8CGK3 | Lon protease homolog, mitochondrial | -0.02 | 0.761359 |
| O70439 | Syntaxin-7 | 0.05 | 0.761766 |
| P11438 | Lysosome-associated membrane glycoprotein 1 | 0.02 | 0.762864 |
| Q3THW5 | Histone H2A.V;Histone H2A.Z | -0.03 | 0.764114 |
| Q64433 | 10 kDa heat shock protein, mitochondrial | -0.03 | 0.764412 |
| Q62234 | Myomesin-1 | 0.02 | 0.764862 |
| Q8R4N0 | Citrate lyase subunit beta-like protein, mitochondrial | -0.03 | 0.765262 |
| Q91YJ5 | Translation initiation factor IF-2, mitochondrial | 0.02 | 0.765342 |
| Q9CPU2 | NADH dehydrogenase [ubiquinone] 1 beta subcomplex subunit 2, mitochondrial | -0.06 | 0.765815 |
| Q9D1N2 | Glycosylphosphatidylinositol-anchored high density lipoprotein-binding protein 1 | -0.04 | 0.76608 |
| Q9WUM5 | Succinyl-CoA ligase [ADP/GDP-forming] subunit alpha, mitochondrial | 0.02 | 0.767418 |
| P10852 | 4F2 cell-surface antigen heavy chain | -0.04 | 0.767501 |
| P63158 | High mobility group protein B1 | -0.02 | 0.7677 |
| Q8CIN4 | Serine/threonine-protein kinase PAK 2;PAK-2p27;PAK-2p34 | 0.03 | 0.767898 |
| Q9JLM9 | Growth factor receptor-bound protein 14 | 0.04 | 0.768383 |
| P00405 | Cytochrome c oxidase subunit 2 | -0.02 | 0.769275 |
| Q8BGX2 | Uncharacterized protein C19orf52 homolog | 0.03 | 0.769408 |
| Q6P1F6 | Serine/threonine-protein phosphatase 2A 55 kDa regulatory subunit B alpha isoform | 0.02 | 0.769624 |
| P62301 | 40S ribosomal protein S13 | 0.04 | 0.770234 |
| Q71RI9 | Kynurenine--oxoglutarate transaminase 3 | 0.02 | 0.770337 |
| Q9Z0V8 | Mitochondrial import inner membrane translocase subunit Tim17-A | 0.03 | 0.772252 |
| P31428 | Dipeptidase 1 | -0.03 | 0.772598 |
| Q91ZE0 | Trimethyllysine dioxygenase, mitochondrial | 0.03 | 0.773523 |
| Q80Y14 | Glutaredoxin-related protein 5, mitochondrial | 0.02 | 0.774208 |
| Q9DBP5 | UMP-CMP kinase | 0.02 | 0.774531 |
| Q6PDY2 | 2-aminoethanethiol dioxygenase | -0.02 | 0.774785 |
| Q9DC61 | Mitochondrial-processing peptidase subunit alpha | 0.03 | 0.774929 |
| P09055 | Integrin beta-1 | 0.02 | 0.775264 |
| Q5M8N4 | Epimerase family protein SDR39U1 | 0.02 | 0.775566 |

| | | | |
|---|---|---|---|
| P68369 | Tubulin alpha-1A chain;Tubulin alpha-1C chain;Tubulin alpha-3 chain | 0.03 | 0.776523 |
| O70251 | Elongation factor 1-beta | 0.04 | 0.779448 |
| Q9D6S7 | Ribosome-recycling factor, mitochondrial | 0.03 | 0.780661 |
| P62137 | Serine/threonine-protein phosphatase PP1-alpha catalytic subunit | 0.04 | 0.781362 |
| Q9D6U8 | Protein FAM162A | -0.02 | 0.782115 |
| P58064 | 28S ribosomal protein S6, mitochondrial | 0.05 | 0.782518 |
| P62317 | Small nuclear ribonucleoprotein Sm D2 | 0.04 | 0.783234 |
| Q31125 | Zinc transporter SLC39A7 | 0.03 | 0.783842 |
| P08207 | Protein S100-A10 | -0.04 | 0.784286 |
| P25976 | Nucleolar transcription factor 1 | 0.06 | 0.784485 |
| Q61160 | FAS-associated death domain protein | 0.05 | 0.784766 |
| P20065 | Thymosin beta-4;Hematopoietic system regulatory peptide | 0.20 | 0.784838 |
| Q8VCT3 | Aminopeptidase B | 0.02 | 0.785832 |
| P97822 | Acidic leucine-rich nuclear phosphoprotein 32 family member E | 0.05 | 0.786475 |
| Q9CU62 | Structural maintenance of chromosomes protein 1A | 0.08 | 0.78716 |
| Q9EPK5 | WW domain-containing transcription regulator protein 1 | 0.08 | 0.787922 |
| P97333 | Neuropilin-1 | 0.05 | 0.788878 |
| Q8BLY2 | Probable threonine--tRNA ligase 2, cytoplasmic | 0.14 | 0.789582 |
| Q8R1X6 | Spartin | -0.03 | 0.789904 |
| Q8BP48 | Methionine aminopeptidase 1 | -0.02 | 0.791392 |
| P45376 | Aldose reductase | -0.02 | 0.79141 |
| Q505F5 | Leucine-rich repeat-containing protein 47 | -0.02 | 0.791556 |
| P28271 | Cytoplasmic aconitate hydratase | 0.03 | 0.791798 |
| Q8VCF0 | Mitochondrial antiviral-signaling protein | -0.02 | 0.792253 |
| Q8K2B3 | Succinate dehydrogenase [ubiquinone] flavoprotein subunit, mitochondrial | -0.02 | 0.793728 |
| Q8BGS7 | Choline/ethanolaminephosphotransferase 1 | 0.03 | 0.794132 |
| O70318 | Band 4.1-like protein 2 | -0.02 | 0.794362 |
| Q9D0Q7 | 39S ribosomal protein L45, mitochondrial | 0.02 | 0.795224 |
| Q9CRB6 | Tubulin polymerization-promoting protein family member 3 | 0.02 | 0.795825 |
| Q9D051 | Pyruvate dehydrogenase E1 component subunit beta, mitochondrial | -0.02 | 0.795863 |
| Q9D020 | Cytosolic 5-nucleotidase 3A | 0.02 | 0.796884 |
| Q62348 | Translin | -0.02 | 0.797211 |
| Q8BMJ3 | Eukaryotic translation initiation factor 1A, X-chromosomal;Eukaryotic translation initiation factor 1A | 0.04 | 0.797212 |
| P46656 | Adrenodoxin, mitochondrial | 0.02 | 0.799621 |
| P62869 | Transcription elongation factor B polypeptide 2 | 0.05 | 0.800665 |
| Q9DBR7 | Protein phosphatase 1 regulatory subunit 12A | -0.02 | 0.800705 |

| | | | |
|---|---|---|---|
| Q8R0F8 | Acylpyruvase FAHD1, mitochondrial | 0.02 | 0.801035 |
| Q922B2 | Aspartate--tRNA ligase, cytoplasmic | -0.02 | 0.801784 |
| P28661 | Septin-4 | -0.02 | 0.802126 |
| Q9QX11 | Cytohesin-1;Cytohesin-3;Cytohesin-2 | 0.03 | 0.802429 |
| Q99PV0 | Pre-mRNA-processing-splicing factor 8 | -0.02 | 0.80282 |
| P15864 | Histone H1.2 | 0.02 | 0.803144 |
| P19536 | Cytochrome c oxidase subunit 5B, mitochondrial | -0.03 | 0.803709 |
| P03911 | NADH-ubiquinone oxidoreductase chain 4 | -0.02 | 0.804175 |
| Q91YP2 | Neurolysin, mitochondrial | 0.02 | 0.804297 |
| O70373 | Xin actin-binding repeat-containing protein 1 | 0.02 | 0.804546 |
| P63280 | SUMO-conjugating enzyme UBC9 | 0.05 | 0.807534 |
| Q8K2J0 | 1-phosphatidylinositol 4,5-bisphosphate phosphodiesterase delta-3 | -0.03 | 0.809205 |
| Q9WUX5 | Protein MRVI1 | 0.05 | 0.809276 |
| Q02248 | Catenin beta-1 | -0.02 | 0.81077 |
| P62754 | 40S ribosomal protein S6 | 0.02 | 0.812038 |
| Q8BJS4 | SUN domain-containing protein 2 | -0.02 | 0.812343 |
| P48722 | Heat shock 70 kDa protein 4L | -0.02 | 0.812354 |
| Q9Z2Y3 | Homer protein homolog 1 | -0.04 | 0.812561 |
| Q922U1 | U4/U6 small nuclear ribonucleoprotein Prp3 | 0.05 | 0.812685 |
| P35486 | Pyruvate dehydrogenase E1 component subunit alpha, somatic form, mitochondrial | -0.02 | 0.814987 |
| Q6NXN1 | SUZ domain-containing protein 1 | -0.03 | 0.815 |
| P63085 | Mitogen-activated protein kinase 1 | 0.02 | 0.815215 |
| Q9QYB1 | Chloride intracellular channel protein 4 | -0.01 | 0.815691 |
| Q8R010 | Aminoacyl tRNA synthase complex-interacting multifunctional protein 2 | -0.04 | 0.816211 |
| P10107 | Annexin A1 | -0.02 | 0.816539 |
| Q8R5G7 | Arf-GAP with Rho-GAP domain, ANK repeat and PH domain-containing protein 3 | -0.03 | 0.816958 |
| P61161 | Actin-related protein 2 | 0.01 | 0.818168 |
| Q99N91 | 39S ribosomal protein L34, mitochondrial | 0.02 | 0.82136 |
| Q61554 | Fibrillin-1 | -0.02 | 0.82158 |
| Q9JHR7 | Insulin-degrading enzyme | -0.03 | 0.823152 |
| Q8BVI4 | Dihydropteridine reductase | -0.03 | 0.823525 |
| D3Z7P3 | Glutaminase kidney isoform, mitochondrial | 0.02 | 0.82505 |
| Q8BGC4 | Zinc-binding alcohol dehydrogenase domain-containing protein 2 | 0.02 | 0.825167 |
| P58137 | Acyl-coenzyme A thioesterase 8 | 0.07 | 0.825698 |
| P62855 | 40S ribosomal protein S26 | -0.02 | 0.826401 |
| Q62086 | Serum paraoxonase/arylesterase 2 | 0.03 | 0.826482 |
| Q8VI63 | MOB kinase activator 2 | -0.03 | 0.826743 |
| P29341 | Polyadenylate-binding protein 1 | -0.02 | 0.827169 |

| | | | |
|---|---|---|---|
| Q9Z2Y8 | Proline synthase co-transcribed bacterial homolog protein | 0.01 | 0.827317 |
| Q3UN02 | Lysocardiolipin acyltransferase 1 | 0.02 | 0.82887 |
| P61514 | 60S ribosomal protein L37a | 0.02 | 0.830315 |
| Q3UJB9 | Enhancer of mRNA-decapping protein 4 | 0.02 | 0.833211 |
| Q9CRB8 | Mitochondrial fission process protein 1 | -0.01 | 0.83334 |
| Q8CG76 | Aflatoxin B1 aldehyde reductase member 2 | -0.01 | 0.833506 |
| Q8VCG4 | Complement component C8 gamma chain | 0.05 | 0.8336 |
| Q8R164 | Valacyclovir hydrolase | -0.04 | 0.833642 |
| Q61555 | Fibrillin-2 | -0.04 | 0.833901 |
| Q62523 | Zyxin | 0.02 | 0.833987 |
| Q91X72 | Hemopexin | 0.03 | 0.834448 |
| Q9ESB3 | Histidine-rich glycoprotein | -0.02 | 0.835779 |
| Q6P8X1 | Sorting nexin-6;Sorting nexin-6, N-terminally processed | 0.01 | 0.838591 |
| Q8BL03 | Mitochondrial basic amino acids transporter | -0.03 | 0.839055 |
| Q6PDN3 | Myosin light chain kinase, smooth muscle;Myosin light chain kinase, smooth muscle, deglutamylated form | -0.02 | 0.839152 |
| P21107 | Tropomyosin alpha-3 chain | -0.03 | 0.839439 |
| Q8BGQ7 | Alanine--tRNA ligase, cytoplasmic | 0.03 | 0.839571 |
| P62751 | 60S ribosomal protein L23a | 0.02 | 0.841744 |
| P09242 | Alkaline phosphatase, tissue-nonspecific isozyme | -0.02 | 0.842471 |
| Q8CC35 | Synaptopodin | 0.02 | 0.843452 |
| O54984 | ATPase Asna1 | -0.02 | 0.844407 |
| P15208 | Insulin receptor;Insulin receptor subunit alpha;Insulin receptor subunit beta | 0.02 | 0.845897 |
| Q9CZB0 | Succinate dehydrogenase cytochrome b560 subunit, mitochondrial | -0.03 | 0.846 |
| Q8CIZ8 | von Willebrand factor;von Willebrand antigen 2 | -0.02 | 0.846412 |
| Q8BGH2 | Sorting and assembly machinery component 50 homolog | 0.01 | 0.846511 |
| P82347 | Delta-sarcoglycan | 0.01 | 0.846656 |
| Q9CR86 | Calcium-regulated heat stable protein 1 | 0.03 | 0.846996 |
| P63030 | Mitochondrial pyruvate carrier 1 | -0.03 | 0.847147 |
| Q02819 | Nucleobindin-1 | 0.01 | 0.847781 |
| P23492 | Purine nucleoside phosphorylase | -0.02 | 0.849564 |
| P02468 | Laminin subunit gamma-1 | 0.01 | 0.849581 |
| O54901 | OX-2 membrane glycoprotein | 0.03 | 0.849649 |
| Q3THE2 | Myosin regulatory light chain 12B | 0.01 | 0.850514 |
| Q9QY93 | dCTP pyrophosphatase 1 | 0.03 | 0.851164 |
| Q61249 | Immunoglobulin-binding protein 1 | 0.02 | 0.851444 |
| Q921M3 | Splicing factor 3B subunit 3 | -0.02 | 0.853516 |
| Q921F4 | Heterogeneous nuclear ribonucleoprotein L-like | -0.03 | 0.853553 |
| Q00898 | Alpha-1-antitrypsin 1-5 | 0.03 | 0.855066 |
| Q791V5 | Mitochondrial carrier homolog 2 | -0.01 | 0.855454 |

| | | | |
|---|---|---|---|
| Q8C181 | Muscleblind-like protein 2 | -0.04 | 0.855802 |
| Q9D379 | Epoxide hydrolase 1 | 0.02 | 0.856073 |
| P56375 | Acylphosphatase-2 | 0.03 | 0.856216 |
| Q8VCW8 | Acyl-CoA synthetase family member 2, mitochondrial | -0.01 | 0.856458 |
| P51660 | Peroxisomal multifunctional enzyme type 2;(3R)-hydroxyacyl-CoA dehydrogenase;Enoyl-CoA hydratase 2 | -0.01 | 0.857709 |
| Q9JI91 | Alpha-actinin-2 | -0.01 | 0.858791 |
| Q8BH55 | Threonine synthase-like 1 | 0.01 | 0.859348 |
| Q60865 | Caprin-1 | 0.02 | 0.859869 |
| Q8BZ52 | Fibronectin type III and SPRY domain-containing protein 2 | 0.01 | 0.860412 |
| P63166 | Small ubiquitin-related modifier 1 | 0.04 | 0.862563 |
| O08784 | Treacle protein | -0.03 | 0.862992 |
| Q9D783 | Kelch-like protein 40 | 0.03 | 0.864491 |
| O08795 | Glucosidase 2 subunit beta | -0.02 | 0.865543 |
| Q9CX80 | Cytoglobin | -0.02 | 0.86572 |
| P63242 | Eukaryotic translation initiation factor 5A-1;Eukaryotic translation initiation factor 5A-2 | -0.01 | 0.866157 |
| Q8K1M6 | Dynamin-1-like protein | -0.01 | 0.867174 |
| Q9CQF0 | 39S ribosomal protein L11, mitochondrial | -0.02 | 0.867435 |
| P62849 | 40S ribosomal protein S24 | -0.01 | 0.867594 |
| Q9WUD1 | STIP1 homology and U box-containing protein 1 | 0.01 | 0.86775 |
| A2AGT5 | Cytoskeleton-associated protein 5 | -0.03 | 0.868457 |
| Q9D8E6 | 60S ribosomal protein L4 | -0.01 | 0.868554 |
| Q91WU5 | Arsenite methyltransferase | -0.03 | 0.870409 |
| P18406 | Protein CYR61 | 0.02 | 0.873189 |
| P62270 | 40S ribosomal protein S18 | 0.01 | 0.87367 |
| P20108 | Thioredoxin-dependent peroxide reductase, mitochondrial | 0.02 | 0.874152 |
| P09602 | Non-histone chromosomal protein HMG-17 | 0.02 | 0.875333 |
| Q62147 | Sarcospan | -0.02 | 0.875651 |
| Q9WV55 | Vesicle-associated membrane protein-associated protein A | 0.02 | 0.876288 |
| Q8VI36 | Paxillin | -0.02 | 0.877114 |
| Q8R2Y0 | Monoacylglycerol lipase ABHD6 | -0.03 | 0.877663 |
| Q9Z0H4 | CUGBP Elav-like family member 2 | -0.02 | 0.877777 |
| Q8BL66 | Early endosome antigen 1 | -0.01 | 0.878441 |
| Q8VDM4 | 26S proteasome non-ATPase regulatory subunit 2 | 0.01 | 0.879753 |
| P84099 | 60S ribosomal protein L19 | 0.01 | 0.880059 |
| P26443 | Glutamate dehydrogenase 1, mitochondrial | 0.01 | 0.880119 |
| Q61704 | Inter-alpha-trypsin inhibitor heavy chain H3 | 0.01 | 0.880475 |
| P62082 | 40S ribosomal protein S7 | 0.02 | 0.880595 |

| | | | |
|---|---|---|---|
| Q8BFZ9 | Erlin-2;Erlin-1 | -0.01 | 0.881831 |
| P13707 | Glycerol-3-phosphate dehydrogenase [NAD(+)], cytoplasmic | -0.01 | 0.882738 |
| P26262 | Plasma kallikrein;Plasma kallikrein heavy chain;Plasma kallikrein light chain | -0.01 | 0.883117 |
| P54775 | 26S protease regulatory subunit 6B | 0.01 | 0.8836 |
| Q6ZWR6 | Nesprin-1 | -0.01 | 0.88456 |
| P62918 | 60S ribosomal protein L8 | 0.01 | 0.885393 |
| Q99N95 | 39S ribosomal protein L3, mitochondrial | 0.01 | 0.885422 |
| Q9D938 | Transmembrane protein 160 | 0.02 | 0.887093 |
| P57780 | Alpha-actinin-4 | -0.01 | 0.888598 |
| Q64332 | Synapsin-2 | 0.02 | 0.88932 |
| P46471 | 26S protease regulatory subunit 7 | -0.01 | 0.89094 |
| Q921H9 | Cytochrome c oxidase assembly factor 7 | -0.01 | 0.89222 |
| Q8JZU0 | Nucleoside diphosphate-linked moiety X motif 13 | 0.03 | 0.894768 |
| P35980 | 60S ribosomal protein L18 | 0.01 | 0.896186 |
| P03953 | Complement factor D | -0.01 | 0.898184 |
| P61924 | Coatomer subunit zeta-1 | -0.02 | 0.898619 |
| Q6A065 | Centrosomal protein of 170 kDa | 0.02 | 0.899064 |
| Q60996 | Serine/threonine-protein phosphatase 2A 56 kDa regulatory subunit gamma isoform | 0.02 | 0.900249 |
| O35465 | Peptidyl-prolyl cis-trans isomerase FKBP8 | -0.02 | 0.901241 |
| Q8VCM8 | Nicalin | 0.06 | 0.901474 |
| Q922J3 | CAP-Gly domain-containing linker protein 1 | 0.01 | 0.901732 |
| Q3V4B5 | COMM domain-containing protein 6 | 0.02 | 0.903085 |
| Q91VR2 | ATP synthase subunit gamma, mitochondrial | -0.01 | 0.903919 |
| Q3UE37 | Ubiquitin-conjugating enzyme E2 Z | -0.01 | 0.904233 |
| P01863 | Ig gamma-2A chain C region, A allele;Ig gamma-2A chain C region secreted form;Ig gamma-2A chain C region, membrane-bound form | -0.04 | 0.904587 |
| Q9WV91 | Prostaglandin F2 receptor negative regulator | -0.01 | 0.905098 |
| P11688 | Integrin alpha-5;Integrin alpha-5 heavy chain;Integrin alpha-5 light chain | 0.03 | 0.905685 |
| Q9EPC1 | Alpha-parvin | -0.01 | 0.906257 |
| P09813 | Apolipoprotein A-II;Proapolipoprotein A-II | -0.02 | 0.906355 |
| P12815 | Programmed cell death protein 6 | -0.02 | 0.90787 |
| Q8BHG2 | UPF0587 protein C1orf123 homolog | -0.01 | 0.908407 |
| Q8VHE0 | Translocation protein SEC63 homolog | 0.04 | 0.908567 |
| Q91VS7 | Microsomal glutathione S-transferase 1 | 0.04 | 0.908672 |
| P68033 | Actin, alpha cardiac muscle 1 | 0.01 | 0.908926 |
| Q8CGB6 | Tensin-2 | 0.01 | 0.912331 |
| P97823 | Acyl-protein thioesterase 1 | 0.01 | 0.914089 |
| Q62351 | Transferrin receptor protein 1 | 0.01 | 0.916516 |
| P01872 | Ig mu chain C region | -0.02 | 0.917448 |

| | | | |
|---|---|---|---|
| Q3U7R1 | Extended synaptotagmin-1 | 0.01 | 0.917497 |
| Q9CXZ1 | NADH dehydrogenase [ubiquinone] iron-sulfur protein 4, mitochondrial | -0.01 | 0.918465 |
| P99026 | Proteasome subunit beta type-4 | 0.01 | 0.919129 |
| Q8BFW7 | Lipoma-preferred partner homolog | 0.01 | 0.921143 |
| O35343 | Importin subunit alpha-3 | 0.02 | 0.923765 |
| O54931 | A-kinase anchor protein 2 | 0.01 | 0.923842 |
| Q9CRD0 | OCIA domain-containing protein 1 | -0.01 | 0.923894 |
| Q91VX2 | Ubiquitin-associated protein 2 | 0.01 | 0.925609 |
| Q69Z28 | A disintegrin and metalloproteinase with thrombospondin motifs 16 | -0.01 | 0.925649 |
| Q3UPL0 | Protein transport protein Sec31A | 0.01 | 0.926205 |
| Q9JHW2 | Omega-amidase NIT2 | 0.01 | 0.926413 |
| Q9CQY5 | Magnesium transporter protein 1 | -0.01 | 0.92668 |
| Q8R123 | FAD synthase;Molybdenum cofactor biosynthesis protein-like region;FAD synthase region | 0.03 | 0.927594 |
| P62821 | Ras-related protein Rab-1A | 0.01 | 0.928862 |
| O70133 | ATP-dependent RNA helicase A | 0.01 | 0.929541 |
| Q9WV32 | Actin-related protein 2/3 complex subunit 1B | -0.01 | 0.930357 |
| P04247 | Myoglobin | 0.01 | 0.931441 |
| Q6PGF7 | Exocyst complex component 8 | 0.01 | 0.934595 |
| P47809 | Dual specificity mitogen-activated protein kinase kinase 4 | 0.01 | 0.935961 |
| P70158 | Acid sphingomyelinase-like phosphodiesterase 3a | 0.01 | 0.936458 |
| Q9QY80 | Very-long-chain (3R)-3-hydroxyacyl-CoA dehydratase 1 | 0.01 | 0.939979 |
| O55142 | 60S ribosomal protein L35a | 0.00 | 0.942707 |
| P62862 | 40S ribosomal protein S30 | 0.01 | 0.942708 |
| Q91YE8 | Synaptopodin-2 | -0.01 | 0.94296 |
| Q64314 | Hematopoietic progenitor cell antigen CD34 | -0.01 | 0.943158 |
| Q8BG32 | 26S proteasome non-ATPase regulatory subunit 11 | 0.00 | 0.943641 |
| Q9CQ40 | 39S ribosomal protein L49, mitochondrial | 0.01 | 0.944419 |
| P62806 | Histone H4 | 0.01 | 0.944894 |
| Q3TWW8 | Serine/arginine-rich splicing factor 6 | 0.00 | 0.945011 |
| P56695 | Wolframin | 0.01 | 0.945365 |
| Q8VE80 | THO complex subunit 3 | 0.18 | 0.946067 |
| O35206 | Collagen alpha-1(XV) chain;Restin | -0.01 | 0.946928 |
| Q9D8U8 | Sorting nexin-5 | -0.01 | 0.948951 |
| Q68FH4 | N-acetylgalactosamine kinase | -0.01 | 0.949414 |
| P58389 | Serine/threonine-protein phosphatase 2A activator | 0.00 | 0.949661 |
| P03888 | NADH-ubiquinone oxidoreductase chain 1 | 0.01 | 0.950372 |
| Q8BSY0 | Aspartyl/asparaginyl beta-hydroxylase | 0.01 | 0.95252 |
| Q8BPB0 | MOB kinase activator 1B | 0.00 | 0.952574 |

| | | | |
|---|---|---|---|
| Q9CPS6 | Histidine triad nucleotide-binding protein 3 | 0.00 | 0.952882 |
| Q9CX86 | Heterogeneous nuclear ribonucleoprotein A0 | 0.01 | 0.954041 |
| Q3U276 | Succinate dehydrogenase assembly factor 1, mitochondrial | 0.01 | 0.955414 |
| Q5F285 | Transmembrane protein 256 | -0.01 | 0.955536 |
| P52503 | NADH dehydrogenase [ubiquinone] iron-sulfur protein 6, mitochondrial | 0.00 | 0.955858 |
| Q70IV5 | Synemin | 0.01 | 0.955956 |
| Q9CR59 | Growth arrest and DNA damage-inducible proteins-interacting protein 1 | 0.01 | 0.956478 |
| Q9QUM9 | Proteasome subunit alpha type-6 | 0.00 | 0.956584 |
| P48036 | Annexin A5 | 0.00 | 0.956907 |
| Q3TFQ1 | SPRY domain-containing protein 7 | -0.01 | 0.957189 |
| Q60935 | GPI-linked NAD(P)(+)--arginine ADP-ribosyltransferase 1 | 0.00 | 0.95798 |
| Q8BTX9 | Inactive hydroxysteroid dehydrogenase-like protein 1 | 0.01 | 0.959127 |
| Q9WUQ2 | Prolactin regulatory element-binding protein | -0.01 | 0.959306 |
| G5E829 | Plasma membrane calcium-transporting ATPase 1 | 0.01 | 0.959452 |
| Q8VE97 | Serine/arginine-rich splicing factor 4 | 0.00 | 0.959579 |
| Q91VH6 | Protein MEMO1 | 0.00 | 0.959705 |
| P32067 | Lupus La protein homolog | 0.01 | 0.960152 |
| Q5HZI1 | Microtubule-associated tumor suppressor 1 homolog | 0.00 | 0.960191 |
| Q9DB73 | NADH-cytochrome b5 reductase 1 | 0.01 | 0.960314 |
| Q6PHZ2 | Calcium/calmodulin-dependent protein kinase type II subunit delta | 0.00 | 0.961998 |
| E9Q3L2 | Phosphatidylinositol 4-kinase alpha | 0.00 | 0.962084 |
| Q9DCN1 | Peroxisomal NADH pyrophosphatase NUDT12 | 0.00 | 0.962208 |
| P47811 | Mitogen-activated protein kinase 14 | -0.01 | 0.962659 |
| Q8K449 | ATP-binding cassette sub-family A member 9;ATP-binding cassette sub-family A member 6 | 0.00 | 0.9632 |
| Q91ZJ5 | UTP--glucose-1-phosphate uridylyltransferase | 0.00 | 0.963655 |
| P13439 | Uridine 5-monophosphate synthase;Orotate phosphoribosyltransferase;Orotidine 5-phosphate decarboxylase | -0.01 | 0.963872 |
| Q569Z6 | Thyroid hormone receptor-associated protein 3 | 0.00 | 0.965411 |
| O08709 | Peroxiredoxin-6 | 0.00 | 0.966215 |
| O35609 | Secretory carrier-associated membrane protein 3 | 0.01 | 0.967505 |
| Q9D2R6 | Cytochrome c oxidase assembly factor 3 homolog, mitochondrial | 0.00 | 0.967849 |
| Q8CAQ8 | MICOS complex subunit Mic60 | 0.00 | 0.972058 |
| Q8VHX6 | Filamin-C | 0.00 | 0.975136 |
| Q8K1J6 | CCA tRNA nucleotidyltransferase 1, mitochondrial | 0.00 | 0.975751 |
| O88531 | Palmitoyl-protein thioesterase 1 | 0.00 | 0.976307 |

| | | | |
|---|---|---|---|
| Q9D0S9 | Histidine triad nucleotide-binding protein 2, mitochondrial | 0.00 | 0.977177 |
| P70274 | Selenoprotein P | 0.00 | 0.977321 |
| Q6PDI6 | Protein FAM63B | -0.01 | 0.978102 |
| Q9WVA2 | Mitochondrial import inner membrane translocase subunit Tim8 A | 0.00 | 0.979717 |
| Q9D666 | SUN domain-containing protein 1 | 0.00 | 0.981716 |
| Q8BP67 | 60S ribosomal protein L24 | 0.00 | 0.982626 |
| P70372 | ELAV-like protein 1 | 0.00 | 0.982732 |
| Q5I043 | Ubiquitin carboxyl-terminal hydrolase 28 | -0.01 | 0.983049 |
| Q9ERG0 | LIM domain and actin-binding protein 1 | 0.00 | 0.985279 |
| Q8BGA9 | Mitochondrial inner membrane protein OXA1L | 0.00 | 0.985579 |
| Q9DCR2 | AP-3 complex subunit sigma-1 | 0.00 | 0.985639 |
| Q9EST5 | Acidic leucine-rich nuclear phosphoprotein 32 family member B | 0.00 | 0.985715 |
| Q99JI6 | Ras-related protein Rap-1b;Ras-related protein Rap-1A | 0.00 | 0.986284 |
| Q5SV42 | Leukocyte elastase inhibitor C;Leukocyte elastase inhibitor A;Leukocyte elastase inhibitor B | 0.00 | 0.986606 |
| Q920M5 | Coronin-6 | 0.00 | 0.988225 |
| Q99MS7 | EH domain-binding protein 1-like protein 1 | 0.00 | 0.988432 |
| P62983 | Ubiquitin-40S ribosomal protein S27a;Ubiquitin;40S ribosomal protein S27a;Ubiquitin-60S ribosomal protein L40;Ubiquitin;60S ribosomal protein L40;Polyubiquitin-B;Ubiquitin;Polyubiquitin-C;Ubiquitin;Ubiquitin-related 1;Ubiquitin-related 2 | 0.00 | 0.989069 |
| O08756 | 3-hydroxyacyl-CoA dehydrogenase type-2 | 0.00 | 0.991667 |
| Q9QYR9 | Acyl-coenzyme A thioesterase 2, mitochondrial | 0.00 | 0.991839 |
| P97384 | Annexin A11 | 0.00 | 0.991848 |
| P19157 | Glutathione S-transferase P 1 | 0.00 | 0.993275 |
| Q99KK9 | Probable histidine--tRNA ligase, mitochondrial | 0.00 | 0.993581 |
| P27659 | 60S ribosomal protein L3 | 0.00 | 0.99395 |
| P48678 | Prelamin-A/C;Lamin-A/C | 0.00 | 0.994309 |
| Q8BMF4 | Dihydrolipoyllysine-residue acetyltransferase component of pyruvate dehydrogenase complex, mitochondrial | 0.00 | 0.99448 |
| Q9R0Q7 | Prostaglandin E synthase 3 | 0.00 | 0.9947 |
| Q9ET54 | Palladin | 0.00 | 0.995577 |
| Q8R4U7 | Leucine zipper protein 1 | 0.00 | 0.996095 |
| Q810U5 | Coiled-coil domain-containing protein 50 | 0.00 | 0.996819 |
| Q5SUR0 | Phosphoribosylformylglycinamidine synthase | 0.00 | 0.99715 |
| Q4VAE3 | Transmembrane protein 65 | 0.00 | 0.997196 |
| Q8QZT1 | Acetyl-CoA acetyltransferase, mitochondrial | 0.00 | 0.997868 |
| Q99KR3 | Beta-lactamase-like protein 2 | 0.00 | 0.997933 |

| | | | |
|---|---|---|---|
| Q9DCV4 | Regulator of microtubule dynamics protein 1 | 0.00 | 0.997956 |
| Q91Z31 | Polypyrimidine tract-binding protein 2 | 0.00 | 0.99811 |
| Q8C6I2 | Succinate dehydrogenase assembly factor 2, mitochondrial | 0.00 | 0.998601 |

**Supplemental Table 1.** List of the 2907 proteins (including UniProt accessions and protein names) with relative fold change (FC) of expression between HFpEF and Sham mice (Log2 scale) and the associated P-value (ranked from lowest to highest).

| UNIPROT | PROTEIN | LogFC | P.VALUE | AMINO ACID |
|---|---|---|---|---|
| Q8K4G5 | Actin-binding LIM protein 1 | 0.88 | 0.000337 | S499 |
| Q8K4G5 | Actin-binding LIM protein 1 | 0.88 | 0.000366 | S496 |
| A2ASS6 | Titin | -1.01 | 0.002034 | S262 |
| A2ASS6 | Titin | -1.01 | 0.002034 | S264 |
| A2ASS6 | Titin | -1.01 | 0.002034 | T266 |
| Q91VN6 | Probable ATP-dependent RNA helicase DDX41 | 1.48 | 0.002044 | S21 |
| Q91VN6 | Probable ATP-dependent RNA helicase DDX41 | 1.48 | 0.002044 | S23 |
| Q9JLV1 | BAG family molecular chaperone regulator 3 | -1.30 | 0.002100 | S281 |
| Q9JLV1 | BAG family molecular chaperone regulator 3 | -1.30 | 0.002100 | S285 |
| Q99KL7 | Ras-related protein Rab-28 | 0.79 | 0.002248 | S2 |
| Q99KL7 | Ras-related protein Rab-28 | 0.79 | 0.002248 | S4 |
| Q91YE8 | Synaptopodin-2 | 0.77 | 0.003070 | S543 |
| E9Q401 | Ryanodine receptor 2 | 0.76 | 0.003790 | S2807 |
| E9Q401 | Ryanodine receptor 2 | 0.74 | 0.003799 | S2810 |
| E9Q401 | Ryanodine receptor 2 | 0.74 | 0.004747 | S2813 |
| P17095 | High mobility group protein HMG-I/HMG-Y | 2.27 | 0.004767 | S99 |
| P17095 | High mobility group protein HMG-I/HMG-Y | 2.27 | 0.004767 | S102 |
| P17095 | High mobility group protein HMG-I/HMG-Y | 2.27 | 0.004767 | S103 |
| O35295 | Transcriptional activator protein Pur-beta | 0.95 | 0.006992 | S6 |
| O35295 | Transcriptional activator protein Pur-beta | 0.95 | 0.006992 | S8 |
| Q62261 | Spectrin beta chain, non-erythrocytic 1 | -1.20 | 0.007246 | S2160 |
| Q62261 | Spectrin beta chain, non-erythrocytic 1 | -1.20 | 0.007246 | S2164 |
| Q62261 | Spectrin beta chain, non-erythrocytic 1 | -1.20 | 0.007246 | S2168 |
| Q9CQV4 | Protein FAM134C | 0.82 | 0.008424 | S258 |
| Q9QYG0 | Protein NDRG2 | 0.56 | 0.009887 | S350 |
| Q9QYR6 | Microtubule-associated protein 1A;MAP1A heavy chain;MAP1 light chain LC2 | 1.26 | 0.010896 | S1648 |
| Q9QYC0 | Alpha-adducin | 5.64 | 0.013701 | S600 |
| Q9QYC0 | Alpha-adducin | 5.64 | 0.013701 | T610 |
| Q9D0L8 | mRNA cap guanine-N7 methyltransferase | 0.88 | 0.015114 | S11 |
| Q9D0L8 | mRNA cap guanine-N7 methyltransferase | 0.88 | 0.015114 | S15 |
| Q9QYC0 | Alpha-adducin | 1.57 | 0.019358 | T610 |
| Q9QYC0 | Alpha-adducin | 1.57 | 0.019358 | T614 |
| Q9CQV4 | Protein FAM134C | 0.58 | 0.019363 | S313 |

| | | | | |
|---|---|---|---|---|
| **Q9CQV4** | Protein FAM134C | 0.58 | 0.019363 | S320 |
| **P59325** | Eukaryotic translation initiation factor 5 | 0.64 | 0.019505 | S387 |
| **P59325** | Eukaryotic translation initiation factor 5 | 0.64 | 0.019505 | S388 |
| **Q8K4G5** | Actin-binding LIM protein 1 | 0.54 | 0.020049 | S502 |
| **Q65CL1** | Catenin alpha-3 | 0.52 | 0.021709 | S637 |
| **Q65CL1** | Catenin alpha-3 | 0.52 | 0.021709 | S647 |
| **Q8N7N5** | DDB1- and CUL4-associated factor 8 | 1.90 | 0.021937 | S100 |
| **Q8K4G5** | Actin-binding LIM protein 1 | 1.10 | 0.022797 | S496 |
| **Q3TDQ1** | Dolichyl-diphosphooligosaccharide--protein glycosyltransferase subunit STT3B | -0.61 | 0.023072 | S495 |
| **Q3TDQ1** | Dolichyl-diphosphooligosaccharide--protein glycosyltransferase subunit STT3B | -0.61 | 0.023072 | S496 |
| **O54724** | Polymerase I and transcript release factor | -0.81 | 0.023906 | S38 |
| **O54724** | Polymerase I and transcript release factor | -0.81 | 0.023906 | S42 |
| **P62996** | Transformer-2 protein homolog beta;Transformer-2 protein homolog alpha | 0.48 | 0.025235 | S264 |
| **P62996** | Transformer-2 protein homolog beta;Transformer-2 protein homolog alpha | 0.48 | 0.025235 | S266 |
| **P50136** | 2-oxoisovalerate dehydrogenase subunit alpha, mitochondrial | 1.44 | 0.027331 | S334 |
| **Q9ET78** | Junctophilin-2 | 0.56 | 0.027335 | S600 |
| **Q69ZX8** | Actin-binding LIM protein 3 | 0.61 | 0.028424 | S282 |
| **Q69ZX8** | Actin-binding LIM protein 3 | 0.61 | 0.028424 | T279 |
| **Q9QXZ0** | Microtubule-actin cross-linking factor 1 | 1.00 | 0.029113 | S7296 |
| **Q9QYG0** | Protein NDRG2 | -0.46 | 0.029248 | S332 |
| **Q9ET78** | Junctophilin-2 | 0.55 | 0.031245 | S593 |
| **Q9ET78** | Junctophilin-2 | 0.55 | 0.031245 | S597 |
| **P23242** | Gap junction alpha-1 protein | 0.75 | 0.034897 | S306 |
| **A2ASS6** | Titin | -0.96 | 0.036700 | S34464 |
| **A2ASS6** | Titin | -0.96 | 0.036700 | T34467 |
| **P31324** | cAMP-dependent protein kinase type II-beta regulatory subunit | 1.86 | 0.036703 | S83 |
| **P31324** | cAMP-dependent protein kinase type II-beta regulatory subunit | 1.86 | 0.036703 | S85 |
| **Q9JKS4** | LIM domain-binding protein 3 | -0.86 | 0.041558 | S179 |
| **Q9JJW5** | Myozenin-2 | -0.51 | 0.041601 | T107 |
| **A2ASS6** | Titin | -0.54 | 0.043499 | S1411 |
| **A2ASS6** | Titin | -0.54 | 0.043499 | S1415 |
| **Q9QYG0** | Protein NDRG2 | 0.44 | 0.043940 | T348 |
| **Q8BR65** | Sin3 histone deacetylase corepressor complex component SDS3 | 0.61 | 0.044000 | S234 |
| **Q8BR65** | Sin3 histone deacetylase corepressor complex component SDS3 | 0.61 | 0.044000 | S236 |
| **Q9CQV4** | Protein FAM134C | 1.00 | 0.045517 | S260 |

| | | | | |
|---|---|---|---|---|
| Q9QYG0 | Protein NDRG2 | 0.81 | 0.047806 | S350 |
| P53986 | Monocarboxylate transporter 1 | 0.58 | 0.049050 | S491 |
| P83741 | Serine/threonine-protein kinase WNK1 | 0.55 | 0.049643 | S2024 |
| P83741 | Serine/threonine-protein kinase WNK1 | 0.55 | 0.049643 | S2027 |
| A2ASS6 | Titin | -0.41 | 0.052403 | S283 |
| A2ASS6 | Titin | -0.41 | 0.052403 | S290 |
| Q3UH68 | LIM and calponin homology domains-containing protein 1 | 0.82 | 0.058496 | S231 |
| A2ASS6 | Titin | 0.43 | 0.064976 | S34451 |
| A2ASS6 | Titin | 0.43 | 0.064976 | S34457 |
| O70548 | Telethonin | -0.36 | 0.067211 | S161 |
| Q8CI51 | PDZ and LIM domain protein 5 | -0.83 | 0.067862 | S228 |
| Q65CL1 | Catenin alpha-3 | 0.38 | 0.078774 | S650 |
| Q91YE8 | Synaptopodin-2 | 0.64 | 0.080662 | S319 |
| Q91YE8 | Synaptopodin-2 | 0.64 | 0.080662 | S320 |
| Q8BTI8 | Serine/arginine repetitive matrix protein 2 | 0.36 | 0.082986 | S2360 |
| Q8BTI8 | Serine/arginine repetitive matrix protein 2 | 0.36 | 0.082986 | T2362 |
| Q8BGD9 | Eukaryotic translation initiation factor 4B | -0.37 | 0.083874 | S498 |
| Q05BC3 | Echinoderm microtubule-associated protein-like 1 | -0.59 | 0.084235 | S139 |
| Q05BC3 | Echinoderm microtubule-associated protein-like 1 | -0.59 | 0.084235 | T132 |
| P48787 | Troponin I, cardiac muscle | 0.31 | 0.084899 | S24 |
| Q65CL1 | Catenin alpha-3 | 0.61 | 0.086437 | S637 |
| P48787 | Troponin I, cardiac muscle | 0.31 | 0.088784 | S23 |
| Q99JB8 | Protein kinase C and casein kinase II substrate protein 3 | -0.35 | 0.089916 | S354 |
| Q8K4G5 | Actin-binding LIM protein 1 | 0.36 | 0.094903 | S479 |
| Q8K4G5 | Actin-binding LIM protein 1 | 0.36 | 0.094903 | T473 |
| P28574 | Protein max | 0.81 | 0.095844 | S2 |
| P28574 | Protein max | 0.81 | 0.095844 | S11 |
| A2AQ25 | Sickle tail protein | 0.61 | 0.100992 | S1905 |
| P23242 | Gap junction alpha-1 protein | 0.44 | 0.101422 | S306 |
| P23242 | Gap junction alpha-1 protein | 0.44 | 0.101422 | S314 |
| A2ASS6 | Titin | 0.32 | 0.104988 | T33859 |
| P10637 | Microtubule-associated protein tau | 0.30 | 0.114052 | S494 |
| P20357 | Microtubule-associated protein 2 | 0.53 | 0.115786 | S626 |
| Q63918 | Serum deprivation-response protein | 0.43 | 0.118525 | S359 |
| Q63918 | Serum deprivation-response protein | 0.43 | 0.118525 | S363 |
| P26231 | Catenin alpha-1 | 0.47 | 0.121446 | S641 |
| Q9JJW5 | Myozenin-2 | -0.43 | 0.133272 | S101 |
| Q9JJW5 | Myozenin-2 | -0.43 | 0.133272 | S106 |
| Q9JJW5 | Myozenin-2 | -0.43 | 0.133272 | T107 |

| | | | | |
|---|---|---|---|---|
| Q9JJW5 | Myozenin-2 | -0.43 | 0.133272 | T111 |
| Q8BTI8 | Serine/arginine repetitive matrix protein 2 | 0.38 | 0.139875 | S453 |
| P23242 | Gap junction alpha-1 protein | 0.41 | 0.140356 | S314 |
| Q9JJW5 | Myozenin-2 | 0.28 | 0.141772 | S106 |
| Q9QYG0 | Protein NDRG2 | 0.32 | 0.152146 | S352 |
| Q9JJW5 | Myozenin-2 | -0.51 | 0.153059 | T111 |
| Q9R0P5 | Destrin | 0.32 | 0.154252 | S3 |
| Q9QYG0 | Protein NDRG2 | 0.61 | 0.161578 | T334 |
| E9Q401 | Ryanodine receptor 2 | 0.33 | 0.166243 | T2809 |
| P97825 | Hematological and neurological expressed 1 protein;Hematological and neurological expressed 1 protein, N-terminally processed | -0.36 | 0.167023 | T82 |
| Q6ZPQ6 | Membrane-associated phosphatidylinositol transfer protein 2 | 0.48 | 0.170086 | S338 |
| Q6AW69 | Cingulin-like protein 1 | 0.35 | 0.173505 | S199 |
| Q9ET78 | Junctophilin-2 | 0.36 | 0.180333 | S593 |
| Q9ET78 | Junctophilin-2 | 0.36 | 0.180333 | S597 |
| P10637 | Microtubule-associated protein tau | 0.37 | 0.183867 | S688 |
| O70548 | Telethonin | 0.58 | 0.184805 | S161 |
| O70548 | Telethonin | 0.58 | 0.184805 | S157 |
| Q9QYG0 | Protein NDRG2 | 0.29 | 0.189538 | S338 |
| A2ASS6 | Titin | -0.31 | 0.190039 | S34107 |
| Q8JZQ9 | Eukaryotic translation initiation factor 3 subunit B | 0.38 | 0.192130 | S68 |
| Q8JZQ9 | Eukaryotic translation initiation factor 3 subunit B | 0.38 | 0.192130 | S79 |
| Q63918 | Serum deprivation-response protein | 0.42 | 0.194510 | S363 |
| Q3UHX2 | 28 kDa heat- and acid-stable phosphoprotein | 0.29 | 0.195631 | S60 |
| Q3UHX2 | 28 kDa heat- and acid-stable phosphoprotein | 0.29 | 0.195631 | S63 |
| P48678 | Prelamin-A/C;Lamin-A/C | 0.90 | 0.196193 | S390 |
| Q9CT10 | Ran-binding protein 3 | 0.43 | 0.199658 | S33 |
| Q9CT10 | Ran-binding protein 3 | 0.43 | 0.199658 | S40 |
| P09528 | Ferritin heavy chain;Ferritin heavy chain, N-terminally processed | 0.51 | 0.201544 | S5 |
| REV__P84104 | Serine/arginine-rich splicing factor 3 | 0.28 | 0.202977 | S53 |
| Q62433 | Protein NDRG1 | 0.29 | 0.206554 | S330 |
| Q9ET78 | Junctophilin-2 | 0.80 | 0.208448 | S479 |
| Q9ET78 | Junctophilin-2 | 0.80 | 0.208448 | T483 |
| Q9JJW5 | Myozenin-2 | -0.34 | 0.210811 | S101 |
| Q52KI8 | Serine/arginine repetitive matrix protein 1 | 0.32 | 0.212506 | S779 |
| Q52KI8 | Serine/arginine repetitive matrix protein 1 | 0.32 | 0.212506 | S781 |

| | | | | |
|---|---|---|---|---|
| Q61234 | Alpha-1-syntrophin | -0.39 | 0.215172 | S194 |
| Q5U3K5 | Rab-like protein 6 | 0.84 | 0.224562 | S436 |
| Q5U3K5 | Rab-like protein 6 | 0.84 | 0.224562 | S438 |
| A2ASS6 | Titin | -0.33 | 0.225156 | S34109 |
| P26231 | Catenin alpha-1;Catenin alpha-2 | -0.33 | 0.233786 | S652 |
| P26231 | Catenin alpha-1 | -0.33 | 0.233786 | S641 |
| Q3B7Z2 | Oxysterol-binding protein 1 | 0.25 | 0.247092 | S188 |
| Q3B7Z2 | Oxysterol-binding protein 1 | 0.25 | 0.247092 | S191 |
| Q9QYG0 | Protein NDRG2 | -0.31 | 0.260330 | T330 |
| Q7TPW1 | Nexilin | -0.41 | 0.269016 | S495 |
| Q7TPW1 | Nexilin | -0.41 | 0.269016 | S500 |
| A2ASS6 | Titin | 1.13 | 0.270264 | S1406 |
| A2ASS6 | Titin | 1.13 | 0.270264 | S1408 |
| Q8BTI8 | Serine/arginine repetitive matrix protein 2 | 0.29 | 0.271569 | S2646 |
| Q8BTI8 | Serine/arginine repetitive matrix protein 2 | 0.29 | 0.271569 | S2648 |
| Q8BP27 | Swi5-dependent recombination DNA repair protein 1 homolog | 0.19 | 0.276966 | S67 |
| Q8BP27 | Swi5-dependent recombination DNA repair protein 1 homolog | 0.19 | 0.276966 | S71 |
| A2ASS6 | Titin | 0.46 | 0.288563 | Y33864 |
| Q8JZQ9 | Eukaryotic translation initiation factor 3 subunit B | 0.25 | 0.289285 | S120 |
| Q8JZQ9 | Eukaryotic translation initiation factor 3 subunit B | 0.25 | 0.289285 | S123 |
| Q9DCL8 | Protein phosphatase inhibitor 2 | -0.24 | 0.295392 | S122 |
| Q9DCL8 | Protein phosphatase inhibitor 2 | -0.24 | 0.295392 | S123 |
| Q8R1A4 | Dedicator of cytokinesis protein 7 | 0.33 | 0.298088 | S900 |
| P54116 | Erythrocyte band 7 integral membrane protein | -0.26 | 0.301210 | S2 |
| P54116 | Erythrocyte band 7 integral membrane protein | -0.26 | 0.301210 | S7 |
| Q8K4G5 | Actin-binding LIM protein 1 | 0.22 | 0.309350 | S475 |
| Q8K4G5 | Actin-binding LIM protein 1 | 0.22 | 0.309350 | T473 |
| Q8CHU3 | Epsin-2 | -0.40 | 0.313941 | S192 |
| Q8CHU3 | Epsin-2 | -0.40 | 0.313941 | S195 |
| A2ASS6 | Titin | -0.28 | 0.317909 | S34112 |
| P10637 | Microtubule-associated protein tau | 0.20 | 0.331846 | S491 |
| Q6ZQ58 | La-related protein 1 | 0.80 | 0.338291 | S743 |
| Q65CL1 | Catenin alpha-3 | 0.20 | 0.346186 | T649 |
| P58871 | 182 kDa tankyrase-1-binding protein | 0.18 | 0.351190 | S1612 |
| P58871 | 182 kDa tankyrase-1-binding protein | 0.18 | 0.351190 | S1622 |
| Q62407 | Striated muscle-specific serine/threonine-protein kinase | 0.27 | 0.352782 | S439 |

| | | | | |
|---|---|---|---|---|
| Q62407 | Striated muscle-specific serine/threonine-protein kinase | 0.27 | 0.352782 | T453 |
| Q9JJW5 | Myozenin-2 | -0.26 | 0.354268 | T107 |
| Q62407 | Striated muscle-specific serine/threonine-protein kinase | 0.23 | 0.356068 | S2451 |
| Q9QYG0 | Protein NDRG2 | 0.19 | 0.381123 | T330 |
| P48787 | Troponin I, cardiac muscle | 0.20 | 0.384793 | Y27 |
| Q8K4G5 | Actin-binding LIM protein 1 | -0.21 | 0.390800 | S470 |
| P48787 | Troponin I, cardiac muscle | 0.22 | 0.399341 | S24 |
| Q9QYG0 | Protein NDRG2 | -0.30 | 0.403147 | S326 |
| Q9QXS1 | Plectin | -0.26 | 0.405884 | S4393 |
| Q91YE8 | Synaptopodin-2 | 0.72 | 0.411212 | S319 |
| Q91YE8 | Synaptopodin-2 | 0.72 | 0.411212 | S320 |
| Q5XF90 | Probable cation-transporting ATPase 13A4 | 0.59 | 0.425462 | Y1146 |
| A2ASS6 | Titin | 0.19 | 0.431199 | S307 |
| Q4U4S6 | Xin actin-binding repeat-containing protein 2 | -0.31 | 0.437491 | S2913 |
| Q4U4S6 | Xin actin-binding repeat-containing protein 2 | -0.31 | 0.437491 | T2910 |
| O08582 | GTP-binding protein 1 | 0.14 | 0.444957 | S12 |
| Q6PDG5 | SWI/SNF complex subunit SMARCC2 | 0.15 | 0.458101 | S302 |
| Q3UH68 | LIM and calponin homology domains-containing protein 1 | -0.33 | 0.466491 | S233 |
| Q3UH68 | LIM and calponin homology domains-containing protein 1 | -0.33 | 0.466491 | S235 |
| Q69ZX8 | Actin-binding LIM protein 3 | 0.18 | 0.467522 | S372 |
| Q69ZX8 | Actin-binding LIM protein 3 | 0.18 | 0.467522 | S373 |
| P26231 | Catenin alpha-1;Catenin alpha-2 | -0.31 | 0.476647 | S652 |
| P26231 | Catenin alpha-1;Catenin alpha-2 | -0.31 | 0.476647 | S655 |
| P26231 | Catenin alpha-1;Catenin alpha-2 | -0.31 | 0.476647 | T654 |
| P26231 | Catenin alpha-1;Catenin alpha-2 | -0.31 | 0.476647 | T658 |
| P30999 | Catenin delta-1 | -0.22 | 0.481476 | S864 |
| O35295 | Transcriptional activator protein Pur-beta | -0.15 | 0.496962 | S8 |
| Q9QXS1 | Plectin | 0.12 | 0.503221 | S4393 |
| Q9QXS1 | Plectin | 0.12 | 0.503221 | S4396 |
| Q9QYG0 | Protein NDRG2 | 0.14 | 0.519701 | S338 |
| A2AB59 | Rho GTPase-activating protein 27 | 0.15 | 0.534236 | S462 |
| Q8K4G5 | Actin-binding LIM protein 1 | 0.14 | 0.556339 | S470 |
| Q8K4G5 | Actin-binding LIM protein 1 | 0.14 | 0.556339 | T477 |
| Q9QYG0 | Protein NDRG2 | 0.12 | 0.559702 | S332 |
| E9Q557 | Desmoplakin | 0.19 | 0.568066 | S2 |
| E9Q557 | Desmoplakin | 0.19 | 0.568066 | S7 |
| Q8K4G5 | Actin-binding LIM protein 1 | 0.11 | 0.568760 | S470 |
| Q9QYG0 | Protein NDRG2 | 0.13 | 0.572476 | T334 |

| | | | | |
|---|---|---|---|---|
| Q5F2E7 | Nuclear fragile X mental retardation-interacting protein 2 | -0.13 | 0.587004 | S213 |
| Q8BTI8 | Serine/arginine repetitive matrix protein 2 | -0.16 | 0.593347 | S2656 |
| Q8BTI8 | Serine/arginine repetitive matrix protein 2 | -0.16 | 0.593347 | S2660 |
| Q9Z0G2 | SRSF protein kinase 3 | 0.26 | 0.595819 | S328 |
| Q61029 | Lamina-associated polypeptide 2, isoforms beta/delta/epsilon/gamma;Lamina-associated polypeptide 2, isoforms alpha/zeta | -0.15 | 0.610134 | S66 |
| Q61029 | Lamina-associated polypeptide 2, isoforms beta/delta/epsilon/gamma;Lamina-associated polypeptide 2, isoforms alpha/zeta | -0.15 | 0.610134 | S67 |
| O35551 | Rab GTPase-binding effector protein 1 | -0.30 | 0.620491 | S410 |
| O70435 | Proteasome subunit alpha type-3 | -0.09 | 0.665599 | S250 |
| Q3UMU9 | Hepatoma-derived growth factor-related protein 2 | 0.08 | 0.678107 | S366 |
| Q3UMU9 | Hepatoma-derived growth factor-related protein 2 | 0.08 | 0.678107 | S367 |
| P14602 | Heat shock protein beta-1 | -0.18 | 0.686193 | S86 |
| Q9JKS4 | LIM domain-binding protein 3 | -0.09 | 0.690837 | S171 |
| Q9JKS4 | LIM domain-binding protein 3 | -0.09 | 0.690837 | S179 |
| Q8K4G5 | Actin-binding LIM protein 1 | -0.09 | 0.697985 | S479 |
| Q8K4G5 | Actin-binding LIM protein 1 | -0.09 | 0.697985 | T477 |
| Q9QYG0 | Protein NDRG2 | -0.07 | 0.702840 | S328 |
| Q9QYG0 | Protein NDRG2 | 0.08 | 0.708407 | S328 |
| Q62407 | Striated muscle-specific serine/threonine-protein kinase | 0.08 | 0.709313 | S439 |
| Q62407 | Striated muscle-specific serine/threonine-protein kinase | 0.08 | 0.709313 | S457 |
| Q62407 | Striated muscle-specific serine/threonine-protein kinase | 0.08 | 0.709313 | T453 |
| Q5U3K5 | Rab-like protein 6 | -0.11 | 0.729818 | S482 |
| Q5U3K5 | Rab-like protein 6 | -0.11 | 0.729818 | S483 |
| O35295 | Transcriptional activator protein Pur-beta | 0.08 | 0.730475 | S6 |
| Q3TLH4 | Protein PRRC2C | -0.11 | 0.732980 | S1219 |
| P48678 | Prelamin-A/C;Lamin-A/C | 0.06 | 0.751504 | S404 |
| P48678 | Prelamin-A/C;Lamin-A/C | 0.06 | 0.751504 | S407 |
| B2RUR8 | OTU domain-containing protein 7B | -0.12 | 0.764742 | S464 |
| B2RUR8 | OTU domain-containing protein 7B | -0.12 | 0.764742 | S467 |
| P61014 | Cardiac phospholamban | -0.16 | 0.784929 | S16 |
| P61014 | Cardiac phospholamban | -0.16 | 0.784929 | T17 |
| Q9QXS1 | Plectin | 0.15 | 0.787362 | S4396 |
| A2ASS6 | Titin | -0.07 | 0.792119 | S301 |

| | | | | |
|---|---|---|---|---|
| **A2ASS6** | Titin | -0.07 | 0.792119 | S307 |
| **A2ASS6** | Titin | -0.07 | 0.792119 | T299 |
| **Q3UH68** | LIM and calponin homology domains-containing protein 1 | 0.09 | 0.798587 | S217 |
| **Q8BWB1** | Synaptopodin 2-like protein | 0.05 | 0.813647 | S140 |
| **Q8BWB1** | Synaptopodin 2-like protein | 0.05 | 0.813647 | T138 |
| **P35486** | Pyruvate dehydrogenase E1 component subunit alpha, somatic form, mitochondrial | -0.08 | 0.839450 | S293 |
| **P35486** | Pyruvate dehydrogenase E1 component subunit alpha, somatic form, mitochondrial | -0.08 | 0.839450 | S300 |
| **Q9DBC7** | cAMP-dependent protein kinase type I-alpha regulatory subunit;cAMP-dependent protein kinase type I-alpha regulatory subunit, N-terminally processed | 0.06 | 0.847629 | S77 |
| **Q9DBC7** | cAMP-dependent protein kinase type I-alpha regulatory subunit;cAMP-dependent protein kinase type I-alpha regulatory subunit, N-terminally processed | 0.06 | 0.847629 | S83 |
| **Q6ZQ58** | La-related protein 1 | -0.06 | 0.850126 | S302 |
| **P26231** | Catenin alpha-1;Catenin alpha-2 | 0.05 | 0.868913 | S655 |
| **P26231** | Catenin alpha-1;Catenin alpha-2 | 0.05 | 0.868913 | T658 |
| **Q9JKS4** | LIM domain-binding protein 3 | 0.11 | 0.878356 | S171 |
| **Q8BGD9** | Eukaryotic translation initiation factor 4B | -0.04 | 0.884496 | S504 |
| **Q9ET78** | Junctophilin-2 | -0.05 | 0.886386 | S479 |
| **Q9ET78** | Junctophilin-2 | -0.05 | 0.886386 | T483 |
| **P53986** | Monocarboxylate transporter 1 | 0.03 | 0.888317 | S491 |
| **Q62407** | Striated muscle-specific serine/threonine-protein kinase | -0.03 | 0.906647 | S2019 |
| **Q62407** | Striated muscle-specific serine/threonine-protein kinase | -0.03 | 0.906647 | S2020 |
| **Q61234** | Alpha-1-syntrophin | -0.03 | 0.907064 | S183 |
| **Q61234** | Alpha-1-syntrophin | -0.03 | 0.907064 | S187 |
| **Q61234** | Alpha-1-syntrophin | -0.03 | 0.907064 | S194 |
| **Q62417** | Sorbin and SH3 domain-containing protein 1 | -0.05 | 0.914387 | S58 |
| **Q62417** | Sorbin and SH3 domain-containing protein 1 | -0.05 | 0.914387 | S62 |
| **P48787** | Troponin I, cardiac muscle | 0.02 | 0.921919 | S23 |
| **Q8BTI8** | Serine/arginine repetitive matrix protein 2 | 0.03 | 0.933465 | S1343 |
| **P26231** | Catenin alpha-1;Catenin alpha-2 | 0.02 | 0.938195 | S655 |
| **Q3U1Y4** | DENN domain-containing protein 4B | -0.01 | 0.958223 | S732 |
| **A2ASS6** | Titin | -0.02 | 0.976045 | S262 |
| **A2ASS6** | Titin | -0.02 | 0.976045 | S264 |
| **A2ASS6** | Titin | -0.02 | 0.976045 | T266 |
| **O54774** | AP-3 complex subunit delta-1 | 0.00 | 0.988799 | S760 |

| | | | | |
|---|---|---|---|---|
| **P61014** | Cardiac phospholamban | 0.00 | 0.997950 | S16 |

**Supplemental Table 2.** List of the 281 identified phosphorylation sites (including UniProt accessions, protein names and phosphorylation site) in HFpEF and Sham mice. Relative fold change (FC) of expression between HFpEF and Sham mice (Log2 scale) and the associated P-value (ranked from lowest to highest) are also listed.
